# The functional and catalytic landscape of urease reveals a conserved target against *Helicobacter pylori*

**DOI:** 10.1080/19490976.2026.2653575

**Published:** 2026-04-03

**Authors:** Qiang Song, Huimin Wu, Zhengcai Ma, Ting Huang, Xinhu Zhu, Zhipeng Zhang, Guicheng Wu, Rakia Manzoor, Shiyu Liu, Ye Wang, Xuegang Li, Wenjin Zhang, Xiaoli Ye, Hang Ma

**Affiliations:** a Engineering Research Center of Coptis Development and Utilization (Ministry of Education), School of Life Sciences, Southwest University, Chongqing, People's Republic of China; b Chongqing Municipality Clinical Research Center for Endocrinology and Metabolic Diseases, Chongqing University Three Gorges Hospital, Chongqing, People's Republic of China; c Engineering Research Center of Coptis Development and Utilization (Ministry of Education), College of Pharmaceutical Sciences, Southwest University, Chongqing, People's Republic of China

**Keywords:** *Helicobacter pylori*, urease, antibiotic resistance, mutation, gut microbiota

## Abstract

Nearly half of the global population is infected with *Helicobacter pylori*. Antibiotic use has led to substantial antimicrobial resistance and unintended gut microbiota depletion, creating an urgent need for alternative therapeutic strategies. Here, we demonstrate that urease, a key enzyme that enables *H. pylori* survival by hydrolysing urea to neutralize stomach acid, is a conserved antibacterial target with low risk of resistance development. Using comprehensive deep mutational scanning coupled with phage-based functional screening, we systematically evaluated how mutations in core residues affect urease expression, enzymatic activity, bacterial colonization, and virulence, uncovering the catalytic nature of *H. pylori* urease. We found exceptional evolutionary conservation within the urease catalytic pocket, and potential mutation sites that affect urease activity are not close to the core of this pocket. Analysis of existing urease inhibitors revealed that their binding sites are not typically in these potential mutation sites, which indicates that the potential for resistance development is low. In addition, we show that targeting urease alone is effective in eradicating *H. pylori* and synergistically boosts the efficacy of antibiotics. Notably, the incorporation of urease inhibitors into antibiotic-based therapeutic regimens effectively preserves gut microbiota diversity and microbial genomic stability, thereby lowering the risk of antibiotic resistance. Collectively, our study elucidate the inherent resistance-resistant property of urease and establish the clinical value of combining urease inhibitors with antibiotics to reduce antibiotic resistance.

## Graphical abstract

**Figure:**
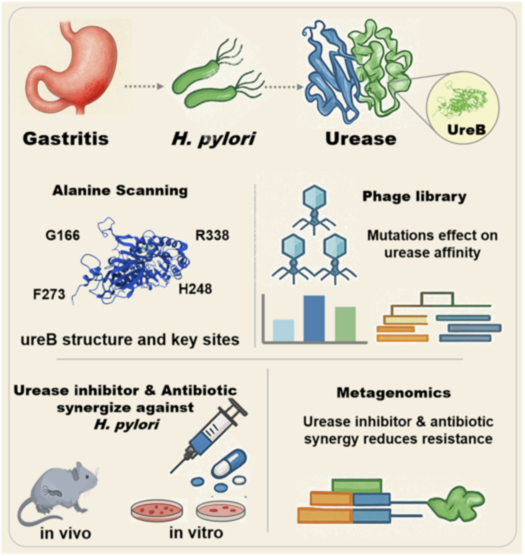

## Introduction

*Helicobacter pylori* (*H. pylori*) is a major pathogenic agent implicated in the etiology of chronic gastritis, peptic ulcer disease, gastric cancer, and gastric mucosa-associated lymphoid tissue lymphoma.^1^^,^^2^ Recent epidemiological data indicate that the global prevalence of *H. pylori* infection has reached 44.3%. Standard *H. pylori* eradication regimens rely heavily on combined antibiotic therapies involving amoxicillin, clarithromycin, amoxicillin, levofloxacin and metronidazole.^3^^,^^4^ However, the increasing resistance rates to these antibiotics have led to a marked decline in the clearance rates of standard therapies across numerous regions worldwide, thereby increasing the likelihood of treatment failure and recurrence.^4^^,^^5^

While pathogens employ transient resistance mechanisms, including reduced cell membrane permeability,^6^ biofilm formation,^7^ and efflux pump activation,^8^ sustained antibiotic resistance fundamentally requires genetic mutations in drug targets.^9^^,^^10^ This is consistently observed in clinically resistant strains. Amoxicillin resistance arises from mutations near the penicillin-binding motif in the *pbp1A* gene of *H. pylori.*^11^ More than 90% of clarithromycin-resistant *H. pylori* strains harbor mutations at 23S rRNA positions 2142-2143.^12^ Tetracycline resistance involves mutations at positions 926–928 of the 16S rRNA gene.^13^ Overcoming the limitations of current treatment frameworks and developing new therapeutic strategies that are less prone to genetic resistance have become imperative.

Urease plays an indispensable role in *H. pylori* colonization, survival, and pathogenicity.^14^ This enzyme hydrolyzes host-derived urea to produce ammonia and carbon dioxide. The generated ammonia creates an alkaline microenvironment that protects *H. pylori* from gastric acid, allowing it to survive in the harsh stomach environment.^15^^,^^16^ Moreover, ammonia directly damages gastric mucosal cells through its toxic effects. Urease also works with TlpB to detect urea signals from the gastric epithelium, guiding bacterial chemotaxis towards gastric pits and promoting colonization.^17^ Therefore, urease is both fundamentally required for *H. pylori* survival and a key virulence factor that facilitates infection and gastric disease development. By inhibiting urease activity, urease inhibitors eliminate the protective alkaline niche and disrupt chemotaxis, thereby reducing *H. pylori* colonization and growth.^18^^,^^19^ On the basis of this insight, precise inhibition of urease activity has been shown to have direct and effective antibacterial potential.

Numerous urease inhibitors have been developed and classified into three groups according to their mechanism of action. The first group comprises urea analogs, including thiourea and urea derivatives.^20^ The second group consists of nonurea analog inhibitors, such as phosphorodiamidates and imidazoles, that bind to the catalytic site despite their structural differences from urea.^21^ The third group includes metal chelators, such as acetohydroxamic acid (AHA), that inactivate the enzyme by sequestering nickel ions. Although inhibitors such as AHA are clinically approved, their therapeutic application remains limited because urease is expressed in only a limited number of microorganisms and therefore cannot be broadly applied for the treatment of microbial infections.^19^^,^^22^^,^^23^ Therefore, comprehensive knowledge of how urease inhibitors impact the *H. pylori* would aid efforts to understand *H. pylori* evolution and guide the design of drug resistance and other countermeasures.

This study systematically investigates the biological functions of *H. pylori* urease and evaluates the therapeutic potential of urease inhibitors against *H. pylori* infection. We demonstrate that urease inhibitors have a low tendency to induce bacterial resistance. Through mutagenesis studies, we identify critical amino acid residues that influence urease activity and define specific catalytic motifs required for urea hydrolysis, providing a molecular basis for understanding its mechanism. Furthermore, we analyze the binding characteristics of currently available urease inhibitors to elucidate the mechanisms by which they avoid specific anti-urease resistance. Importantly, we discover synergistic activity between urease inhibitors and conventional antibiotics. These findings reveal the functional molecular architecture of urease and may contribute to the development of safer, more effective, and practical treatment strategies for *H. pylori* eradication.

## Materials and methods

### Chemicals and reagents

Columbia solid and liquid culture media were procured from Qingdao Highborn Biotechnology (HB8646, Qingdao, China) and Thermo Scientific (CM0331B, Waltham, Massachusetts, USA), respectively. The GES-1 cell line was obtained from the Cell Bank of the Type Culture Collection of the Chinese Academy of Sciences (Shanghai, China). Enzyme-linked immunosorbent assay (ELISA) kits for mouse interleukin (IL)-1β (catalog number ml098416), IL-6 (catalog number ml098430), and TNF-alpha (catalog number ml002095) were sourced from Enzyme-linked Biotechnology Corporation (Shanghai, China). Additionally, a mouse *H. pylori* antibody IgG ELISA kit (catalog number KA0220) was acquired from Wuhan Aimeijie Technology Co., Ltd (Wuhan, China) to quantify anti-*H. pylori* IgG levels. Primary antibodies for UreB (catalog number ab127916), 6× His-tag (catalog number ab200537), ZO-1 (catalog number ab307799), CD45 (catalog number ab40763), IL-6 (catalog number ab233706), and TNF-alpha (catalog number ab307165) were purchased from Abcam (Cambridge, MA, USA). The *H. pylori* stool antigen (HPSA) was procured from Botuo Biotechnology Co., Ltd (Hangzhou, China). Cell culture reagents, including RPMI 1640 medium (11875119), penicillin/streptomycin (15140122), Trypsin-EDTA (25300120), and fetal bovine serum (FBS, A5670701), were obtained from Invitrogen (Thermo Fisher Scientific, Inc.). The MiniBEST Bacteria Genomic DNA Extraction Kit (9770Q) was procured from TaKaRa Bio-Inc., Japan. The pBluescript II KS (-) Plasmid was obtained from Wuhan Miaoling Biotechnology Co., Ltd, located in Wuhan, China. Additionally, the Urease Activity Detection Kit (BC4115) was acquired from Solaibao, based in Beijing, China.

### Amino acid sequence homology alignment

The amino acid sequences of UreB from various strains were downloaded from the UniProt database (www.uniprot.org). After the sequences were converted to FASTA format, sequence alignment was conducted using ClustalX software. The “Do Complete Alignment” function was employed to generate an output file with the “.aln” suffix. BOXSHADE was subsequently utilized for comparative analysis. Amino acid sequences are shown in Supplementary Table 1.

### H. pylori strains and growth conditions

The standard *H. pylori* strain (ATCC 43504) was procured from Biobw Biotechnology Co., Ltd. (Beijing, China) and its identity was confirmed through 16S rRNA sequencing conducted by the Beijing Genomics Institute. The *H. pylori* bacteria were cultured on brain-heart infusion agar plates supplemented with 10% sheep blood under microaerophilic conditions at 37 °C (comprising 5% O_2_, 10% CO_2_, and 85% N_2_). Once colonies developed on the solid medium, representative colonies were selected and inoculated into a liquid medium containing 10% fetal bovine serum (FBS). These cultures were then incubated on a shaker at 120 RPM for 24 h to reach the logarithmic growth phase, facilitating further experimental analysis. For long-term storage, *H. pylori* strains are typically preserved at –80 °C in a solution containing 25% glycerol.

### H. pylori growth curve analysis

The turbidity of the *H. pylori* culture in the logarithmic growth phase was adjusted to an optical density of 0.05 using the appropriate culture medium. Sterile 6-well plates were prepared, and 2 ml of the bacterial suspension was added to each well. An equivalent volume of blank medium containing DMSO was added to the wells designated as negative controls. Following the loading of the samples, the well plates were placed in a tri-gas incubator for shaking culture. At predetermined time points, i.e., 0, 6, 12, 18, 24, and 30 h, 100 μl of bacterial solution was quantitatively aspirated, and the absorbance at 600 nm (OD600) was measured using a microplate reader. Each concentration was tested in triplicate, and the entire experiment was conducted three times. A growth curve was constructed with the sampling time points on the x-axis and the corresponding OD600 values on the y-axis.

### Determination of MIC and MBC

The Minimum Inhibitory Concentration (MIC) of antibiotics was assessed using the broth microdilution method. Antibiotic solutions were prepared in microtiter plates following the two-fold serial dilution technique. *H. pylori* in its logarithmic growth phase was introduced to achieve a final bacterial concentration of 1.5 × 10^8^ CFU/mL. The plates were incubated for 24 h at 37 °C under conditions of 5% oxygen and 10% carbon dioxide. The MIC value was identified as the lowest antibiotic concentration that visibly inhibited bacterial growth. Subsequently, the Minimum Bactericidal Concentration (MBC) was determined based on the MIC samples. Following MIC determination, 50 µL of each culture broth, ranging from 1 to 4 times the MIC, was plated on solid media and incubated for three days under the same conditions. The MBC was defined as the lowest concentration of the antibiotic that resulted in a 99.99% reduction in bacterial viability, as per CLSI guidelines (2012).^24^

### Drug-resistant H. pylori induction

To simulate the clinical process of antibiotic resistance, under acidic (pH 4.5) conditions and 5 mM urea, bacteria were continuously exposed to liquid medium containing 1/2× MIC of compounds. First, the MICs of metronidazole, levofloxacin, EPI, and AHA against ATCC 43504 were determined. *H. pylori* ATCC 43504 was subsequently incubated separately on liquid medium supplemented with subinhibitory concentrations of metronidazole, levofloxacin, EPI, and AHA. The reasons we selected EPI and AHA as urease inhibitors are, on one hand, their high inhibitory activity against urease,^25-28^ and on the other hand, the substantial preliminary work accumulated by our team.^25^ After 24 h of incubation, samples containing *H. pylori* were coated on solid medium. Following the development of colonies on the solid agar, the bacteria were subsequently inoculated back into the liquid medium. The MIC was determined by the serial twofold dilution method every passage. When the MIC value increased, the drug concentration on the solid medium was updated accordingly. This process was repeated for 40 generations of ATCC43504. Detection of the candidate mutations was performed by direct sequencing. The primers used for the detection of mutations are listed in Supplementary Table 2.

### qRT-PCR

Total RNA was isolated utilizing the phenol-chloroform extraction method. The RNA concentration was quantified using a NanoDrop One spectrophotometer (Thermo Fisher Scientific, Massachusetts, USA). Subsequently, 1 µg of RNA was reverse transcribed into complementary DNA (cDNA) using the Takara reverse transcription kit. Quantitative real-time PCR (qRT-PCR) analyzes were conducted on the T100^TM^ Thermal Cycler (Bio-Rad Laboratories). The specific primers employed in these experiments are detailed in Supplementary Table 2. The qRT-PCR procedures were executed in accordance with the Minimum Information for Publication of Quantitative Digital PCR Experiments (MIQE) guidelines.^29^

### Transmission electron microscopy (TEM)

The impact of bacterial resistance on the micromorphology of *H. pylori* was investigated using transmission electron microscopy (TEM; Hitachi model H-7650, Japan). The study included a control group as well as 20th and 40th generations of drug-resistant bacteria, which were cultured on solid medium. Following the growth of monoclonal bacteria, the backward-picking bacteria were transferred to liquid medium for further culture. The bacterial samples were washed twice with phosphate-buffered saline (PBS) and subsequently fixed with 2.5% glutaraldehyde overnight. The samples were then postfixed with 1% osmium tetroxide (OsO_4_) and dehydrated in a series of graded ethanol solutions. Ultimately, the samples were embedded in Spurr resin, sectioned, and stained with uranyl acetate. Ultrastructural analysis was conducted using TEM.

### Transcriptomic analysis

Transcriptomic sequencing was utilized to systematically assess the effects of EPI and AHA on the transcriptional changes of *H. pylori*. Samples were collected from both untreated *H. pylori* and those treated with EPI and AHA at half the minimum inhibitory concentration (1/2× MIC). Transcriptome sequencing (single-end 50 bp, SE50) was performed using the BGISEQ-500 platform by BGI Genomics Co., Ltd., with three biological replicates for each condition. Raw sequencing data were processed to obtain clean data by removing reads containing adapters, reads with ambiguous bases (*N*), and low-quality reads. The processed reads were then aligned to the ATCC 43504 reference genome using Bowtie2 version 2.2.3. Differential expression analysis was conducted using DESeq; statistical methods based on the negative binomial distribution were employed to identify differentially expressed genes (DEGs). Genes were considered significantly differentially expressed if they exhibited a false discovery rate (FDR) of ≤0.05 and an absolute log fold change (|logFC|) of ≥2, and these genes were selected for subsequent analysis.

### Detection of metabolites by HPLC

*H. pylori* was treated with 1/2× MIC EPI or AHA. Bacterial culture media were collected at 1 h 12 h, and 24 h. The supernatant and bacterial cells were separated by centrifugation at 6000 rpm. The bacterial pellet was resuspended in PBS and lysed using a generator disruptor, after which the lysates were collected. An equal volume of acetonitrile was added to both the supernatant medium and the lysate, and the mixture was agitated overnight at 4 °C. Cellular debris was removed by centrifugation at 12000 rpm, and the supernatant was subsequently filtered through a 0.22 µm filter to eliminate protein aggregates. The metabolites of EPI and AHA were analyzed using high-performance liquid chromatography (HPLC).

### Drug sensitivity recovery

Fortieth-generation drug-resistant bacteria were resuspended in solid culture dishes without drugs. These bacteria were initially cultivated in an acidic liquid medium with a pH of 4.5, and the MIC was assessed after a 24-hour period of growth stabilization. The bacteria were subsequently transferred to a solid medium, which was maintained at a pH of 4.5. Single bacterial colonies were then isolated from the solid medium and further cultured in the liquid medium. This process was repeated continuously over 30 generations.

### Adhesion of bacteria to gastric epithelial cells

Gastric epithelial cells (GES-1 cells) were cultured in RPMI 1640 medium supplemented with 10% heat-inactivated fetal bovine serum (FBS) and 1% penicillin‒streptomycin and maintained in an incubator at 37 °C with 5% CO_2_. GES-1 cells (5 × 10^4^) in the logarithmic growth phase were seeded into 6-well plates and cultured for 24 h until they reached 50% to 60% confluency. The standard culture medium was aspirated, and acidic cell culture medium containing 5 mM urea was added, followed by the introduction of *H. pylori* in the logarithmic growth phase at a multiplicity of infection of 100. After a 2-hour coculture period, the medium was gently aspirated, and the cells were washed six times with sterile PBS to remove excess *H. pylori*. The cells were then digested with trypsin and counted. A total of 1 × 10^5^ cells were taken and serially diluted in *H. pylori* liquid medium at dilutions of 1 × 10^−1^, 1 × 10^−2^, 1 × 10^−3^, 1 × 10^−4^, 1 × 10^−5^, and 1 × 10^−6^. Subsequently, 20 µl of each dilution was plated onto solid Petri dishes in triplicate. After 72 h, once the colony growth was complete, the number of colonies at the 1 × 10^−4^ dilution was counted using an electron microscope. The number of colonies was used to assess the relative adhesion ability of the bacteria.

### UreB knockout

Bacterial DNA was extracted using a Takara bacterial DNA extraction kit. The genomic DNA served as a template for the amplification of the upstream and downstream homologous arms of the *UreB* gene. Concurrently, a fragment of the *Kana* gene was amplified from the pBluescript II KS (-) plasmid. The anterior and posterior homologous arms of the *UreB* gene, along with the *Kana* gene, were subsequently ligated and amplified into a single fragment using overlapping extension PCR. The resulting amplified fragment was subsequently ligated into the pBluescript II KS (-) plasmid, which was subsequently introduced into wild-type *H. pylori* via electroporation. For this process, the 363–1105 bp segment of the UreB gene was replaced with the *Kana* fragment. Positive bacterial colonies were screened on solid culture medium containing kanamycin to ensure resistance. Knockout of the *UreB* gene was confirmed through Sanger sequencing. The specific primers utilized for gene knockout are detailed in Supplementary Table 2.

### H. pylori point mutation construction

The target fragments were initially amplified using primers designed to incorporate a full-length 6× His-tag, with primer binding sites positioned on both sides of the insertion site. The amplified DNA fragments were ligated using splicing by overlap extension PCR. The recombinant fragments were subsequently ligated into the pBluescript II KS (-) plasmid and introduced into wild-type *H. pylori* via electroporation. Mutated bacteria were identified through monoclonal screening and sequencing. Kanamycin resistance and promoter fragments were introduced into the standard ATCC43504 strain containing the *UreB* gene using the same method as described above.

Finally, specific primers were designed to amplify the selected fragments by overlapping extension PCR using strains containing the kanamycin fragments as a template. The resulting product was inserted into the pBluescript II KS (-) plasmid through ligation, and electroporation into *H. pylori* was subsequently conducted using the previously constructed 6× His-tag. The primers used for point mutation are detailed in the supplementary materials (Supplementary Table 2).

### Western blot

Proteins from *H. pylori* were extracted utilizing the Protein Extraction Reagent (catalog number 78510; ThermoFisher, Carlsbad, CA, USA) in conjunction with phenylmethylsulfonyl fluoride. The protein concentration was subsequently determined using the BCA Protein Assay Reagent (Beyotime, China). 20 µg protein samples were separated by SDS–PAGE, and then transferred to a polyvinylidene difluoride membrane (Bio-Rad, Hercules, CA, USA). The membrane was blocked with 5% skim milk and then incubated with UreB polyclonal antibody (ab127916, Abcam, Cambridge, MA, USA) overnight at 4 °C and secondary antibody for 2 h at room temperature. Immunoreactivity was measured using ECL substrate (Epizyme Biotech, China) and images were captured using a chemiluminescence imaging system (Chemstudio SA2, Analytik Jena, Germany).

### Urease activity measurement

The urease activity of all the point mutation-containing bacteria was investigated. Bacterial suspension turbidity was measured using a Maxwell turbidity meter. Bacteria (3 × 10^9^ CFU) were then collected by centrifugation at 6000 rpm for 5 min. The supernatant was discarded, and the remaining bacteria were resuspended in 300 µL of PBS. This solution was then ultrasonicated (pulse on for 3 sec, pulse off for 10 sec; power, 200 W) using an ultrasonic processor and centrifugation at 12,000 rpm for 30 min at 4 °C. The resulting supernatant was collected and promptly stored at –80 °C. A urease activity detection kit was used according to the manufacturer's instructions to assess urease activity. Each experimental group comprised three independent protein samples (*n* = 3), with three replicates for each sample.

### Animal experiments

All procedures related to animal care and use were approved by the Animal Ethics Committee of Three Gorges Hospital, which is affiliated with Chongqing University (SYXXWD2024-063). Specific-pathogen-free (SPF) C57BL/6 female mice, aged 7 weeks, were procured from Sibeifu Biotechnology (Beijing, China). The mice were maintained under controlled environmental conditions (temperature: 25 ± 2 °C; relative humidity: 50% ± 5%; 12-hour light/dark cycle) and were provided with ad libitum access to food and sterilized water. Following a one-week acclimatization period, the mice were randomly assigned to two groups: a control group and a model group. The mice in the model group were infected with *H. pylori* (1 × 10^10^ CFU/0.2 mL/mouse) daily for seven consecutive days. Diet and water were withheld for 4 h post-inoculation. The control group received broth medium instead of bacterial inoculum. Two weeks after the initial *H. pylori* inoculation, stool antigen tests were conducted to confirm the successful colonization of *H. pylori* in the stomachs of the mice.

The mice infected with *H. pylori* were subsequently randomly assigned to different treatment groups. The infected mice underwent EPI-based combination therapy consisting of EPI (150 mg/kg/day) coadministered with either tetracycline (150 mg/kg/day) or clarithromycin (130 mg/kg/day) for 14 consecutive days. The dosage and treatment duration were determined according to the fifth Chinese expert consensus on clinical equivalent dosing.^30^^,^^31^ The control and model groups received an equivalent volume of saline instead. One week after the final drug treatment, the *H. pylori* stool antigen (HPSA) test and a mouse *H. pylori* antibody *IgG* ELISA kit were used to assess the eradication efficacy of the drug. Prior to euthanasia, the animals were fasted overnight. Their stomachs were then excised along the greater curvature, with a small portion allocated for solid culture dish cultivation and the remainder fixed in 4% paraformaldehyde for subsequent analyzes.

### Cultivation of H. pylori from mouse gastric tissue

To culture *H. pylori* from mouse gastric tissues, selective medium (Dent’s medium with 20 μg/mL bacitracin and 10 μg/mL nalidixic acid) and a blood agar base were used. Mouse gastric biopsy samples were weighed, homogenized for 10–20 sec in 1.5 mL tubes using a hand homogenizer, and subjected to serial dilution in sterile PBS. Afterwards, 100 μL of each dilution was added and incubated under microaerophilic conditions for 5 d. *H. pylori* colonies were identified via Gram staining and rapid tests, with bacterial counts expressed as colony-forming units (CFUs) per gram of tissue.

### Enzyme-linked immunosorbent assay (ELISA)

Blood samples were collected from the mouse vena cava, placed in clean tubes and incubated at RT for 1 h. The samples were subsequently centrifuged at 3000 rpm at 4 °C for 10 minutes to separate the serum from the supernatant. A complete ELISA kit for the detection of IL-1beta, IL-6, and TNF-*α* was then brought to room temperature for two hours. The concentrations of IL-1beta, IL-6, and TNF-*α* were determined with the kit according to the manufacturer’s protocol.

### Histological analysis of gastric tissue sections

Haematoxylin and eosin (H&E) staining was employed to examine histological alterations in gastric tissues. The tissues were initially fixed in paraformaldehyde and subsequently embedded in paraffin wax blocks. Afterwards, the tissues were cut into consecutive sections with a thickness of 5 μm. All the sections were then dewaxed, rehydrated, and stained using an H&E staining kit (G1076; Sevier Biological Technology Co., Ltd., Wuhan) in accordance with the manufacturer's specifications.

Immunohistochemistry (IHC) was used to measure the expression of IL-6 and TNF-ɑ. Paraffin-embedded tissue sections were dewaxed and rehydrated, followed by antigen retrieval at elevated temperatures using sodium citrate buffer. Endogenous peroxidase activity was inhibited by treatment with 3% hydrogen peroxide (H_2_O_2_), and nonspecific binding was minimized through blocking with 5% normal goat serum. A polyclonal antibody was utilized for IHC staining, followed by the application of a horseradish peroxidase (HRP)-conjugated anti-IgM secondary antibody (SA5-10288; Invitrogen, USA) at a 1:1000 dilution. The sections were subsequently incubated with streptavidin-HRP (Neobioscience; Shenzhen, China), followed by staining with 3, 3'-diaminobenzidine. The sections were then counterstained with haematoxylin and mounted using neutral gum.

Immunofluorescence was used to measure the expression of ZO-1 and CD45. Tissues were deparaffinized and dehydrated through a series of graded alcohol solutions, followed by antigen retrieval. The tissues were subsequently incubated with primary antibodies overnight at 4 °C. This was followed by a 1-hour incubation with FITC-labeled anti-rabbit secondary antibodies (GB22404; Servicebio). Nuclear staining was performed using 4’,6-diamidino-2-phenylindole. Images were subsequently obtained using a fluorescence microscope (BX60, Olympus, Japan).

### Molecular docking

The crystal structure of the UreB protein utilized for the docking studies was obtained from the Protein Data Bank (PDB) (http://www.rcsb.org/pdb/) with the PDB ID 6zja. Water molecules and small-molecule ligands were subsequently removed using PyMOL version 2.5.5. The three-dimensional structures of the small-molecule compounds were retrieved from the PubChem database (https://pubchem.ncbi.nlm.nih.gov/) and subjected to energy minimization by employing the MMFF94 force field. Molecular docking was conducted using AutoDock Vina version 1.2.3. Finally, PyMOL version 2.5.5 was employed to visualize the docking results.

### Purification of the UreB protein

The full-length or partial *UreB* gene was amplified using extracted *H. pylori* DNA as the template. A Flag tag was introduced at the *N*-terminus of *UreB*. Full-length *UreB* was ligated to an XhoI-digested PT7 vector. The resulting plasmid was subsequently transformed into the *Escherichia coli* strain BL21. Single bacterial colonies were selected and inoculated into Luria–Bertani (LB) liquid medium containing kanamycin for primary culture at 37 °C with agitation at 180 rpm. Upon reaching an OD600 value of 0.6–0.7 in the culture medium, a secondary cultivation was initiated with an inoculation volume of 1:100. When the OD600 of the secondary culture reached 0.5–0.6, protein expression was induced by adding isopropyl β-D-1-thiogalactopyranoside (IPTG) to a final concentration of 1 mmol/L, followed by incubation at 25 °C for 4 h. Subsequently, the induced bacterial cells were harvested by centrifugation at 4 °C and 4000 rpm for 25 minutes, after which the supernatant was carefully removed. The cell pellet was resuspended in equilibration buffer and vigorously vortexed to ensure complete homogenization. The cell suspension was supplemented with phenylmethylsulfonyl fluoride (PMSF) as a protease inhibitor and lysozyme (final concentration of 1 mg/mL), followed by incubation at 37 °C for 20 min to achieve complete cell lysis. Subsequent cell disruption was performed using an ultrasonic cell disruptor, with a total treatment time of 25 minutes, employing a 3-second pulse followed by a 7-second interval. The lysate was clarified by centrifugation at 12,000 rpm for 15 min at 4 °C, and the resulting supernatant containing soluble proteins was carefully collected.

### Biotinylated probe synthesis

3-Hydroxy-4-methoxybenzaldehyde, molecular sieve 5 A, and ammonium acetate were accurately measured and added to a round-bottom flask. Next, 100 mL of acetic acid and 13 mL of nitromethane were added to the reaction mixture. The reaction mixture was stirred under reflux for 8 h, after which the reaction progress was monitored by thin-layer chromatography (TLC). Afterwards, the slurry was filtered over diatomaceous earth, followed by the addition of ethyl acetate. The reaction mixture was then heated to 80 °C and stored at –20 °C to allow crystallization. The solution was filtered through a 0.22 μm filter and vacuum dried to obtain Compound 2. The compound was dissolved in tetrahydrofuran and methanol, and sodium borohydride was added at 0 °C. TLC was used to monitor the reaction progress. The mixture was poured into ice-cold water and acidified with dilute HCl to pH 7.0. Compound 3 was purified by column chromatography on silica gel. It was then dissolved in ethyl acetate, after which hydrochloric acid solution was added. The mixture was then heated to 60 °C, and TLC was used to obtain Compound 4. Compound 4 was extracted with dichloromethane and dried with anhydrous sodium sulfate. Finally, the dichloromethane was evaporated to yield Compound 5. Compound 5 was dissolved in dichloroethane, reacted with sodium triacetoxyborohydride, and monitored by TLC. Afterwards, the solvent was evaporated to dryness and subjected to column chromatography, which yielded Compound 6. Compound 6 was stirred with dichloromethane under argon. Compound 7 was purified by precipitation in cold diethylether, filtered, and dried under vacuum at 30 °C. A mixture of Compound 7 and K_2_CO3 was magnetically stirred at 70 °C for 18 h with DMF. The product was extracted, dried, concentrated, and separated by column chromatography to obtain Compound 8. Compound 8 was then reacted with biotin to create the final biotinylated berberine compound (compound 9).

### Biotin–avidin-mediated affinity purification

To study the binding of the UreB protein to EPI, an oligonucleotide affinity purification experiment was performed on the basis of a biotin–avidin affinity system. One milliliter of the magnetic bead suspension was pipetted into an EP tube, and the EP tube was placed on the magnetic stand for 15 minutes. The supernatants were discarded, and the beads were resuspended in lysis buffer. This process was repeated three times. Afterwards, 100 μL of biotin-conjugated EPI probes were mixed with 0.3 μg of the UreB-purified protein and incubated for 4 h at 4 °C with rotation. Fresh protein magnetic beads were added to the mixture, and the mixture was incubated for 3 additional hours at 4 °C. The supernatants were discarded, and the beads were resuspended in HEPES buffer (this process was repeated three times). The washed magnetic beads were added to the loading buffer, boiled at 95 °C for 10 minutes, and stored at –80 °C. The binding interactions were assessed using Coomassie brilliant blue staining and Western blot analysis.

### Construction of a phage mutation library

We constructed a synthetic phage‐displayed library consisting of 37 amino acid residues derived from the UreB gene. The sites selected for mutagenesis include those involved in nickel binding, substrate interaction, and pocket surface formation. The expected protein size for the full-length protein was 29 kDa. The protein size is similar to that of phage-displayed scFv selection antibodies, resulting in higher display efficiency. The green area is the saturation mutation site (Supplementary Table 3). The N-terminal sequence maintains the β-fold conformation, whereas the C-terminal sequence preserves the α-helix structure. The mutation site employs the NNK codon to incorporate bases, resulting in the potential expression of 20 amino acids, each with an equal probability of occurrence. The DNA sequence of the gene synthesis template is shown in Supplementary Table 3. The sequence includes a terminator TAG codon, which is suppressed during phage display and subsequently translated as the amino acid glutamine (Q). The yellow region denotes the enzyme cleavage site, whereas the red region indicates the location of the NNK synthesis mutation. The residual DNA sequences serve as template sequences and consist of natural nucleotides (Supplementary Table 3).

The DNA fragment for the linear library was synthesized by Sangon Bioengineering Co., Ltd. (Shanghai), and the necessary mutation primers are detailed in Supplementary Table 3. The gene fragments were combined with the digested PBC vector, ligated using DNA ligase, and incubated overnight at 16 °C. Fresh TG1 competent cells were prepared, to which the ligation product was added, mixed on ice, subjected to heat shock, and resuspended in 2YT medium. The resuspended products were plated on 2YT agar plates and incubated overnight at 37 °C. Colonies were selected and cultured in 2YT medium, followed by the addition of phage for infection and amplification to generate a peptide protein library. The resulting phage library was dissolved in PBS, and its titer was determined through gradient dilution. Next-generation sequencing (NGS) was performed by Sangon Bioengineering (Shanghai) Co., Ltd., Service.

### Binding of the EPI biotinylated probe to the UreB phage library

The phage library was introduced into the blocking solution at a final concentration of 1 × 10^12^ phages/mL and subjected to gentle agitation at 20 rpm for 1 hour at ambient temperature. Two 1.5 mL tubes were prepared by adding avidin-coated beads and blocking solution, followed by placement on a rotary mixer at 20 rpm for 1 hour at room temperature. The supernatant was carefully removed, and the beads were washed with PBS. Subsequently, 1 mL of the biotinylated probe and the phage library were added to separate tubes. The supernatant from the probe tubes was discarded, and the beads were washed with PBS again. The phage library was then added, mixed by inversion, and placed on a rotary mixer for 1 hour at 20 rpm at room temperature. The tubes were subsequently centrifuged at 800 × g for 10 min, after which the supernatant was removed. The beads were subsequently washed with 1 mL of PBST 5 times. Neutralization buffer was subsequently added, and the eluted phage was introduced into 20 mL of TG1 for infection, leading to amplification of the phage library. The amplified phage library was then collected for subsequent rounds of screening, which were conducted for a total of four cycles.

### Urease inhibitors combined with antibiotics in vitro

The overnight-activated ATCC 43504 strain was transferred and cultured in the logarithmic phase, and the bacterial concentration was adjusted to an OD600 of 0.1, corresponding to a concentration of 3 × 10^7^ CFU/mL. ATCC 43504 suspensions were treated with urease inhibitor (EPI and AHA) and antibiotic (tetracycline, clarithromycin, metronidazole and levofloxacin) mixtures (1/16× MIC, 1/8× MIC, 1/4× MIC, 1/2× MIC, 1× MIC, 2× MIC, and 4× MIC) for 24 h. For example, EPI + tetracycline (TET) (4× MIC) was prepared by mixing 4× MIC EPI with 4× MIC tetracycline. Urease inhibitors or antibiotics at a standard concentration of 1× MIC served as controls. The plates were incubated at 37 °C for 24 h under appropriate conditions. The OD600 was measured using a microplate reader to determine growth inhibition.

### Metagenomic analysis

Fresh fecal samples from mice were collected using the tail suspension method and promptly stored in liquid nitrogen. To prevent cross-contamination, the samples were sterilized and cleaned in a timely manner. Each sample was assigned a unique identifier, and relevant background information was documented. DNA extraction, library preparation, and metagenomic sequencing were conducted by Shanghai Meiji Biomedical Technology Co., Ltd. Species classification, and abundance information for each sample were obtained at various taxonomic levels (domain, kingdom, phylum, class, order, family, genus, and species) through comparison with the NR database. This was used as a basis for subsequent statistical analysis at the species level and further analysis of the samples using relevant dataset websites such as the Antibiotic Resistance Genes Database (ARDB). The DNA sequencing for comparison and the database were both provided by Shanghai Meiji Biomedical Technology Co., Ltd.

### Mutation analysis of bacterial genome resequencing

Genomic DNA was extracted from each individual, randomly fragmented, and fragments of the desired length were recovered by electrophoresis. Adapters were then added for cluster generation, followed by sequencing. We used fastp v0.20.0 to assess the sequencing quality and to preprocess the raw sequencing data. A 4-base pair sliding window was set to excise sequences with an average quality score below Q20 within the window. Sequences containing *N* bases and those shorter than 30 base pairs posttrimming were also removed. BWA software (http://biobwa.sourceforge.net/) was used to align the sequencing reads with the reference genome sequence using the MEM algorithm. Picard tools were used to remove PCR-duplicated reads. The sequencing depth and coverage relative to the reference genome were subsequently calculated on the basis of the BAM result files. GATK software was used to locally realign reads near indels to produce a realigned BAM file, thereby reducing false-positive SNPs near indels. Snippy 4.6.0 software was used to identify SNPs, small indels, and other relevant information, with low sequencing depth and alignment quality sites being filtered out. Finally, variant site annotation was performed using SnpEff (http://snpeff.sourceforge.net/SnpEff.html) to assess the impact of mutations on the genome.

### Statistical analysis

Statistical analysis was performed with GraphPad Prism version 8.0. Data are shown as means ± standard deviation (SD). Statistical comparisons were performed by one-way analysis of variance (ANOVA), and two sample student's t-tests. *P* < 0.05 was considered as statistically significant difference, and *P* < 0.01 was extremely significant difference. Statistical parameters and significance are reported in the figure legends. For comparing two sets of data, a two-tailed Student’s Ttest was performed. For comparing multiple data sets, a one-way ANOVA with multiple comparisons with Tukey post-hoc test was used for normal distributions, and a Mann-Whitney U test was used for non-normal distributions.Data are determined to be statistically significant when *p* < 0.05. Asterisks denote statistical significance as: *p < 0.05; **, *p* < 0.01; ***p < 0.001, compared to indicated controls. Error bars indicate standard deviation (SD). All other graphical representations are described in the Figure legends. Statistical analyzes were performed using GraphPad Prism version 2.0.

## Results

### Targeting H. pylori urease reduces the likelihood of drug resistance development

**Figure 1.**
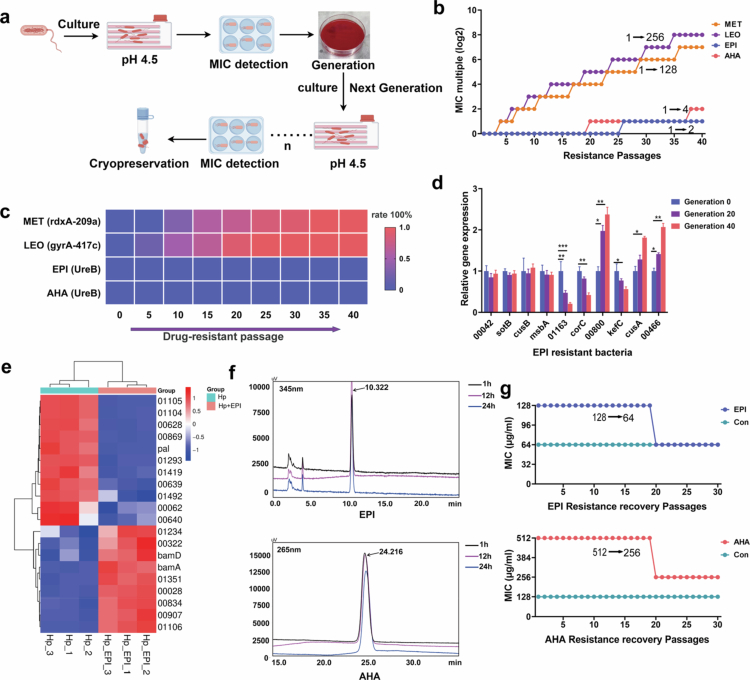
Targeting *H. pylori* urease is unlikely to induce drug resistance. (a) Schematic of drug resistance development in *H. pylori* under acidic conditions (pH 4.5). (b) Minimum inhibitory concentration (MIC) alterations occurred over 40 generations in a continuously drug-resistant culture under the acidic condition. (c) Analysis of the mutation rate in genes by Sanger sequencing. To detect mutations in the target site, primers for the target site in the *rdxA*, *gyrA*, and *UreB* genes were designed. (d) Quantitative real-time PCR (qPCR) analysis of multiple efflux pump genes across the 0^th^, 20^th^, and 40^th^ generations. Data are presented as mean ± SD. *, *p* < 0.05; **, *p* < 0.01; and ***, *p* < 0.001. (e) Heatmap and hierarchical clustering of high-throughput mRNA sequencing data of the HP and HP + EPI groups. (f) High-performance liquid chromatography (HPLC) was used to evaluate the degradation of EPI and AHA in *H. pylori* at 1 h, 12 h and 24 h. (g) MIC detection of drug-resistant bacteria under normal culture conditions. EPI: epiberberine; AHA: acetohydroxamic acid; Conn: control.

In *H. pylori*, urease is composed of *α* subunits (UreA) and β subunits (UreB), with one α subunit and one β subunit forming a heterodimeric functional unit and 12 such units forming a spherical ((αβ)_3_)_4_ macromolecule.^32^^,^^33^ The nickel-dependent catalytic center for urea hydrolysis is located within the β subunit. In an attempt to better comprehend the UreB functions, we aligned mature UreB genome sequences. We analyzed 140 known *H. pylori* UreB sequences and found that they have extremely low numbers of single-nucleotide polymorphisms. Particularly within a 10 Å radius of the catalytic pocket, the sequences are nearly identical (Supplementary Figure S1). Therefore, we speculate that *H. pylori* urease is structurally conserved and resistant to mutations. To verify this, we established an acidic culture (pH 4.5 and 5 mM urea) to enable long-term cultivation of *H. pylori* dependent on urease activity (Supplementary Figure S2a).^26^ We knocked out urease and found that *H. pylori* could survive in a neutral environment but could not survive under acidic conditions (Supplementary Figure S2b–e). We subsequently used urease inhibitors to induce drug resistance under acidic conditions (Figure 1a). Metronidazole and levofloxacin were used as controls in this study. Rapid increases in the minimum inhibitory concentration (MIC) values for both metronidazole and levofloxacin were consistently observed. In contrast, the MIC values for the urease inhibitors epiberberine (EPI) and AHA exhibited minimal changes, increasing only 2-fold and 4-fold, respectively, by the 40^th^ generation (Figure 1b). These results showed that the treatment of *Helicobacter pylori* with EPI and AHA reduced resistance under acidic conditions compared to metronidazole and levooxygen.

The primary mechanisms of bacterial drug resistance include alterations in target genes, enhanced activation of efflux pump systems, reduced membrane permeability, biofilm formation, and enzymatic inactivation. Among these mechanisms, mutations in target genes are irreversible once they are acquired.^4^^,^^34-36^ Therefore, we performed gene sequencing of bacteria treated with these drugs to identify gene mutations. Mutations in the target gene of metronidazole, rdxA, emerged as early as the 5^th^ generation, while levofloxacin-resistant mutations in gyrA began to emerge by the 10^th^ generation. In contrast, the EPI- and AHA-treated bacteria showed no detectable mutations in UreB until the 40^th^ generation (Figure 1c). These results indicate that targeting urease prevents the development of genetic drug resistance.

We further investigated the potential mechanisms underlying the development of resistance to urease inhibitors. The increases in the MIC values for both antibiotics and urease inhibitors did not affect bacterial growth capacity or infectivity (Supplementary Figure S2f, g). Transmission electron microscopy analysis revealed that the morphology of the urease inhibitor-resistant bacteria remained largely unchanged across the 0^th^, 20^th^, and 40^th^ generations (Supplementary Figure S2h). This suggests that bacterial resistance does not arise through alterations in the cell wall structure. We quantified the expression of 10 efflux pump-associated genes in *H. pylori* and observed significantly upregulated expression of genes related to multiple efflux pump systems (Figure 1d and Supplementary Figure S2i). Additionally, transcriptomic analysis of EPI-treated and AHA-treated bacteria revealed significant alterations in channel protein expression (Figure 1e and Supplementary Figure S2g). High-performance liquid chromatography (HPLC) revealed that AHA and EPI were stably present in the bacteria (Figure 1f). Therefore, the low level of resistance to urease inhibitors is likely attributed to nongenetic factors, such as alterations in the efflux system and membrane permeability, rather than genetic mutations.

We cultured drug-resistant *H. pylori* strains in drug-free medium to assess the reversibility of resistance. Fortieth-generation resistant strains were continuously passaged in drug-free medium for 30 generations. The results revealed that the MIC values for metronidazole and levofloxacin remained stable throughout the entire culture period (Supplementary Figure S2k), whereas susceptibility to both EPI and AHA gradually increased over time (Figure 1g). Taken together, these findings highlight the inherently low resistance potential of urease-targeted strategies in *H. pylori*.

### Alanine scanning reveals urease's functional constraints in gastric adaptation

**Figure 2.**
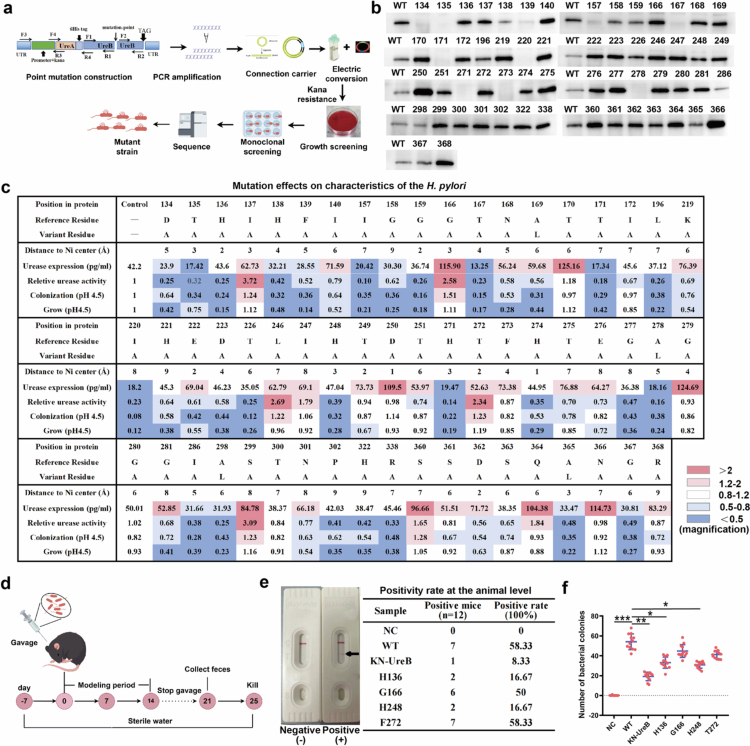
Alanine scan revealing key sites. (a) Alanine scanning roadmap, which provides an overview of the tool design and its main steps. (b) 6× His-tag protein expression levels were measured by Western blotting. (c) Effects of mutations on the characteristics of *H. pylori.* Distance between Ni atom and the specific residuals was calculated using Chimera version 1.16. Urease expression was measured by ELISA. Relative urease activity was measured by a urease activity assay kit. Colonization was confirmed by coculture of *H. pylori* and GES-1 cells under acidic conditions. Growth was assessed by constructing a growth curve under acidic conditions. (d) Schematic diagram of mouse infection with mutated *H. pylori*. (e) The positive infection rate for each group was measured using the *H. pylori* stool antigen (HPSA) assay. (f) The colonized bacteria was checked by counting the number of colony-forming units (CFUs) per gram tissue (*n* = 12).

To investigate why urease-targeted *H. pylori* eradication results in low resistance development, we utilized an alanine scanning mutagenesis strategy to dissect the structure‒function relationship of urease. This strategy capitalizes on the nonreactive methyl side chain of alanine while preserving protein secondary structure integrity.^37^ Through sequential substitution of individual amino acid residues with alanine or replacement of existing alanines with leucine, we were able to evaluate the functional consequences of specific mutations and identify essential catalytic sites (Figure 2a). We selected amino acid residues within a 10 Å radius of the nickel ions in the catalytic pocket for mutagenesis (Supplementary Figure S3a). All 58 mutant strains were successfully constructed, as verified by tagged protein detection and sequencing analysis (Supplementary Figure S3b, c). We first examined the effect of mutations on urease expression. Mutations induced divergent changes in urease expression levels, with both substantial decreases and increases observed following the introduction of mutations at specific residues (Figure 2b). These data were further supported by the ELISA results (Figure 2c and Supplementary Figure S3d). We subsequently evaluated changes in urease activity after amino acid residue mutations were introduced. We found that eight specific mutants (I137, G166, A246, I247, T272, S299, S360, and Q364) resulted in significantly increased urease activity. Moreover, ten mutations (T170, T249, D250, F273, G279, G280, T300, S361, N366, and R368) did not significantly alter enzymatic activity. Importantly, the remaining 40 residual mutations, representing 68.97% of the total, caused a marked decrease in urease activity (Figure 2c and Supplementary Figure S3e). These results indicate that most UreB point mutations result in impaired urease function.

Both the expression levels and activity of enzymes directly influence the acid tolerance of *H. pylori*, ultimately determining bacterial survival in acidic environments. To assess the impact of specific point mutations on the survival of *H. pylori*, we systematically analyzed the growth curves of each mutant strain under both neutral and acidic conditions. Our findings revealed that none of the individual mutations significantly altered bacterial growth under neutral pH conditions (Supplementary Figure S3f). Under acidic stress, compared with wild-type bacteria, no mutant strains exhibited superior adaptability, with 39 out of 58 strains showing significant growth inhibition (Figure 2c and Supplementary Figure S3g). These results may be attributed to the fact that the growth of organisms is governed by multiple synergistic factors, whereas the disruption of any single critical determinant can lead to death. In addition, while a subset of these mutations modestly enhanced colonization, the majority exhibited significantly impaired bacterial colonization (Figure 2c and Supplementary Figure S4a).

Furthermore, we developed a murine model of *H. pylori* infection to verify the impact of single mutations on bacterial colonization capacity in vivo (Figure 2d). We selected two strains characterized by increased urease activity (G166 and T272) and two strains characterized by reduced urease activity (H136 and H248). *H. pylori* infection in mice was assessed by three methods: an *H. pylori* IgG detection kit, a stool detection kit, and a mouse gastric tissue culture method. Compared with that of the standard ATCC 43504 strain, the infectivity of the G166 and T272 mutants was not changed, whereas the colonization capacity of the H136 and H248 strains was significantly attenuated (Figure 2e, f and Supplementary Figure S4b). Importantly, the compromised acid adaptation capacity directly correlated with reduced pathogenicity, as evidenced by diminished host inflammatory responses (Supplementary Figure S4c–f).

The impaired acid tolerance phenotype suggests that these mutants cannot effectively overcome gastric acid challenge, creating a survival disadvantage. This selection pressure likely explains the natural rarity of most urease mutations, as they compromise gastric colonization fitness. In this respect, mutations at the I137, G166, L246, I247, T272, S299, S360, and Q364 sites may persist, as these uniquely maintain or increase acid adaptation while avoiding fitness costs.

### Urease inhibitor binding sites avoid mutation hot spots

**Figure 3.**
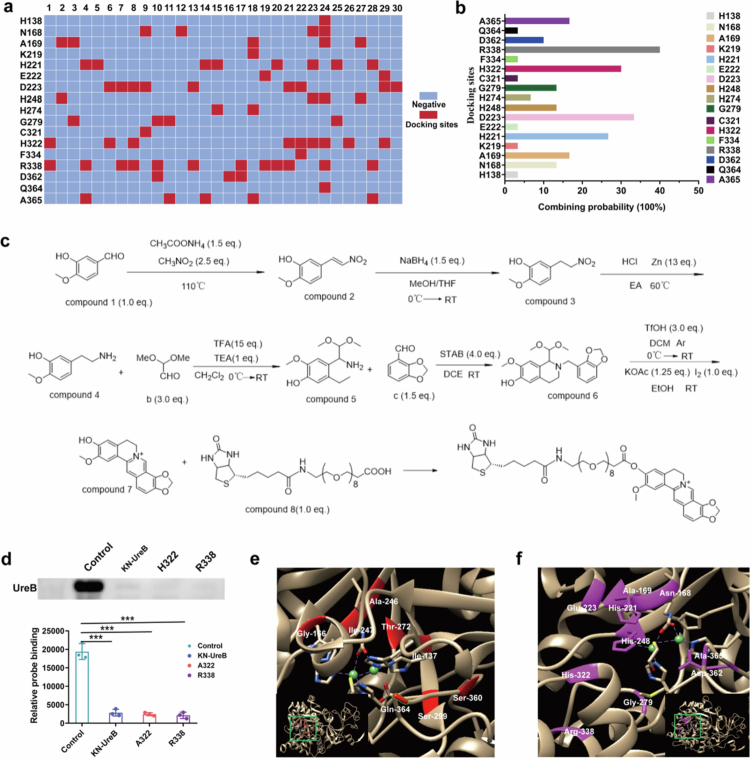
Urease inhibitor binding to urease. (a) Molecular docking analysis of 30 known urease inhibitors against urease. The binding sites are marked in red. (b) Frequency statistics of binding sites between these 30 inhibitors and urease. (c) Technical roadmap for biotin probe synthesis. (d) Validation of EPI binding sites on urease using biotin-modified EPI based on the biotin-avidin-mediated affinity purification system. ***, *p* < 0.001. (e) The mutation hot spots are visualized by using Chimera 1.16. Red represents easily mutating amino acids. (f) The visualized urease inhibitor binding sites. Pink indicates amino acids with high mutability.

To further elucidate why targeting urease is less likely to lead to the development of resistance, we analyzed the binding sites of 30 known urease inhibitors (Supplementary Figure S5), including EPI and AHA, and examined their binding site overlap with identified urease mutation hot spots (Figure 3a, b and Supplementary Figure S6a). These inhibitors function by competitively binding with urea or by blocking urea access to the hydrolysis site.^38^ To validate the accuracy of our analysis, we designed and synthesized a biotin-modified EPI probe that maintained antimicrobial activity equivalent to that of native EPI (Figure 3c and Supplementary Figure S6b, c). We successfully enriched the urease protein using this probe via the biotin-streptavidin system (Supplementary Figure S6d). We subsequently determined the binding sites of EPI to urease by assessing the binding affinity of the probe across mutant urease variants. Consistent with our prediction, mutations at residues H322 and R338, as well as UreB knockout, significantly diminished probe binding (Figure 3d).

Frequency analysis revealed that R338, D223, H322, and H221 were the most prevalent inhibitor binding sites, with frequencies of 40.0%, 33.3%, 30%, and 26.7%, respectively (Figure 3b). Crucially, our alanine scanning mutagenesis results demonstrated that these high-frequency sites are essential for catalytic activity (Figure 2c), indicating that most urease inhibitors function by blocking the catalytic activity of the enzyme. In addition, comparative analysis of urease inhibitor binding sites and identified mutation hot spots revealed that inhibitor binding sites exhibited near-complete spatial segregation from mutation hot spots. To explore the reasons for this, we analyzed the spatial distribution of mutation hot spots in urease. Unlike urease inhibitor binding sites, these mutation hot spots were buried within the protein interior close to the catalytic pocket rather than being exposed on the catalytic pocket surface (Figure 3e, f). Therefore, urease inhibitors could not directly interact with these mutation hot spots. Collectively, these results clarify the mechanism underlying the low likelihood of resistance when urease is targeted and provide a reference for the development of novel urease inhibitors.

### Deep mutational scanning of H. pylori urease using a phage display library

**Figure 4.**
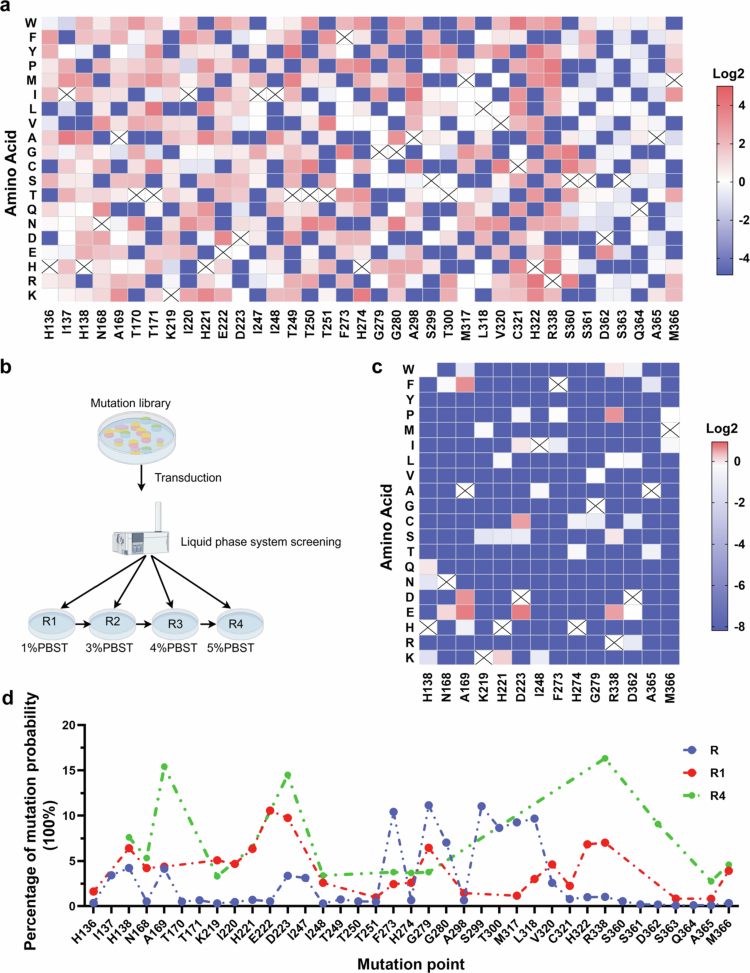
Phage library screening reveals the potential impact of a broad range of mutations. (a) Construction of the phage library. Mutations are marked with different colors based on their frequency. The amino acid identity of the parental strain at each site is marked with an “X”. (b) Schematic diagram of the binding of the biotin probe to the phage library. (c) The residual mutation library that exhibits binding to the probe was obtained after four rounds of screening. (d) Statistically analyzed mutation sites after four rounds of panning.

To comprehensively assess the impact of mutations on urease functionality and drug resistance, we thoroughly evaluated random mutations at sites potentially influencing urease activity by constructing a phage display library. The candidate sites for mutagenesis include Ni-binding sites, substrate binding sites, and pocket surface sites (Supplementary Figure S7a). Finally, 37 sites were chosen for library construction. We randomly selected a subset of clones for library validation (Supplementary Figure S7b). Seven mutation hotspots (F273, G279, G280, S299, T300, M317, and I318) with particularly high mutation frequencies under low-pH pressure were identified (Figure 4a). Notably, these mutations did not cause significant changes in the proliferation, colonization, or urease activity of *H. pylori* in acidic environments (Figure 2f). This suggests that bacteria cannot mutate to acquire better acid adaptability and at the same time do not produce mutations that diminish acid tolerance. These mutations are likely to decrease survival and lead to elimination under stronger or weaker acidic conditions.

To investigate how mutations affect inhibitor binding, we performed gradient elution of the mutant library using a biotinylated EPI probe.^39^ On the basis of binding strength, we categorized the mutants into four distinct pools (R1–R4; Figure 4b). Compared with the original 37-site library (R), 24 mutated residues bound to R1, whereas 13 mutation types failed to bind to EPI (Supplementary Figure S7c). Strikingly, 14 amino acid mutations in R4 resulted in exceptional binding affinity, with point mutations at A169, D223, and R338 resulting in the strongest interactions with EPI (Figure 4c, d). These sites are essential for the proliferation, colonization, and urease activity of *H. pylori* in acidic environments (Figure 2f). These findings explain the inherent ability of urease to prevent mutation-driven drug resistance.

### Urease inhibitors and antibiotics exhibit synergistic effects in combating H. pylori infection

**Figure 5.**
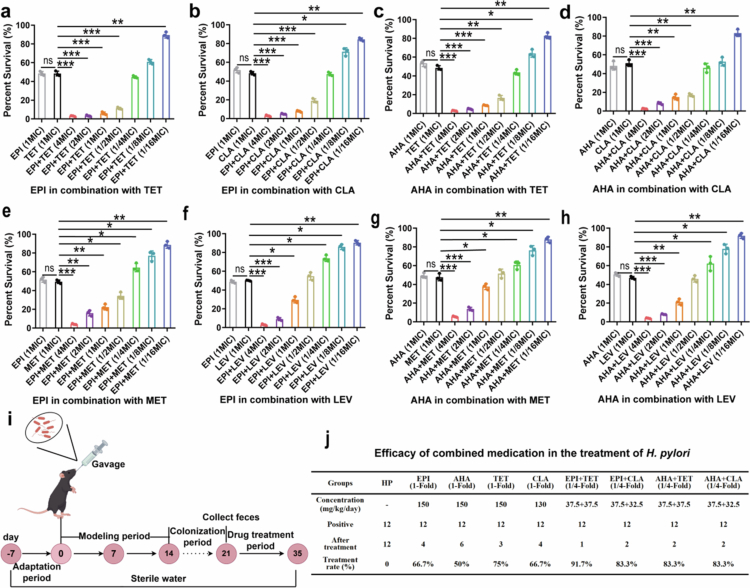
Combination of urease inhibitors and antibiotics in vitro and in vivo. (a, b) Combination of EPI and antibiotics (TET and CLA) in vitro. (c, d) Combination of AHA and antibiotics (TET and CLA) in vitro. (e, f) Combination of EPI and antibiotics (MET and LEV) in vitro. (g, h) Combination of AHA and antibiotics (MET and LEV) in vitro. MET: metronidazole; LEO: levofloxacin; EPI: epiberberine; AHA: acetohydroxamic acid. (i) Schematic diagram of the combined treatment against *H. pylori* infection in mice. (j) The synergistic effects of urease inhibitor and antibiotics.

Antibiotic resistance presents a major challenge in the treatment of *H. pylori* and is potentially linked to the misuse of antibiotics.^40^ Reducing antibiotic usage may alleviate this issue. Therefore, we investigated the potential synergistic effects of combining urease inhibitors with antibiotics to fight against *H. pylori* infection. In vitro, the combination of EPI or AHA with tetracycline or clarithromycin at 1/4× MIC exhibited an efficacy comparable to that of 1× MIC antibiotics alone (Figure 5a–d). While higher concentrations were needed for synergy with metronidazole or levofloxacin, enhanced activity was still evident (Figure 5e–h). These results indicate that urease inhibitors can be synergistically used with antibiotics.

Additionally, we investigated the in vivo synergistic effects of urease inhibitors combined with antibiotics (Figure 5i). Twelve *H. pylori*-infected mice were assigned to each group. Monotherapy with either urease inhibitors or antibiotics achieved *H. pylori* eradication rates ranging from 50% to 75% (Figure 5j). In contrast, combination therapy at 1/4× MIC increased the eradication rate to at least 83.3%. We conducted a preliminary safety assessment of urease inhibitors. Histological examination revealed no significant pathological alterations in mouse organs following treatment with the urease inhibitors EPI and AHA (Supplementary Figure S8a, b). Compared with monotherapy with either urease inhibitors or antibiotics alone, combination therapy not only enhanced therapeutic outcomes but also significantly reduced inflammation (Supplementary Figure S8c–e). Collectively, the combination of urease inhibitors with conventional antibiotics allows the standard therapeutic dose to be reduced, thereby indirectly mitigating antibiotic resistance.

### Combined treatment with urease inhibitors and antibiotics reduces the probability of resistance

**Figure 6.**
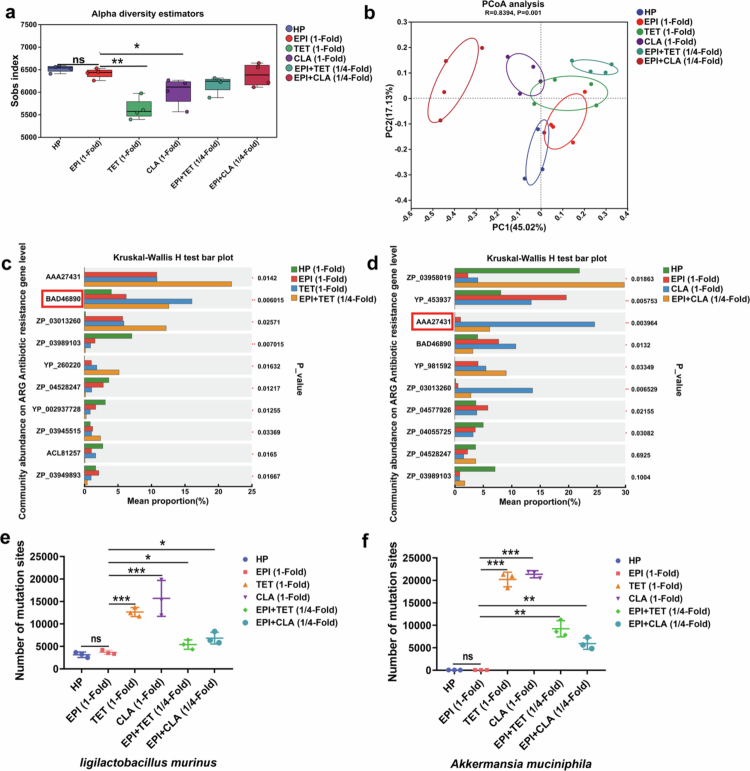
Macrogenomic analysis of drug-resistant mutations. (a) Comparative analysis of the difference in the *α* diversity index of the gut microbiota among different groups (Sobs diversity index). (b) PCoA of the gut microbiota among the different groups. PC1 and PC2 are two principal coordinate components; PC1 represents the principal coordinate component that explains the greatest possible change in the data, and PC2 is the principal coordinate component that explains the greatest proportion of the remaining degree of change. (c, d) Statistical analysis of ten major drug resistance genes identified by NGS data alignment against the ARDB database. (e) Number of mutation sites at the whole-genome level in *Lactobacillus murinus*. (f) Number of mutation sites at the whole-genome level in *Akkermansia muciniphila*. *, *p* < 0.05; **, *p* < 0.01; and ***, *p* < 0.001.

Currently, the majority of antibiotics employed in clinical settings for the treatment of *H. pylori* infections are broad-spectrum antibiotics.^4^^,^^41^ While they eradicate *H. pylori*, they also disrupt the homeostasis of the gut microbiota.^42^^,^^43^ To examine the impact of the combination of urease inhibitors with antibiotics on the gut microbiota, we performed metagenomic sequencing of fecal samples from the 6 treatment groups, obtaining a minimum of 42 million raw reads per sample with postassembly N50 values of 1,072–2,306 bp (Supplementary Figure S8f). We calculated the Shannon indices to estimate community diversity. An alpha diversity box plot revealed that antibiotic treatment significantly reduced microbial diversity, whereas urease inhibitors did not result in an obvious change (Figure 6a). Notably, combination therapy partially restored diversity. We conducted principal coordinate analysis (PCoA) using Bray‒Curtis dissimilarity and observed distinct clustering patterns across groups. The EPI and HP groups exhibited spatial proximity in the ordination plot, suggesting that urease inhibitors had a limited effect on the microbial community composition (Figure 6b).

Through a comparative analysis of 24 metagenome sequencing samples against the Antibiotic Resistance Genes Database (ARDB), we identified 205 distinct antibiotic resistance gene (ARGs). Functional analysis of the top 10 most abundant and differentially expressed genes revealed that in the EPI-tetracycline combination group, the *tetQ* gene (BAD46890),^44^ which protects ribosomes from translation inhibition by tetracycline, exhibited the most pronounced difference in expression (Figure 6c). Compared with urease inhibitors alone, tetracycline monotherapy significantly increased the expression of *tetQ*, while combination therapy partially reduced the expression of this resistance gene. Similarly, in the EPI-clarithromycin group, the *ermF* gene (AAA27431),^45^ which methylates adenine at position 2058 of 23S rRNA and thereby confers resistance to clarithromycin, exhibited the greatest difference in expression (Figure 6d). Clarithromycin treatment markedly elevated *ermF* levels, whereas combination therapy effectively decreased its expression.

To assess antibiotic-induced mutagenesis in the gut microbiota, we performed bacterial genome mutation analysis of the dominant species *Ligilactobacillus murinus* and *Akkermansia muciniphila*, which are beneficial commensals with high genomic abundance. The results demonstrated that EPI monotherapy induced no detectable genomic mutations, whereas tetracycline or clarithromycin monotherapy increased mutation frequencies by 10 to 100 times (Figure 6e, f). Importantly, compared with antibiotics alone, the combination of EPI and antibiotics significantly reduced mutation rates, indicating that coadministration of urease inhibitors with antibiotics can effectively suppress the development of antibiotic-induced resistance mutations in the gut microbiota.

## Discussion

The excessive use of antibiotics in the medical, veterinary, and animal husbandry fields represents a significant global issue, contributing to a marked increase in antibiotic resistance among numerous bacterial species.^46^ Eradication of *H. pylori* is among the primary reasons for clinical applications of antibiotics.^36^ However, the pervasive use of antibiotics has led to a continuous increase in antibiotic resistance.^47^ In this study, we unexpectedly discovered that *H. pylori* urease exhibits high structural conservation, and that inhibitors targeting urease decrease the development of resistance to antibiotics used to treat *H. pylori* infection. This arises from the critical dependence on urease activity for *H. pylori* survival in the uniquely acidic gastric environment, where mutations compromising urease function impose severe fitness costs not observed in other species. Therefore, urease-targeted *H. pylori* eradication may represent a promising therapeutic strategy that reduces the likelihood of drug resistance development.

Urease plays a crucial role in the pathogenesis of *H. pylori* infection through its enzymatic activity. By catalyzing urea hydrolysis in the gastric environment, urease generates ammonia, which forms a protective neutralization cloud around *H. pylori*, enabling gastric colonization and proliferation. In addition to its pH-regulating function, urease participates in the urea cycle and amino acid metabolism.^15^^,^^22^ While numerous studies have explored urease inhibition as an anti-*H. pylori* strategy,^28^^,^^48^ the precise active site architecture and critical functional domains remain incompletely characterized. In this study, we performed comprehensive alanine scanning mutagenesis of UreB, systematically analyzing 58 residues proximal to the nickel-containing active center. Our investigation revealed key amino acid sites governing *H. pylori* growth, colonization capacity, and urease activity. We found that most mutations substantially impaired urease function, concomitantly compromising bacterial fitness under acidic conditions. Furthermore, we generated a phage mutation library to emulate the random mutation of the UreB protein under natural conditions. Studies have shown that amino acids with a high probability of mutation generally do not significantly impact protein function, whereas crucial functional amino acid sites tend to be resistant to mutation.^49^^,^^50^ We mapped inhibitor binding sites on UreB and found that most coincided with regions essential for catalysis, indicating that inhibitors primarily act by blocking these critical functional domains. These findings suggest strong evolutionary constraints on urease mutagenesis, as most modifications would be deleterious to bacterial survival and explain why targeting urease results in reduced drug resistance development. Compared with previous reports on urease research,^14^^,^^28^ we have discovered that urease inhibitors are less prone to drug resistance against *H. pylori* under acidic conditions. Furthermore, we provide an in-depth analysis of the contribution of individual urease sites to *H. pylori*. This offers a novel strategy for the future design of urease-targeted drugs for treating *H. pylori* infection.

The global increase in resistance to antibiotics used to treat *H. pylori* infection has emerged as a primary contributor to treatment failure,^51^ necessitating innovative approaches to enhance drug delivery, optimize therapeutic outcomes, and minimize antibiotic doses.^52^ On the other hand, broad-spectrum antibiotics remain the mainstay for *H. pylori* eradication in contemporary clinical practice,^53^ yet their nonselective antimicrobial activity disrupts gut microbiota homeostasis^54-56^ and induces dysbiosis-linked inflammation and metabolic dysfunction.^56-58^ The exclusive presence of urease in select microorganisms such as *H. pylori* enables targeted pathogen eradication. In this study, we investigated the synergistic effects of urease inhibitors in conjunction with traditional antibiotics. Although we strove to make the model as close to the in vivo environment as possible during its construction, the inherent drawback of simplified conditions could not be entirely avoided. Our results demonstrate that urease inhibitor-antibiotic combinations not only potentiated antimicrobial efficacy but also significantly reduced the antibiotic dose needed to treat *H. pylori*-infected mice without compromising therapeutic effectiveness. These findings highlight a promising strategy to circumvent antibiotic resistance by integrating urease inhibitors into existing regimens, thereby reducing selective pressure while maintaining clinical efficacy. Although there are some differences between in vivo and in vitro experiments, we strive to ensure consistency in experimental conditions and guarantee the accuracy of the experimental results. For example, we maintain the same temperature, the same pH value, the same concentration of drugs, and so on. The strategy of co-administering conventional antibiotics with urease inhibitors represents a compelling therapeutic paradigm, as it combines direct bactericidal or bacteriostatic activity with targeted disruption of urease-dependent survival mechanisms.^59^ By attenuating urease activity, which is critical for environmental adaptation in certain pathogens, this approach can potentiate antibiotic efficacy and potentially impede the emergence of resistant phenotypes. However, its clinical translation remains constrained by an early developmental stage, a paucity of large-scale trials, and unresolved pharmacokinetic and safety profiles.^60^ Continued preclinical validation and well-designed clinical studies are required to establish its utility and scalability in managing urease-producing pathogens. Furthermore, understanding pathogen-specific dynamics and optimizing inhibitor selectivity will be crucial for broad application.

In summary, our study demonstrates that compared with conventional antibiotics, targeting *H. pylori* urease may represents a promising therapeutic strategy with reduced potential for resistance development. The structural conservation of urease and its essential role in bacterial survival create evolutionary constraints that limit mutations that are resistant to antibiotic treatment without compromising bacterial fitness. Combination therapy experiments demonstrate that urease inhibitors act synergistically with antibiotics, enabling a reduction in antibiotic dosage without compromising therapeutic effectiveness. These findings suggest that urease-targeted therapy could help mitigate the escalating challenge of antibiotic resistance in *H. pylori* infection treatment, providing a sustainable strategy for managing this widespread global health issue.

## Supplementary Material

supplemental information.docx supplemental information.docx

## Data Availability

All data are contained in the article and the supporting information or from the corresponding author upon reasonable request.
