# Supplementary material for: The functional and catalytic landscape of urease reveals a conserved target against Helicobacter pylori
Source: Gut Microbes. 2026 Apr 3;18(1):2653575. doi: 10.1080/19490976.2026.2653575 (PMC13051613; doi:10.1080/19490976.2026.2653575)
Supplement: supplemental information.docx [file KGMI_A_2653575_SM4233.docx]

Supplementary Figures

**
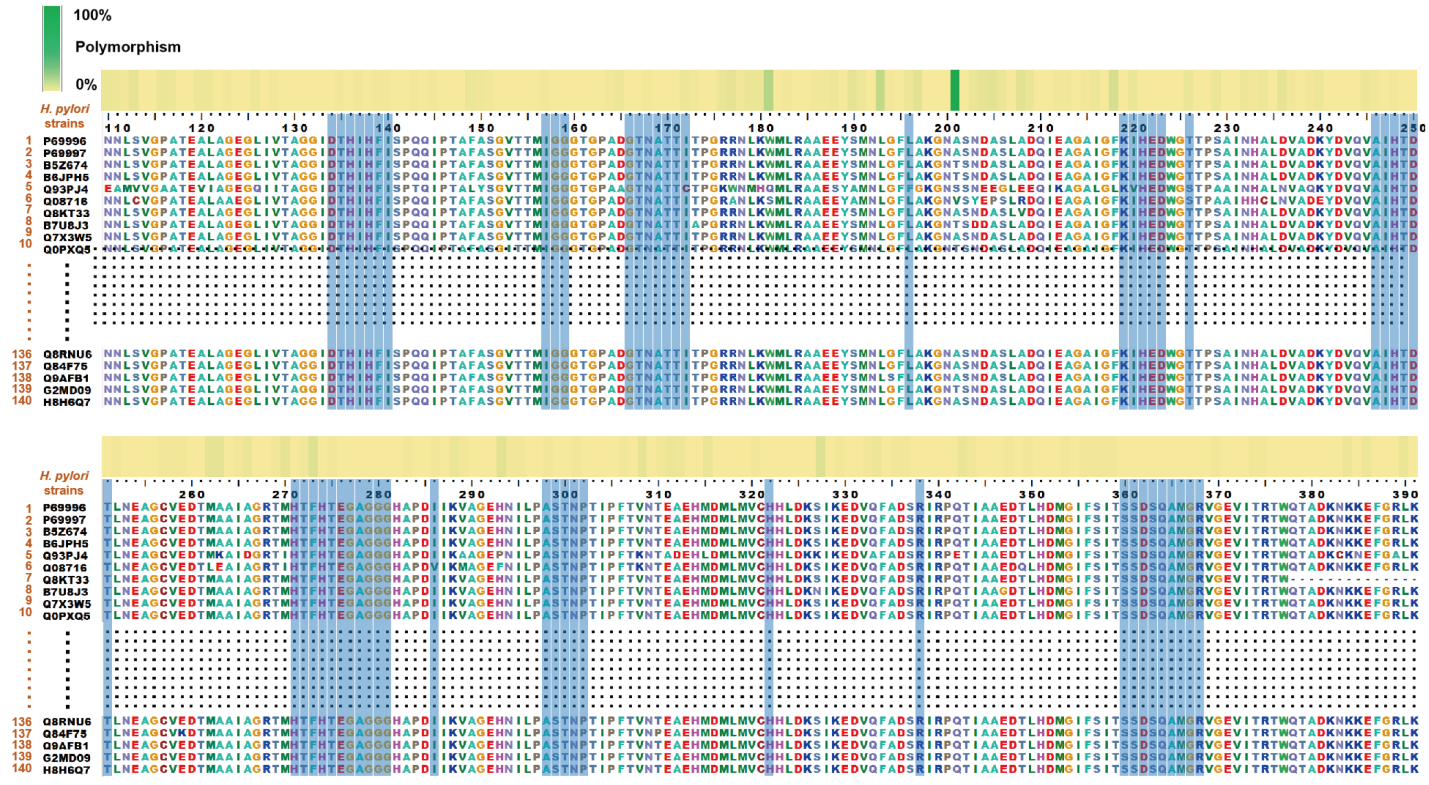
**

**Supplementary Figure S1. Results of multiple sequence alignment.** Evolutionary conservation of the UreB gene across a range of species. Protein alignment demonstrated high conservation of most amino acids among different species. The blue highlighted part represents the position near the Ni atom in the active site of urease.


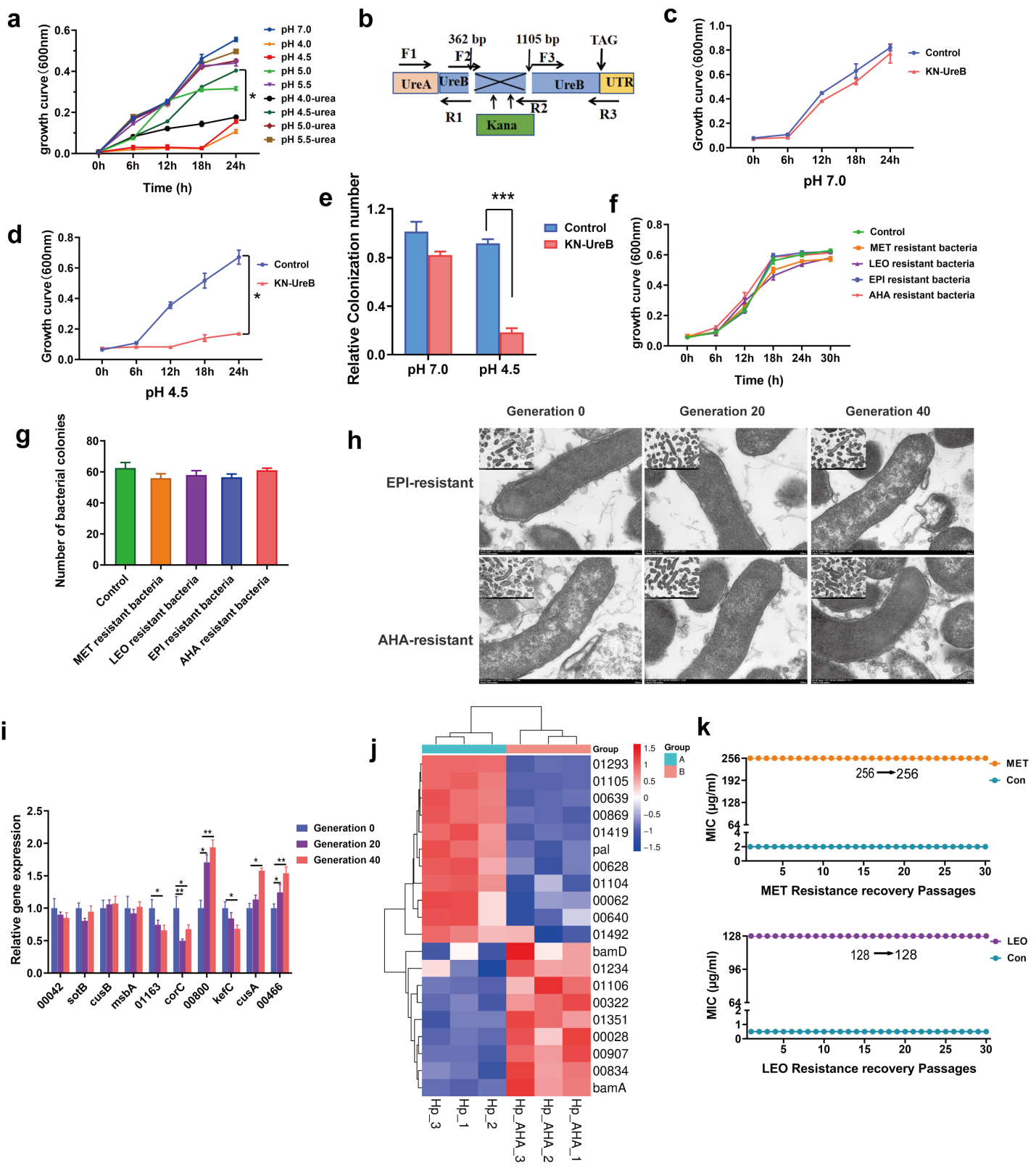


**Supplementary Figure S2. *UreB* knockout (KN-*UreB*) and resistance analysis. (a)** Growth curves of *H. pylori* in the presence of 5 mM urea under different pH conditions. The growth curve was plotted using the OD600 value at different times (0 h, 6 h, 12 h, 18 h, and 24 h). (**b)** Construction of the *UreB* knockout strain. (**c)** Growth curves of *UreB* knockout *H. pylori* at pH 7.0; the wild-type ATCC 43504 strain was used as a control. (**d)** Growth curves of *UreB* knockout *H. pylori* at pH 4.5; the wild-type ATCC 43504 strain was used as a control. (**e)** Comparison of the colonization of the WT and KN-*UreB* strains under different pH conditions (pH 7.0 and pH 4.5). The number of asterisks indicates the level of significance: ***, *p* < 0.001. (**f)** Growth curves of four drug-resistant bacteria. MET: metronidazole; LEO: levofloxacin; EPI: epiberberine; AHA: acetohydroxamic acid. The wild type ATCC 43504 strain was used as a control. (**g)** Comparison of the colonization of the four drug-resistant bacteria at pH 4.5. The wild-type ATCC 43504 strain was used as a control. (**h)** Transmission electron microscopy analysis of the morphology of the EPI- and AHA-resistant bacteria across the 0th, 20th, and 40th generations. Magnification: × 30,000; scale bar: 500 nm; × 8,000; scale bar: 2.0 μm. (**I)** Real-time quantitative PCR (qRT‒PCR) analysis of the expression levels of AHA resistance-related genes in the 0^th^, 20^th^, and 40^th^ generations. The bars represent the means ± SDs of three samples. The grey column indicates the qRT‒PCR value when 16S rRNA was used as a reference gene. The number of asterisks represents the level of significance: *, *p* < 0.05; **, *p* < 0.01; and ***, *p* < 0.001. (**j)** Heatmap and hierarchical clustering of high-throughput miRNA sequencing data. Hierarchical clustering heatmaps of the mRNA expression data of the HP and HP+AHA groups. The red line indicates high relative expression, and the blue line indicates low relative expression. HP group: *H. pylori* group; HP+AHA group: *H. pylori* and AHA treatment group. **(k)** MIC detection of drug-resistant bacteria under normal culture conditions. MET: metronidazole; LEO: levofloxacin; Con: control.


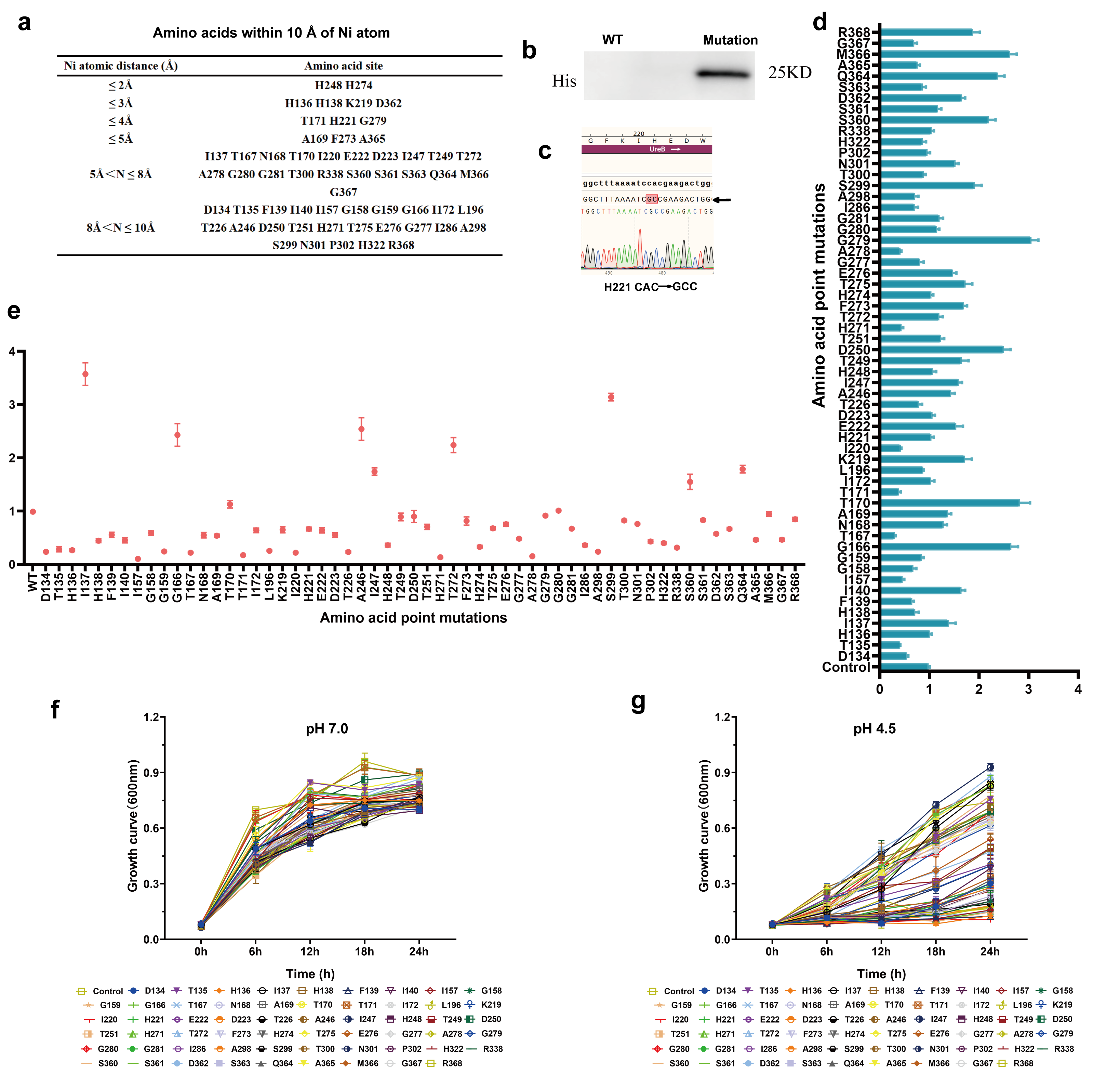


**Supplementary Figure S3.** **Changes in various mutation sites in *H. pylori* after alanine scanning mutation. (a)** Amino acid statistics within a range of 10 Å from the Ni atom active centre using Chimera version 1.16. (**b)** Western blotting was used to determine whether the 6 × His-tag was successfully inserted into the *UreB* gene. (**c)** Mutation of the amino acid site of *H. pylori* was successfully confirmed using Sanger sequencing. (**d)** Urease expression after point mutation introduction determined by ELISA. (**e)** Urease activity after point mutation introduction determined by a urease activity assay kit. (**f, g)** Growth curves of 58 types of bacteria with point mutants at pH 7.0 and pH 4.5; the wild-type ATCC 43504 strain was used as a control.


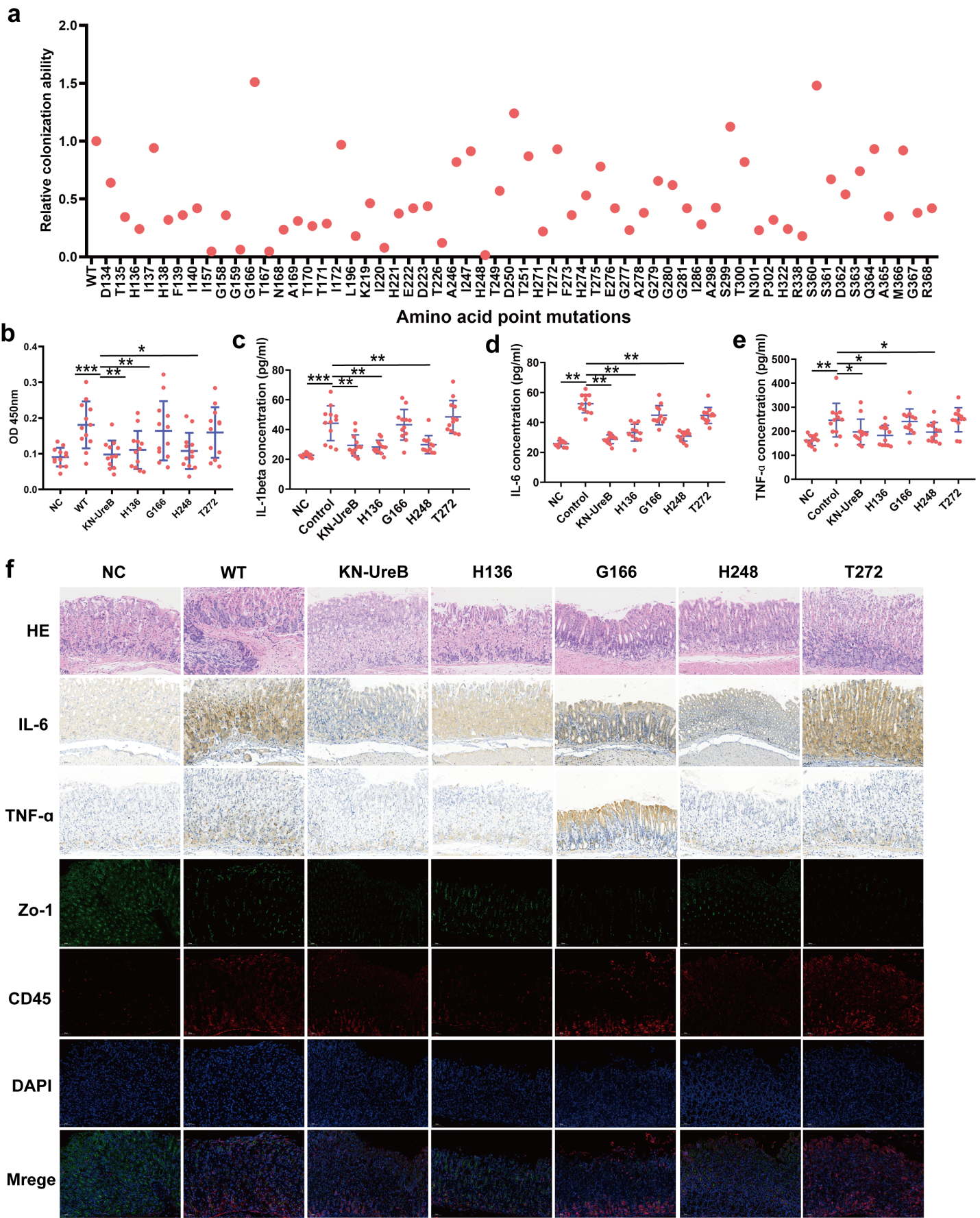


**Supplementary Figure S4. ELISA and histological analysis of inflammatory markers. (a)** Colonization was evaluated through coculture of *H. pylori* and GES-1 cells under acidic conditions. The normal ATCC 43504 strain was used as a control. Each experiment was performed in triplicate. (b**)** *H. pylori* IgG levels in the serum were measured by ELISA. (**c, d, e)** The concentrations of IL-1 beta, IL-6 and TNF-ɑ in the serum of mice were determined by ELISA. Each group contained 12 samples. The number of asterisks represents the level of significance: *, *p* < 0.05; **, *p* < 0.01; ***, *p* < 0.001. **(f)** Effect of mutant bacteria on stomach histopathological changes determined by HE staining. Immunohistochemical analysis of the expression of the gastric inflammatory mediators IL-6 and TNF-ɑ. The expression levels of CD45 and ZO-1 in the stomach slices were examined using immunofluorescence analysis. Green fluorescence represents ZO-1, red fluorescence represents CD45, and blue fluorescence represents the cell nucleus. Magnification: × 200; scale bar: 50 µm.

**
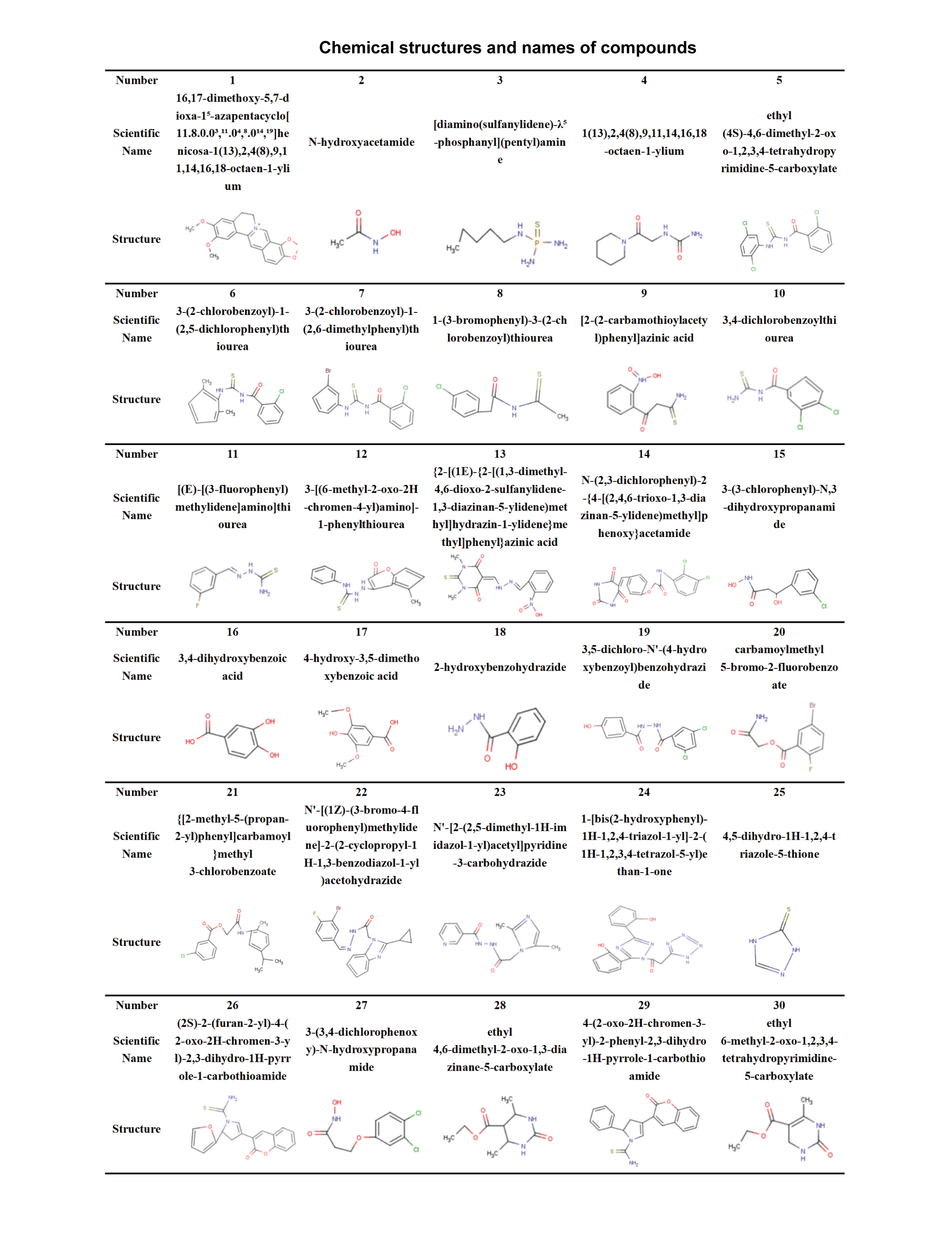
**

**Supplementary Figure S5. Names and structures of 30 urease inhibitors.**


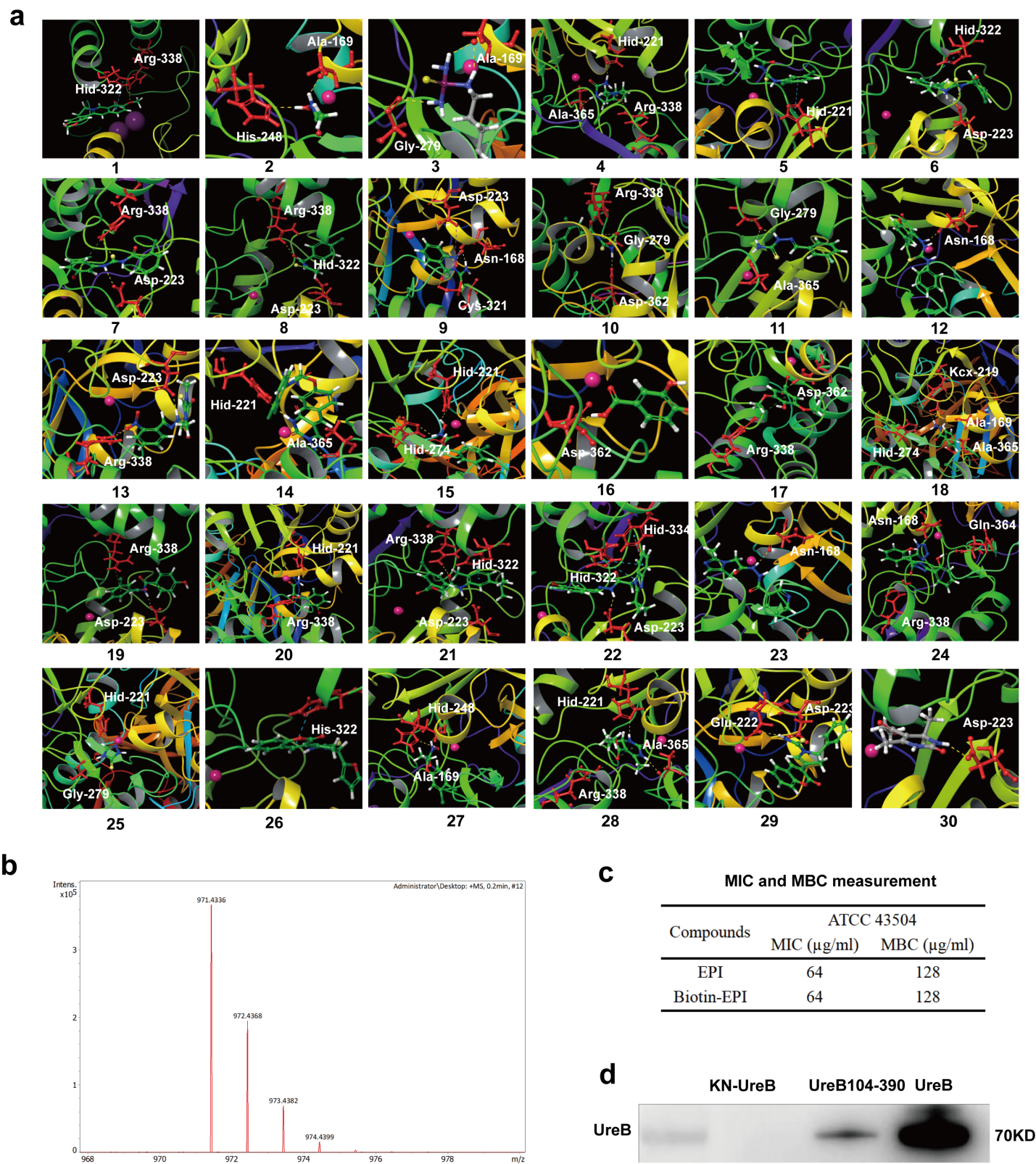


**Supplementary Figure S6. Molecular docking analysis of urease inhibitors and urease. (a)** Molecular models of 30 known urease inhibitor compounds binding to urease. Red represents conjugated amino acids, and green and white represent urease inhibitors. (**b)** The molecular weight of the EPI biotin probe was determined by mass spectrometry (MS). (**c)** MIC and MBC values of EPI and biotin-EPI against *H. pylori*. (**d)** Bound proteins were detected using the UreB protein. The binding ability of the EPI biotin probe was detected using the UreB protein. KN-UreB: UreB knockout strain; UreB104-390: a truncated form of the protein that encodes amino acids 104-390 of UreB; UreB: full-length UreB protein.


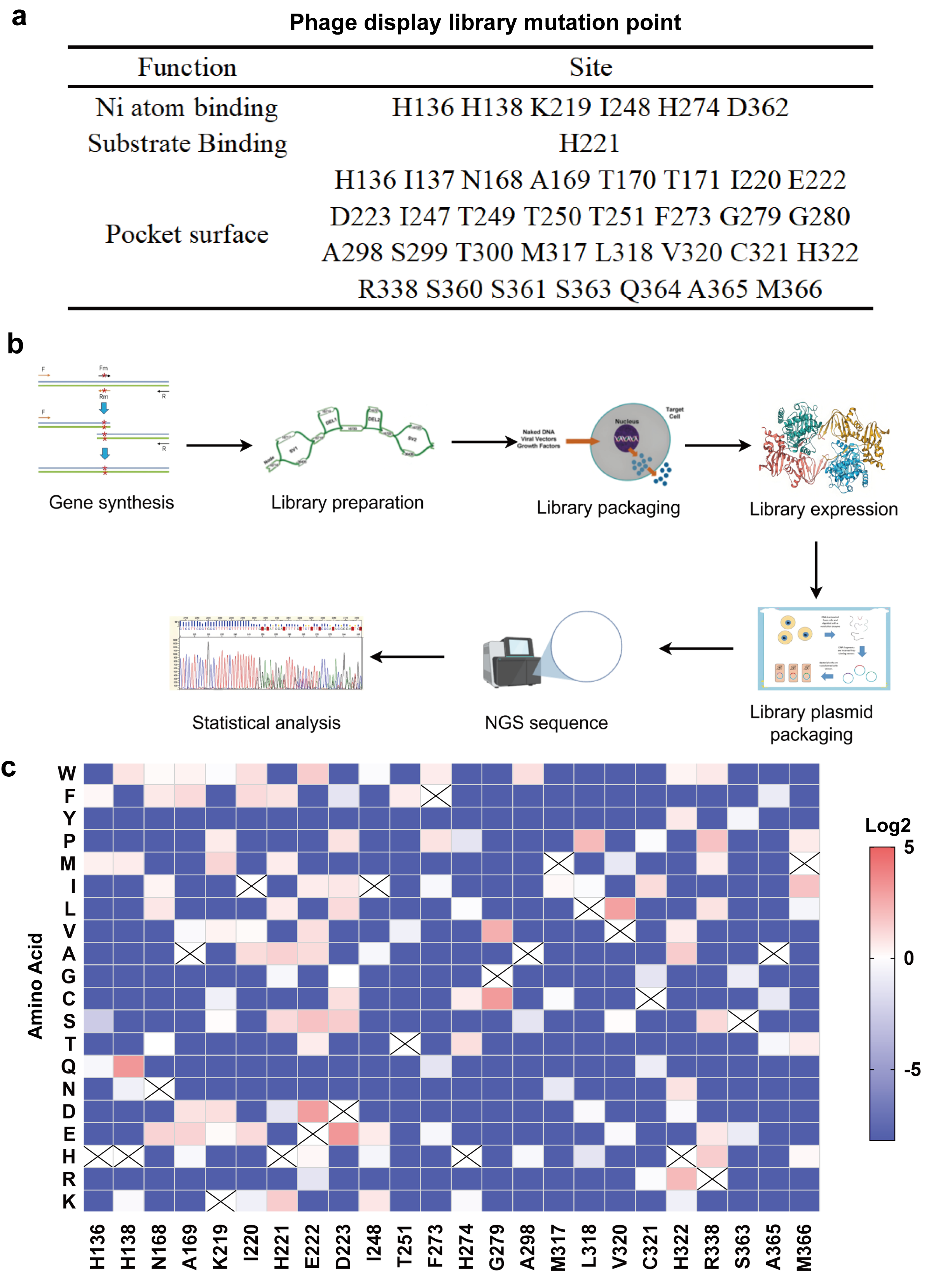


**Supplementary Figure S7. Construction and screening of the phage library. (a)** The candidate sites for mutagenesis include Ni-binding sites, substrate binding sites, and pocket surface sites. (**b)** Flowchart for constructing the phage library. (**c)** First combination of a phage mutation library and a biological probe for screening the remaining mutations. The biotin probe initially interacted with the phage display library, and the residual amino acid mutation library was subjected to screening. High-frequency mutations are coloured red, whereas low-frequency mutations are coloured dark blue. White squares are mutations that were missing. The amino acid identity in the parental strain at each site is represented with an “X”. The number of times each mutation occurred was processed through log2 transformation.


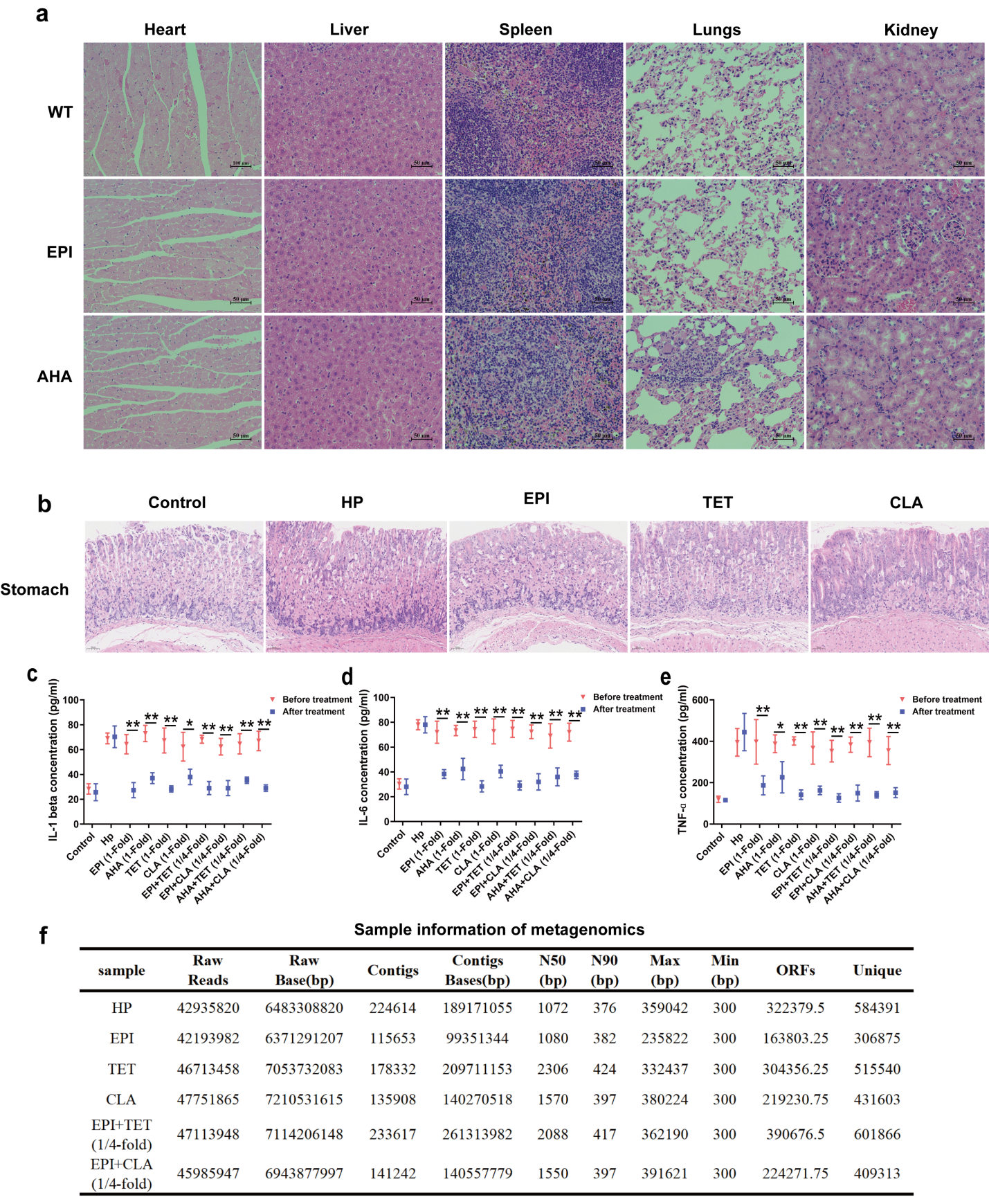


**Supplementary** **Figure S8. HE staining and sample information for metagenomics. (a)** HE staining to assess the morphological changes in the heart, liver, spleen, lungs, and kidneys of mice after treatment with EPI or AHA. Magnification: × 200; scale bar: 50 µm. (**b)** HE staining to assess the morphological changes in the stomachs of mice after treatment with EPI, AHA, TET or CLA. Magnification: × 200; scale bar: 50 µm. (**c, d, e)** The concentrations of IL-1 beta, IL-6, and TNF-ɑ in the serum of the mice were determined by ELISA. Each group contained 12 samples. The number of asterisks indicates the level of significance: *, *p* < 0.05; **, *p* < 0.01; and ***, *p* < 0.001. (**f)** Sample information for metagenomics. Raw reads: number of sequence entries in raw reads; Raw bases: number of bases in raw reads; Contigs: number of sequence entries in contigs; Contigs bases: total sequence length of contigs; N50 (N90): sort the contigs sequences in order of length, and add up the length values of the scanned sequences one by one from the largest to the smallest, when the cumulative value exceeds 50% (90%) of the total length of all the sequences, then the scanned sequences are summed up. Compared with the average length of the contigs sequences, the N50 (N90) is a more accurate representation of the effect of the contigs splicing; Max: the length of the contigs with the longest lengths. Min: length of the shortest contig; ORFs: number of ORFs. Four biological replicates in each experimental group.

Supplementary Tables

Table S1.UreB amino acid sequence.

| **Number** | **ID number** | **Amino acid sequence** | **Length**  **(aa)** |
| --- | --- | --- | --- |
| 1 | P69996 | MKKISRKEYVSMYGPTTGDKVRLGDTDLIAEVEHDYTIYGEELKFGGGKTLREGMSQSNNPSKEELDLIITNALIVDYTGIYKADIGIKDGKIAGIGKGGNKDMQDGVKNNLSVGPATEALAGEGLIVTAGGIDTHIHFISPQQIPTAFASGVTTMIGGGTGPADGTNATTITPGRRNLKWMLRAAEEYSMNLGFLAKGNASNDASLADQIEAGAIGFKIHEDWGTTPSAINHALDVADKYDVQVAIHTDTLNEAGCVEDTMAAIAGRTMHTFHTEGAGGGHAPDIIKVAGEHNILPASTNPTIPFTVNTEAEHMDMLMVCHHLDKSIKEDVQFADSRIRaPQTIAAEDTLHDMGIFSITSSDSQAMGRVGEVITRTWQTADKNKKEFGRLKEEKGDNDNFRIKRYLSKYTINPAIAHGISEYVGSVEVGKVADLVLWSPAFFGVKPNMIIKGGFIALSQMGDANASIPTPQPVYYREMFAHHGKAKYDANITFVSQAAYDKGIKEELGLERQVLPVKNCRNITKKDMQFNDTTAHIEVNPETYHVFVDGKEVTSKPANKVSLAQLFSIF | 569 |
| 2 | P69997 | MKKISRKEYVSMYGPTTGDKVRLGDTDLIAEVEHDYTIYGEELKFGGGKTLREGMSQSNNPSKEELDLIITNALIVDYTGIYKADIGIKDGKIAGIGKGGNKDMQDGVKNNLSVGPATEALAGEGLIVTAGGIDTHIHFISPQQIPTAFASGVTTMIGGGTGPADGTNATTITPGRRNLKWMLRAAEEYSMNLGFLAKGNASNDASLADQIEAGAIGFKIHEDWGTTPSAINHALDVADKYDVQVAIHTDTLNEAGCVEDTMAAIAGRTMHTFHTEGAGGGHAPDIIKVAGEHNILPASTNPTIPFTVNTEAEHMDMLMVCHHLDKSIKEDVQFADSRIRPQTIAAEDTLHDMGIFSITSSDSQAMGRVGEVITRTWQTADKNKKEFGRLKEEKGDNDNFRIKRYLSKYTINPAIAHGISEYVGSVEVGKVADLVLWSPAFFGVKPNMIIKGGFIALSQMGDANASIPTPQPVYYREMFAHHGKAKYDANITFVSQAAYDKGIKEELGLERQVLPVKNCRNITKKDMQFNDTTAHIEVNPETYHVFVDGKEVTSKPANKVSLAQLFSIF | 569 |
| 3 | B5Z674 | MKKISRKEYVSMYGPTTGDKVRLGDTDLIAEVEHDYTIYGEELKFGGGKTLREGMSQSNNPSKEELDLIITNALIVDYTGIYKADIGIKDGKIAGIGKGGNKDMQDGVKNNLSVGPATEALAGEGLIVTAGGIDTHIHFISPQQIPTAFASGVTTMIGGGTGPADGTNATTITPGRRNLKWMLRAAEEYSMNLGFLAKGNTSNDASLADQIEAGAIGFKIHEDWGTTPSAINHALDVADKYDVQVAIHTDTLNEAGCVEDTMAAIAGRTMHTFHTEGAGGGHAPDIIKVAGEHNILPASTNPTIPFTVNTEAEHMDMLMVCHHLDKSIKEDVQFADSRIRPQTIAAEDTLHDMGIFSITSSDSQAMGRVGEVITRTWQTADKNKKEFGRLKEEKGDNDNFRIKRYLSKYTINPAIAHGISEYVGSVEVGKVADLVLWSPAFFGVKPNMIIKGGFIALSQMGDANASIPTPQPVYYREMFAHHGKAKYDANITFVSQAAYDKGIKEELGLERQVLPVKNCRNITKKDMQFNDTTAHIEVNPETYHVFVDGKEVTSKPANKVSLAQLFSIF | 569 |
| 4 | B6JPH5 | MKKISRKEYVSMYGPTTGDKVRLGDTDLIAEVEHDYTIYGEELKFGGGKTLREGMSQSNNPSKEELDLIITNALIVDYTGIYKADIGIKDGKIAGIGKGGNKDMQDGVKNNLSVGPATEALAGEGLIVTAGGIDTHIHFISPQQIPTAFASGVTTMIGGGTGPADGTNATTITPGRRNLKWMLRAAEEYSMNLGFLAKGNTSNDASLADQIEAGAIGFKIHEDWGTTPSAINHALDVADKYDVQVAIHTDTLNEAGCVEDTMAAIAGRTMHTFHTEGAGGGHAPDIIKVAGEHNILPASTNPTIPFTVNTEAEHMDMLMVCHHLDKSIKEDVQFADSRIRPQTIAAEDTLHDMGIFSITSSDSQAMGRVGEVITRTWQTADKNKKEFGRLKEEKGDNDNFRIKRYLSKYTINPAIAHGISEYVGSVEVGKVADLVLWSPAFFGVKPNMIIKGGFIALSQMGDANASIPTPQPVYYREMFAHHGKAKYDANITFVSQAAYDKGIKEELGLERQVLPVKNCRNITKKDMQFNDTTAHIEVNPETYHVFVDGKEVTSKPANKVSLAQLFSIF | 569 |
| 5 | Q09068 | MLGLVLLYVGIVLISNGICGLTKVDPKSTAVMNFFVGGLSIICNIVVITYSALHPTAPVEGAEDIAQVSHHLTSFYGPATGLLFGFTYLYAAINHTFGLDWRPYSWYSLFVAINTIPAAILSHYSDMLDDHKVLGITEGDWWAIIWLAWGVLWLTAFIENILKIPLGKFTPWLAIIEGILTAWIPAWLLFIQHWV | 195 |
| 6 | Q93PJ4 | MIKISRKQYASMYGPTTGDKVRLGDTNLFAEIEKDYTLYGEEIKFGGGKTIRDGMAQSASTYTNELDAVITNAMIIDYTGIYKADIGIKGGKIVGIGKAGNPDTQDSVNEAMVVGAATEVIAGEGQIITAGGIDTHIHFISPTQIPTALYSGVTTMIGGGTGPAAGTNATTCTPGKWNMHQMLRAAESYAMNLGFFGKGNSSNEEGLEEQIKAGALGLKVHEDWGSTPAAINHALNVAQKYDVQVAIHTDTLNEAGCVEDTMKAIDGRTIHTFHTEGAGGGHAPDIIKAAGEPNILPASTNPTIPFTKNTADEHLDMLMVCHHLDKKIKEDVAFADSRIRPETIAAEDTLHDMGIFSITSSDSQAMGRVGEVITRTWQTADKCKNEFGALKEECGENDNFRIKRYISKYTINPAIAHGISEYVGSVEVGKFADLVLWKPSMFGIKPEMILKNGMIVAAKIGDSNASIPTPEPVVYAPMFG  SYGKAKYNCAITFVSKIAYDCHIKEELGLERILLPVKNCRNITKKDMKFNDVITPIEVNPETYEVRVNNTKITSKPVEKVSLGQLYCLF | 569 |
| 7 | Q08716 | MKKISRKEYVSMYGPTTGDRVRLGDTDLILEVEHDCTTYGEEIKFGGGKTIRDGMSQTNSPSSYELDLVLTNALIVDYTGIYKADIGIKDGKIAGIGKAGNKDMQDGVDNNLCVGPATEALAAEGLIVTAGGIDTHIHFISPQQIPTAFASGVTTMIGGGTGPADGTNATTITPGRANLKSMLRAAEEYAMNLGFLAKGNVSYEPSLRDQIEAGAIGFKIHEDWGSTPAAIHHCLNVADE  YDVQVAIHTDTLNEAGCVEDTLEAIAGRTIHTFHTEGAGGGHAPDVIKMAGEFNILPASTNPTIPFTKNTEAEHMDMLMVCHHLDKSIKEDVQFADSRIRPQTIAAEDQLHDMGIFSITSSDSQAMGRVGEVITRTWQTADKNKKEFGRLKEEKGDNDNFRIKRYISKYTINPAIAHGISDYVGSVEVGKYADLVLWSPAFFGIKPNMIIKGGFIALSQMGDANASIPTPQPVYYREMFG  HHGKNKFDTNITFVSQAAYKAGIKEELGLDRVVLPVKNCRNITKKDLKFNDVTAHIDVNPETYKVKVDGKEVTSKAADELSLAQLYNLF | 569 |
| 8 | Q8KT33 | MKKISRKEYVSMYGPTTGDKVRLGDTDLIAEVEHDYTIYGEELKFGGGKTLREGMSQSNNPSKEELDLIITNALIVDYTGIYKADIGIKDGKIAGIGKGGNKDMQDGVKNNLSVGPATEALAGEGLIVTAGGIDTHIHFISPQQIPTAFASGVTTMIGGGTGPADGTNATTITPGRRNLKWMLRAAEEYSMNLGFLAKGNASNDASLVDQIEAGAIGFKIHEDWGTTPSAINHALDVADKYDVQVAIHTDTLNEAGCVEDTMAAIAGRTMHTFHTEGAGGGHAPDIIKVAGEHNILPASTNPTIPFTVNTEAEHMDMLMVCHHLDKSIKEDVQFADSRIRPQTIAAEDTLHDMGIFSITSSDSQAMGRVGEVITRTW | 377 |
| 9 | B7U8J3 | MKKISRKEYVSMYGPTTGDKVRLGDTDLIAEVEHDYTIYGEELKFGGGKTLREGMSQSNNPSKEELDLIITNALIVDYTGIYKADIGIKDGKIAGIGKGGNKDMQDGVKNNLSVGPATEALAGEGLIVTAGGIDTHIHFISPQQIPTAFASGVTTMIGGGTGPADGTNATTIAPGRRNLKWMLRAAEEYSMNLGFLAKGNTSDDASLADQIEAGAIGFKIHEDWGTTPSAINHALDVADKYDVQVAIHTDTLNEAGCVEDTMAAIAGRTMHTFHTEGAGGGHAPDIIKVAGEHNILPASTNPTIPFTVNTEAEHMDMLMVCHHLDKNIKEDVQFADSRIRPQTIAAGDTLHDMGIFSITSSDSQAMGRVGEVITRTWQTADKNKKEFGRLKEEKGDNDNFRIKRYLSKYTINPAIAHGISEYVGSVEVGKVADLVLWSPAFFGVKPNMIIKGGFIALSQMGDANASIPTPQPVYYREMFAHHGKAKYDANITFVSQAAYDKGIKEELGLERQVLPVKNCRNVTKKDMQFNDTTAHIEVNPETYHVFVDGKEVTSKPATKVSSAQLFSIF | 569 |
| 10 | Q7X3W5 | MKKISRKEYVSMYGPTTGDKVRLGDTDLIAEVEHDYTIYGEELKFGGGKTLREGMSQSNNPSKEELDLIITNALIVDYTGIYKADIGIKDGKIAGIGKGGNKDMQDGVKNNLSVGPATEALAGEGLIVTAGGIDTHIHFISPQQIPTAFASGVTTMIGGGTGPADGTNATTITPGRRNLKWMLRAAEEYSMNLGFLAKGNASNDASLADQIEAGAIGFKIHEDWGTTPSAINHALDVADKYDVQVAIHTDTLNEAGCVEDTMAAIAGRTMHTFHTEGAGGGHAPDIIKVAGEHNILPASTNPTIPFTVNTEAEHMDMLMVCHHLDKSIKEDVQFADSRIRPQTIAAEDTLHDMGIFSITSSDSQAMGRVGEVITRTWQTADKNKKEFGRLKEEKGDNDNFRIKRYLSKYTINPAIAHGISEYVGSVEVGKVADLVLWSPAFFGVKPNMIIKGGFIALSQMGDANASIPTPQPVYYREMFAHHGKAKYDANITFVSKAAYDKGIKEELGLERQVLPVKNCRNITKKDMQFNDTTAHIEVNPETYHVFVDGKEVTSKPATKVSLAQLFSIF | 569 |
| 11 | Q64EY3 | MISRKEYVSMYGPTTGDKVRLGDTDLIAEVEHDYTIYGEELKFGGGKTLREGMSQSNNPSKEELDLIITNALIVDYTGIYKADIGIKDGKIAGIGKGGNKDMQDGVKNNLSVGPATEALAGEGLIVTAGGIDTHIHFISPQQIPTAFASGVTTMIGGGTGPADGTNATTITPGRRNLKWMLRAAEEYSMNLGFLAKGNTSNDASLADQIEAGAIGFKIHEDWGTTPSAINHALDVADKYDVQVAIHTDTLNEAGCVEDTMAAIAGRTMHTFHTEGAGGGHAPDIIKVAGEHNILPASTNPTIPFTVNTEAEHMDMLMVCHHLDKSIKEDVQFADSRIRPQTIAAEDTLHDMGIFSITSSDSQAMGRVGEVITRTWQTADKNKKEFGRLKEEKGDNDNFRIKRYLSKYTINPAIAHGISEYVGSVEVGKVADLVLWSPAFFGVKPNMIIKGGFIALSQMGDANASIPTPQPVYYREMFAHHGKAKYDANITFVSQAAYDKGIKEELGLERQVLPVKNCRNITKKDMQFNDTTAHIEVNPETYHVFVDGKEVTSKPATKVSLAQLFSIF | 567 |
| 12 | Q0PXQ5 | MKKISRKEYVSMYGPTTGDKVRLGDTDLIAEVEHDYTIYGEELKFGGGKTLREGMSQSNNPSKEELDLIITNALIVDYTGIYKADIGIKDGKIAGIGKGGNKDMQDGVKNNLSVGPATEALAGEGLIVTAGGIDTHIHFISPQQIPTAFASGITTMIGGGTGPADGTNATTITPGRRNLKWMLRAAEEYSMNLGFLAKGNTSNDASLADQIEAGAIGFKIHEDWGTTPSAINHALDVADKYDVQVAIHTDTLNEAGCVEDTMAAIAGRTMHTFHTEGAGGGHAPDIIKVAGEHNILPASTNPTIPFTVNTEAEHMDMLMVCHHLDKSIKEDVQFADSRIRPQTIAAEDTLHDMGIFSITSSDSQAMGRVGEVITRTWQTADKNKKEFGRLKEEKGDNDNFRIKRYLSKYTINPAIAHGISEYVGSVEVGKVADLVLWSPAFFGVKPNMIIKGGFIALSQMGDANASIPTPQPVYYREMFAHHGKAKYDANITFVSKAAYDKGIKEELGLERQVLPVKNCRNITKKDMQFNDTTAHIEVNPETYHVFVDGKEVTSKPATKVSLAQLFSIF | 569 |
| 14 | Q8RNU6 | MKKISRKEYASMYGPTTGDKVRLGDTDLIAEVEHDYTIYGEELKFGGGKTLREGMSQSNNPSKEELDLIITNALIVDYTGIYKADIGIKDGKIAGIGKGGNKDMQDGVKNNLSVGPATEALAGEGLIVTAGGIDTHIHFISPQQIPTAFASGVTTMIGGGTGPADGTNATTITPGRRNLKWMLRAAEEYSMNLGFLAKGNASNDASLADQIEAGAIGFKIHEDWGTTPSAINHALDVADKYDVQVAIHTDTLNEAGCVEDTMAAIAGRTMHTFHTEGAGGGHAPDIIKVAGEHNILPASTNPTIPFTVNTEAEHMDMLMVCHHLDKSIKEDVQFADSRIRPQTIAAEDTLHDMGIFSITSSDSQAMGRVGEVITRTWQTADKNKKEFGRLKEEKGDNDNFRIKRYLSKYTINPAIAHGISEYVGSVEVGKVADLVLWSPAFFGVKPNMIIKGGFIALSQMGDANASIPTPQPVYYREMFAHHGKAKYDANITFVSQAAYDKGIKEELGLERQVLPVKNCRNITKKDMQFNDTTAHIEVNPETYHVFVDGKEVTLNQSIK | 559 |
| 15 | Q84F75 | MKKISRKEYVSMYGPTTGDKVRLGDTDLIAEVEHDYTIYGEELKFGGGKTLREGMSQSNNPSKEELDLIITNALIVDYTGIYKADIGIKDGKIAGIGKGGNKDMQDGVKNNLSVGPATEALAGEGLIVTAGGIDTHIHFISPQQIPTAFASGVTTMIGGGTGPADGTNATTITPGRRNLKWMLRAAEEYSMNLGFLAKGNASNDASLADQIEAGAIGFKIHEDWGTTPSAINHALDVADKYDVQVAIHTDTLNEAGCVKDTMAAIAGRTMHTFHTEGAGGGHAPDIIKVAGEHNILPASTNPTIPFTVNPEAEHMDMLMVCHHLDKSIKEDVQFADSRIRPQTIAAEDTLHDMGIFSITSSDSQAMGRVGEVITRTWQTADKNKKEFGRLKEEKGDNDNFRIKRYLSKYTINPAIAHGISEYVGSVEVGKVADLVLWSPAFFGVKPNMIIKGGFIALSQMGDANASIPTPQPVYYREMFAHHGKAKYDANITFVSQAAYDKGIKEELGLERQVLPVKNCRNITKKDMQFNDTTAHIEVNPETYRVFVDGKEVTSKPANKVSLAQLFSIF | 569 |
| 16 | Q9AFB1 | MKKISRKEYVSMYGPTTGDKVRLGDTDLIAEVEHDYTIYGEELKFGGGKTLREGMSQSNNPSKEELDLIITNALIVDYTGIYKADIGIKDGKIAGIGKGGNKDMQDGVKNNLSVGPATEALAGEGLIVTAGGIDTHIHFISPQQIPTAFASGVTTMIGGGTGPADGTNATTITPGRRNLKWMLRAAEEYSMNLSFLAKGNASNDASLADQIEAGAIGFKIHEDWGTTPSAINHALDVADKYDVQVAIHTDTLNEAGCVEDTMAAIAGRTMHTFHTEGAGGGHAPDIIKVAGEHNILPASTNPTIPFTVNTEAEHMDMLMVCHHLDKSIKEDVQFADSRIRPQTIAAEDTLHDMGIFSITSSDSQAMGRVGEVITRTWQTADKNKKEFGRLKEEKGDNDNFRIKRYLSKYTINPAIAHGISEYVGSVEVGKVADLVLWSPAFFGVKPNMIIKGGFIALSQMGDANASIPTPQPVYYREMFAHHGKAKYDANITFVSQAAYDKGIKEELGLERQVLPVKNCRNITKKDMQFNDTTAHIEVNPETYHVFVDGKEVTSKPANKVSLAQLFSIF | 569 |
| 17 | A0A0K2XPU4 | MKKISRKEYVSMYGPTTGDKVRLGDTDLILEVEHDYTTYGEEIKFGGGKTIRDGMGQTNSPSSHELDLVITNALIVDYTGIYKADIGIKNGKIHGIGKAGNKDLQDGVCNRLCVGPATEALAAEGLIVTAGGIDTHIHFISPQQIPTAFASGITTMIGGGTGPADGTNATTITPGRWNLKEMLRASEEYAMNLGYLGKGNVSFEPALIDQLEAGAIGFKIHEDWGSTPSAINHALNIADK  YDVQVAIHTDTLNEAGCVEDTLEAIAGRTIHTFHTEGAGGGHAPDVIKMAGEFNILPASTNPTIPFTKNTEAEHMDMLMVCHHLDKNIKEDVEFADSRIRPQTIAAEDKLHDMGIFSITSSDSQAMGRVGEVITRTWQTADKNKKEFGRLPEEKGDNDNFRIKRYISKYTINPAITHGISEYVGSVEVGKYADLVLWSPAFFGIKPNMIIKGGFIALSQMGDANASIPTPQPVYYREMFGHHGKAKYDTNITFVSQVAYENGIKHELGLQRVVLPVKNCRNITKKDLKFNDVTAHIEVNPETYKVKVDGNEVTSHAADKLSLAQLYNLF | 569 |
| 18 | A0A3D8J5B0 | MIKITRKQYASMYGPTIGDKVRLGDTNLFAEIEKDFTLYGEEIKFGGGKTIRDGMAQSVSSHNDSLDVVITNAIIIDYCGIYKADIGIKEGKIVGVGKAGNPDIQDGIHSTMVVGANTEAIAGEGLIVTAGGIDTHIHFISPTQIPTALYSGITTMIGGGTGPTAGTNATTCTPGKYNIKQMLRSIEGYAMNFGLLGKGNSSNENALESQIKAGALGLKIHEDWGSTPAVINHALNVAEKYDVQVAIHTDTLNESGCVEDTLNAIAGRTIHTFHTEGAGGGHAPDIIKAAGEMNILPASTNPTIPFTKNTADEHLDMLMVCHHLDKHIKEDVSFADSRIRPETIAAEDALHDMGIFSITSSDSQAMGRVGEVIIRTWQMADKCKNEFGPLKEEKDDNDNFRIKRYIAKYTINPAVAHGISEYVGSIEEGKFADLVLWKPSMFGVKPEMIIKNGMVVAAKMGDSNASIPTPEPVTYTKMFGSYGKAKYDCGITFVSKIAYDSNIKEKLGLERVVVPVKNCRNITKKDMKHNDVTAQIEVNPETYEVKVNGVKITSKPVNKVSLGQLYHLF | 569 |
| 19 | A0A4U8T815 | MAKISRKDYVAMYGPTLGDKIRLGDTDLFAEIEKDYAIYGEEIKFGGGKSIRDGMAQSVNEGEDALDLVITNAVIIDYSGIIKADIGIKAGKIVGIGKAGNKDTQDGVDSNMIVGASTEVIAGEGLIVTAGGIDTHIHFIAPQQIPTALYSGITTMIGGGVGPTAGTSATTCTPGKWNLEQMLKAAEEYTMNLGFFGKGNSSSEAALSKQILSGALGLKIHEDWGSTPAVINHALNVADK  YDVQVAIHTDTLNEAGCVEDTLKAINGRTIHTFHTEGAGGGHAPDIIKVAGELNILPASTNPTIPYTKNTIDEHLDMLMVCHHLDKRIKEDVAFADSRIRPESIAAEDTLHDLGIFSITSSDSQAMGRVGEVITRTWQLADKNKKEFGRLKEECGDNDNFRIKRYVAKYTINPAIAHGIGSYVGSIEVGKYADLVLWKPAFFGAKPEMIIKNGMVAASKMGDINASIPTPQPVIYRPSFGHHGKAKFNTSITFVSQISYELGIKEKLGLKRIVLPIKNCRNISKKDMLYNDVTAHIEVNPQTYEIKVNGKKITSKWVEKVSLGQLYYLF | 569 |
| 20 | A0A4U8U8N0 | MIKINRQEYVSMYGPTTGDKIRLGDTELFAEIEKDYAIYGEEIKFGGGKTIRDGMAQSVSDSENELDSVITNAVIIDYTGIYKADIGIKNGKIFGIGKAGNKDTQDGVCDKLIVGTNTEVIAGEGLIVTAGGIDTHIHYISPTQIPTALYSGVTTMIGGGTGPAAGTSATTCTPGSWHMREMIRATQHYAMNFGFFGKGNSSNENALSKQIESGALGLKVHEDWGSTPAAINHALSIADKYDVQIAIHTDTLNEAGCMEDTLQAINGRTIHTFHTEGAGGGHAPDIIKAAGELHVLPASTNPTIPFTTNTADEHLDMLMVCHHLDKNIKEDVAFADSRIRPETIAAEDTLHDMGIFSITSSDSQAMGRVGEVIIRTWQTADKCKREFGALKEEKGNNDNFRIKRYIAKYTINPAIAHGIADYVGSIEIGKIADLVIWKPSMFGVKPEMILKNGMIVAAKIGDSNASIPTPQPIVYADMFGSVGSAKYDCGFTFVSKVAFDSNIKEKYGIERNILPVKNCRNITKKDMKYNDVVEKIEVDSETYEVKINGVKITSKPVSKVSLGQLYTLF | 569 |
| 21 | A0A0K2Y350 | MKKISRKEYVSMYGPTTGDKVRLGDTDLILEVEHDCTTYGEEIKFGGGKTIRDGMGQTNSPSSHELDLVITNALIVDYTGIYKADIGIKNGKIHGIGKAGNKDLQDGVCNRLCVGPATEALAGEGLIVTAGGIDTHIHFISPQQIPTAFASGITTMIGGGTGPADGTNATTITPGRWNLKEMLRASEEYAMNLGYLGKGNVSYEPSLTDQLYAGAIGFKIHEDWGSTPSAINHALNIADK  YDVQVAIHTDTLNEAGCVEDTLEAIAGRTIHTFHTEGAGGGHAPDVIKMAGEFNILPASTNPTIPFTKNTEAEHMDMLMVCHHLDKNIKEDVEFADSRIRPQTIAAEDKLHDMGIFSITSSDSQAMGRVGEVITRTWQTADKNKKEFGRLPEEKGDNDNFRIKRYIAKYTINPAIAHGISEYVGSVEVGKYADLVLWSPAFFGIKPNMIIKGGFIALSQMGDANASIPTPQPVYYREMFG  HHGKAKFDTNITFVSQVAYENGIKEELGLQRIVLPVKNCRNITKKDLKFNDVTAHIEVNPETYKVKVDGNEVTSHAADKLPLAQLYNLF | 569 |
| 22 | A0A0K2YAN9 | MKKISRKEYVSMYGPTTGDKVRLGDTDLILEVEHDYTTYGEEIKFGGGKTIRDGMGQTNSPSSHELDLVITNALIVDYTGIYKADIGIKNGKIHGIGKAGNKDLQDGVCNRLCVGPATEALAAEGLIVTAGGIDTHIHFISPQQIPTAFASGITTMIGGGTGPADGTNATTITPGRWNLKEMLRASEEYAMNLGYLGKGNVSFEPALIDQLEAGAIGFKIHEDWGSTPSAINHALNIADK  YDVQVAIHTDTLNEAGCVEDTLEAIAGRTIHTFHTEGAGGGHAPDVIKMAGEFNILPASTNPTIPFTKNTEAEHMDMLMVCHHLDKNIKEDVEFADSRIRPQTIAAEDKLHDMGIFSITSSDSQAMGRVGEVITRTWQTADKNKKEFGRLPEEKGDNDNFRIKRYISKYTINPAITHGISEYVGSVEVGKYADLVLWSPAFFGIKPNMIIKGGFIALSQMGDANASIPTPQPVYYREMFGHHGKAKFDTNITFVSQVAYENGIKHELGLQRVVLPVKNCRNITKKDLKFNDVTAHIEVNPETYKVKVDGNEVTSQAADKLSLAQLYNLF | 569 |
| 23 | A0A2U8FEJ6 | MIKISRKQYVSMYGPTTGDKVRLGDTNLFAEIEKDYTTYGEEIKFGGGKTIRDGMAQSASVYENTLDVVITNAMIIDYTGIYKADIGIKNGKIVGIGKAGNPDIQDNVFQTMVVGTATEVIAGEGQILTAGGIDTHIHFISPTQIPTALYSGITTMIGGGTGPAAGTNATTCTPGKWNIQQMLRAAEEYAMNLGFLGKGNSSNEEALEEQITSGALGLKIHEDWGSTPAAINHALNVAEK  YDVQVAIHTDTLNEAGCVEDTMRAIRGRTIHTFHTEGAGGGHAPDIIKAAGELNILPASTNPTIPFTKNTADEHLDMLMVCHHLDKKIKEDVAFADSRIRPETIAAEDTLHDMGIFSITSSDSQAMGRVGEVIIRTWQTADKCKNEFGALKEECGENDNFRIKRYISKYTINPAIAHGISEYVGSVEVGKFADLVLWKPSMFGVKPEMILKNGMIVAAKIGDCNASIPTPEPVVYAPMFG  SYGKAKYDCGITFVSKIAYDTNIKEKFGLQRILLPVKNCRKITKKDMKFNDVVTPIKVNPQTYEVSVKGKKITSKFVDKVSLGQLYSLF | 569 |
| 24 | A0A6D2CBQ4 | MTKINRQEYVSMYGPTTGDKIRLSDTELFAEIEKDYAIYGEEIKFGGGKTIRDGMAQSVSNSENELDSVITNAVIIDYTGIYKADIGIKNGKIFGIGKAGNKDTQDGVCDKLIVGTNTEVIAGEGLIVTAGGIDTHIHYISPTQIPTALYSGVTTMIGGGTGPAAGTSATTCTPGSWHMREMIRATQHYAMNFGFFGKGNSSNENALSKQIESGALGLKVHEDWGSTPAAINHALNIADK  YDVQIAIHTDTLNEAGCMEDTLQAINGRTIHTFHTEGAGGGHAPDIIKAAGELHVLPASTNPTIPFTTNTADEHLDMLMVCHHLDKNIKEDVAFADSRIRPETIAAEDTLHDMGIFSITSSDSQAMGRVGEVIIRTWQTADKCKREFGALKEERGDNDNFRIKRYIAKYTINPAIAHGIADYVGSVEIGKIADLVIWKPSMFGVKPEMILKNGMIVAAKIGDSNASIPTPQPIVYADMFGSVGSARYDCGFTFVSKVAFDSNIKEKYGIERNILPVKNCRNITKKDMKYNDVVEKIEVDSETYEVKVNGVKITSKPISKVSLGQLYTLF | 569 |
| 25 | A0A2W6MUB9 | MFKVSRAEYASHFGITKGDSIRLADTNLFARVEKDYAIYGEECKFGGGKTLRDGMGQNSRMLDKDVVDLIITNALVIDYTGIYKADIGIKDGLIYGIGKGGNPDIMDGVDFVTGVSTEALAGEGLILTAGGVDTHIHFINPEQVNEALSNGVTTLFGGGTGPNDGSKATTCTPGKFHIKRMLQGTDDLPINIGLYGKGHGSNVEVNLEQLRAGAAGLKIHEDWGTTKSAIDNALKAAEIADVSVGIHTDTLNEFGCVEDTRAAIGGRTIHTFHTEGAGGGHAPDIIKLAGDANILPASTNPTMPFTTNTIEEHLDMLMVCHHLSKNVKEDVAFADSRIRKETIGAEDVLHDLGAISIMSSDSQAMGRIGEVVSRTWQTAHKMKEQRGAMEGDSEYCDNNRIKRYIAKYTINPAIAAGVSEYVGSVEVGKIADLVLWDPKSFGTKPKMVIKGGFCALSVMGDSNASIPTPEPIMHRKMFGAIGEAVHRTCYSFMCKMAVEANVAEEYGIKKKVLPIKGAAHAKKADMKLNNATPKIDVDPQTYVVSVDGKEAYSEPVSELPLAQRYYLF | 568 |
| 26 | A0A4U8SCC0 | MIKIKRQEYVSMYGPTTGDKIRLGDTELFAEIEKDYAVYGEEIKFGGGKTIRDGMAQSVSDSENELDTVITNAVIIDYTGIYKADIGIKNGKIAGIGKAGNKDTQDGVCDKLIVGANTEAIAGEGLIVTAGGIDTHIHFISPTQVPTALYSGITTMIGGGTGPAAGTSATTCTPGSWHIREMIRATQQYAMNFGFFGKGNSSNEKALSEQVEAGALGLKIHEDWGSTPAAINHALKIADRYDVQVAIHTDTLNEAGCMEDTLEAINGRTIHTFHTEGAGGGHAPDIIKAAGELNVLPASTNPTIPFTKNTADEHLDMLMVCHHLDKNIKEDVAFADSRIRPETIAAEDTLHDMGIFSITSSDSQAMGRVGEVIMRTWQTADKCKREFGALKEEEGKNDNFRIKRYIAKYTINPAIAHGIADYVGSVETDKIADLVLWKPSMFGVKPEMIIKNGMIVAAKMGDSNASIPTPQPVVYTDMFGSIGSAKYDCGFTFVSKVAFDSNIKEKYGIERNVLPVKNCRNITKRDMKHNDVVEKIEVDSETYEVRVRGEKITSKPIDKVSLGQLYTLF | 569 |
| 27 | A0A553UL00 | MKKISRKEYVSMYGPTTGDKVRLGDTDLILEVEHDCTTYGEEIKFGGGKTIRDGMAQTNSPSSHELDLVITNALIVDYTGIYKADIGIKNGKIHGIGKAGNKDMQDGVCNNLCVGPATEALAAEGLIVTAGGIDTHIHFISPQQIPTAFASGITTMIGGGTGPADGTNATTITPGRWNLKTMLRASEEYAMNLGYLGKGNVSYEPSLVDQLEAGAIGFKIHEDWGSTPAAIHHCLNVADKYDVQVAIHTDTLNEAGCVEDTLQAIAGRTIHTFHTEGAGGGHAPDVIKMSGEFNILPASTNPTIPFTVNTEAEHMDMLMVCHHLDKNIKEDVQFADSRIRPQTIAAEDKLHDMGIFSITSSDSQAMGRVGEVITRTWQTADKNKKEFGRLPEEKGDNDNFRIKRYISKYTINPAIAHGISEYVGSVEVGKFADLVLWSPAFFGIKPNMIIKGGFIALSQMGDANASIPTPQPVYYREMFGHHGKAKFDTNITFVSQVAYDNGIKEELGLQRVVLPVKNCRNITKKDLKFNDVTAHIEVNPETYKVKVDGKEVTSKAADKISLAQLYNLF | 569 |
| 28 | A0A0K2XW58 | MKKISRKEYVSMYGPTTGDKVRLGDTDLILEVEHDYTTYGEEIKFGGGKTIRDGMGQTNSPSSHELDLVITNALIVDYTGIYKADIGIKNGKIHGIGKAGNKDLQDGVCNRLCVGPATEALAAEGLIVTAGGIDTHIHFISPQQIPTAFASGITTMIGGGTGPADGTNATTITPGRWNLKEMLRASEEYAMNLGYLGKGNVSFEPALIDQLEAGAIGFKIHEDWGSTPSAINHALNIADK  YDVQVAIHTDTLNEAGCVEDTLEAIAGRTIHTFHTEGAGGGHAPDVIKMAGEFNILPASTNPTIPFTKNTEAEHMDMLMVCHHLDKNIKEDVEFADSRIRPQTIAAEDKLHDMGIFSITSSDSQAMGRVGEVITRTWQTADKNKKEFGRLPEEKGDNDNFRIKRYISKYTINPAITHGISEYVGSVEVGKFADLVLWSPAFFGIKPNMIIKGGFIALSQMGDANASIPTPQPVYYREMFGHHGKAKFDTNITFVSQVAYENGIKHELGLQRIVLPVKNCRNITKKDLKFNDVTAHIEVNPETYKVKVDGNEVTSHAADKLSLAQLYNLF | 569 |
| 29 | A0A3D8IMT4 | MTKISRKEYVAMYGPTKGDKIRLADTDLYAEIEKDYAVYGEEIKFGGGKTIRDGMAQSATYNENMLDVVITNAVIIDYSGIYKADIGIKEGKIIGIGKAGNRDMQEGVHSNMIVGAGTEVIAGEGLIVTAGGIDTHIHFIAPQQIPTALYSGVTTMIGGGTGPAAGSNATTCTPGEWNMHEMLKAAEEYTMNLGFFGKGNTSDEKSLYNQLQAGALGFKIHEDWGSTPSAINHALNIAEQYDVQAAIHTDTLNEGGCVEDTLKAINGRTIHTFHTEGAGGGHAPDIIKAAGELFVLPASTNPTIPYTKNTADEHLDMLMVCHHLDKRIKEDVAFADSRIRPETIAAEDVLHDMGIFSITSSDSQAMGRVGEVITRTWQTADKNKKEFGRLPQECGENDNFRIKRYIAKYTINPAIACGISSYVGSVEVGKYADLVLWKPAFFGSKPEMIIKNGMIVGSKIGDFNASIPTPEPVVYHEMFG  HHGKAKFDTSITFVSKIAYESGIKEKLGLQRKILPVQNCRNITKKDMQYNDVVANIEVDSETYEVKVNGKKVTSRCVDKVSLGQLYYLF | 569 |
| 30 | A0A3D8JBL5 | MIKISRKEYASMYGPTVGDKVRLGDTELFAEIEKDFTIYGEEIKFGGGKTIRDGMAQSVSSNENELDSVITNAMIIDYTGIYKADIGLKDGKIAGIGKAGNKDTQDGVNDNMIVGTGTEVIAGEGLIVTAGGIDTHIHYISPTQIPTALYSGVTTMIGGGTGPAAGTFATTCTPGKWHMKQMLRSTEEYAMNFGFFGKGNSSNEEALGEQIKAGALGLKVHEDWGSTPAAINHALNIAEKYDVQVAIHTDTLNEAGCIEDTLNAIAGRTIHTFHTEGAGGGHAPDIIKAAGEANILPASTNPTIPFTKNTADEHLDMLMVCHHLDKNIKEDVAFADSRIRPETIAAEDTLHDMGIFSITSSDSQAMGRVGEVITRTWQTADKCKKEFGRLKEETGENDNFRIKRYISKYTINPAIAHGISEYVGSIEVGKIADLILWKPSMFGVKPETIVKCGMIVASKMGDSNASIPTPEPIVYTDMFGSIGKAKYDCGVTFVSKVSCELNIKENFGLDRTLLPVKNCRNVTKKDMKFNDVITPIEVNPETYEVKVEGKKITSKFVDEVSLGQLYNLF | 569 |
| 31 | A0A4U8TET8 | MIKIKRQEYVSMYGPTTGDKIRLGDTELFAEIEKDYAVYGEEIKFGGGKTIRDGMAQSVSDSENELDTVITNAVIIDYTGIYKADIGIKNGKIAGIGKAGNKDTQDGVCDKLIVGANTEAIAGEGLIVTAGGIDTHIHFISPTQVPTALYSGITTMIGGGTGPAAGTSATTCTPGSWHIREMIRATQQYAMNFGFFGKGNSSNEKALSEQVEAGALGLKIHEDWGSTPAAINHALKIADKYDVQVAIHTDTLNEAGCMEDTLEAINGRTIHTFHTEGAGGGHAPDIIKAAGELNVLPASTNPTIPFTKNTADEHLDMLMVCHHLDKNIKEDVAFADSRIRPETIAAEDALHDMGIFSITSSDSQAMGRVGEVIMRTWQTADKCKREFGALKEEEGEHDNFRIKRYIAKYTINPAIAHGIADYVGSVETDKIADLVLWKPSMFGVRPEMILKNGMIVAAKMGDSNASIPTPQPVVYTDMFGSIGSAKYDCGFTFVSKVAFDSNIKEKYGIERNILPVKNCRNITKKDMKYNDVVEKIEVDSETYEVRVRGEKITSKPIDKVSLGQLYTLF | 569 |
| 32 | A0A4U8UDY1 | MIKISRKQYVSMYGPTTGDKVRLGDTNLFAEIEKDYTTYGEEIKFGGGKTIRDGMAQSASMYENVLDVVITNAMIIDYTGIYKADIGIKNGKIIGIGKAGNPDIQDNVFQTMVVGAATEVIAGEGQILTAGGIDTHIHFISPTQIPTALYSGITTMIGGGTGPAAGTNATTCTPGKWNIQQMLRAAEEYAMNLGFLGKGNSSNEEALEEQITSGALGLKIHEDWGSTPAAINHALNVAEK  YDVQVAIHTDTLNEAGCVEDTMRAIRGRTIHTFHTEGAGGGHAPDIIKAAGELNILPASTNPTIPFTKNTADEHLDMLMVCHHLDKKIKEDVAFADSRIRPETIAAEDTLHDMGIFSITSSDSQAMGRVGEVIIRTWQTADKCKNEFGALKEECGENDNFRIKRYISKYTINPAIAHGISEYVGSVEVGKFADLVLWKPSMFGVKPEMILKNGMIVAAKMGDCNASIPTPEPVVYAPMFG  SYGKAKYDCGITFVSKIAYDTNIKEKFGLQRILLPVKNCRKITKKDMKFNDVVTPIKVNPQTYEVSVKGKKITSKFVDKVSLGQLYSLF | 569 |
| 33 | A0A6N4RAN0 | MIKINRQEYVSMYGPTTGDKIRLGDTELFAEIEKDYAIYGEEIKFGGGKTIRDGMAQSVSDSENELDSVITNAVIIDYTGIYKADIGIKNGKIFGIGKAGNKDTQDGVCDKLIVGTNTEVIAGEGLIVTAGGIDTHIHYISPTQIPTALYSGVTTMIGGGTGPAAGTSATTCTPGSWHMREMIRATQHYAMNFGFFGKGNSSNENALSKQIESGALGLKVHEDWGSTPAAINHALSIADK  YDVQIAIHTDTLNEAGCMEDTLQAINGRTIHTFHTEGAGGGHAPDIIKAAGELHVLPASTNPTIPFTTNTADEHLDMLMVCHHLDKNIKEDVAFADSRIRPETIAAEDTLHDMGIFSITSSDSQAMGRVGEVIIRTWQTADKCKREFGALKEEKGNNDNFRIKRYIAKYTINPAIAHGIADYVGSVEIGKIADLVIWKPSMFGVKPEMILKNGMIVAAKIGDSNASIPTPQPIVYADMFGSVGSARYDCGFTFVSKVAFDSNIKEKYGIERNILPVKNCRNITKKDMKYNDVVEKIEVDSETYEVKINGVKITSKPVSKVSLGQLYTLF | 569 |
| 34 | A0A060Q3H2 | MKKISRKEYVSMYGPTTGDKVRLGDTDLILEVEHDCTTYGEEIKFGGGKTIRDGMGQTNSPSSHELDLVITNALIVDYTGIYKADIGIKDGKIHGIGKAGNKDIQDGVCNRLCVGPATEALAGEGLIVTAGGIDTHIHFISPQQIPTAFASGITTMLGGGTGPADGTNATTITPGRWNLKEMLRASEEYAMNLGYMGKGNVSYEPSLVEQLEAGAIGFKIHEDWGSTPSAIHHALKIADE  YDVQVAIHTDTLNEAGCVEDTLEAIAGRTIHTFHTEGAGGGHAPDVIKMAGAFNVLPASTNPTIPFTKNTEAEHMDMLMVCHHLDKNIKEDVEFA | 335 |
| 35 | E7G2Y5 | MRKGQAMKKISRKEYVSMYGPTTGDKVRLGDTDLILEVEHDCTTYGEEIKFGGGKTIRDGMGQTNSPSSHELDLVITNALIVDYTGIYKADIGIKDGKIHGIGKAGNKDIQDGVCNRLCVGPATEALAGEGLIVTAGGIDTHIHFISPQQIPTAFASGITTMLGGGTGPADGTNATTITPGRWNLKEMLRASEEYAMNLGYMGKGNVSYEPSLVEQLEAGAIGFKIHEDWGSTPSAIHHALKIADEYDVQVAIHTDTLNEAGCVEDTLEAIAGRTIHTFHTEGAGGGHAPDVIKMAGAFNVLPASTNPTIPFTKNTEAEHMDMLMVCHHLDKNIKEDVEFADSRIRPQTIAAEDKLHDMGIFSITSSDSQAMGRVGEVITRTWQTADKNKKEFGRLKEETGDNDNFRIKRYISKYTINPAIAHGISEYVGSVEVGKYADLVLWSPAFFGIKPNMIIKGGMIALSQMGDANASIPTPQPVYYREMFGHHGKAKFDTNITFVSRVAYENGIKHELGLQRKVLPVKNCRNITKKDLKFNDVTAHIEVNPETYKVKVDGQEVTSKAADKISLAQLYNLF | 575 |
| 36 | G2M8T5 | MKKLDYVNTYGPTKGDKVRLGDTDLWAEVEHDYTIYGEELKFGAGKTIRECMGQSNSHDENTLDLVITNALIIDYTGIYKADIGIKNGKIAGIGKAGNKDMQDGVSPNLVVGVGTEALAGEGMIVTAGGIDSHTHFLSPQQFPTALANGVTTMFGGGTGPVDGTNATTITPGEWNIHRMLRAAEEYAMNVGFLGKGNSSSKTQLVEQIEAGIVGFKLHEDWGTTPSAIDTCLSVADEYDVQVCIHTDTVNEAGYVEDTLNAMNGRAIHAYHIEGAGGGHSPDVITMAGEENILPSSTTPTIPYTINTVAEHLDMLMTCHHLDKKIREDLQFSQSRIRPGSIAAEDVLHDNGMIAMTSSDSQAIGRAGEVVPRTWQTADKNKKEFGPLKEYDQNGNDNFRIKRYISKYTINPAITHGVSEYIGSVEAGKIADLVVWNPAFFGVKPKIIIKGGLVVFSEMGDSNASVPTPQPVYYREMFGHHGKAKFDTSITFVNKLAYEKGIKEKLGLERQVLPIKNVRNITKKDFKFNNTIGKLTVDPKTFEVFLDGKLCTSKPASELPLAQRYTFF | 567 |
| 37 | G2MD09 | MKKISRKEYVSMYGPTTGDKVRLGDTDLIAEVEHDYTIYGEELKFGGGKTLREGMSQSNNPSKEELDLIITNALIVDYTGIYKADIGIKDGKIAGIGKGGNKDMQDGVKNNLSVGPATEALAGEGLIVTAGGIDTHIHFISPQQIPTAFASGVTTMIGGGTGPADGTNATTITPGRRNLKWMLRAAEEYSMNLGFLAKGNTSNDASLADQIEAGAIGFKIHEDWGTTPSAINHALDVADKYDVQVAIHTDTLNEAGCVEDTMAAIAGRTMHTFHTEGAGGGHAPDIIKVAGEHNILPASTNPTIPFTVNTEAEHMDMLMVCHHLDKSIKEDVQFADSRIRPQTIAAEDTLHDMGIFSITSSDSQAMGRVGEVITRTWQTADKNKKEFGRLKEEKGDNDNFRIKRYLSKYTINPAIAHGISEYVGSVEVGKVADLVLWSPAFFGVKPNMIIKGGFIALSQMGDANASIPTPQPVYYREMFAHHGKAKYDANITFVSQAAYDKGIKEELGLERQVLPVKNCRNITKKDMQFNDTTAHIEVNPETYHVFVDGKEVTSKPANKVSLAQLFSIF | 569 |
| 38 | H8H6Q7 | MKKISRKEYVSMYGPTTGDKVRLGDTDLIAEVEHDYTIYGEELKFGGGKTLREGMSQSNNPSKEELDLIITNALIVDYTGIYKADIGIKDGKIAGIGKGGNKDMQDGVKNNLSVGPATEALAGEGLIVTAGGIDTHIHFISPQQIPTAFASGVTTMIGGGTGPADGTNATTITPGRRNLKWMLRAAEEYSMNLGFLAKGNASNDASLADQIEAGAIGFKIHEDWGTTPSAINHALDVADKYDVQVAIHTDTLNEAGCVEDTMAAIAGRTMHTFHTEGAGGGHAPDIIKVAGEHNILPASTNPTIPFTVNTEAEHMDMLMVCHHLDKSIKEDVQFADSRIRPQTIAAEDTLHDMGIFSITSSDSQAMGRVGEVITRTWQTADKNKKEFGRLKEEKGDNDNFRIKRYLSKYTINPAIAHGISEYVGSVEVGKVADLVLWSPAFFGVKPNMIIKGGFIALSQMGDANASIPTPQPVYYREMFAHHGKAKYDANITFVSQAAYDKGIKEELGLERQVLPVKNCRNITKKDMQFNDTTAHIEVNPETYHVFVDGKEVTSKPANKVSLAQLFSIF | 569 |
| 39 | I0EG04 | MKKISRKEYASMYGPTTGDKVRLGDTDLIAEVEHDYTIYGEELKFGGGKTLREGMSQSNNPSKEELDLIITNALIVDYTGIYKADIGIKDGKIAGIGKGGNKDMQDGVKNNLSVGPATEALAGEGLIVTAGGIDTHIHFISPQQIPTAFASGVTTMIGGGTGPADGTNATTITPGRRNLKFMLRAAEEYSMNFGFLAKGNVSNDASLADQIEAGAIGFKIHEDWGTTPSAINHALDVADKYDVQVAIHTDTLNEAGCVEDTMAAIAGRTMHTFHTEGAGGGHAPDIIKVAGEHNILPASTNPTIPFTVNTEAEHMDMLMVCHHLDKSIKEDVQFADSRIRPQTIAAEDTLHDMGIFSITSSDSQAMGRVGEVITRTWQTADKNKKEFGRLKEEKGDNDNFRIKRYLSKYTINPAIAHGISEYVGSVEVGKVADLVLWSPAFFGVKPNMIIKGGFIALSQMGDANASIPTPQPVYYREMFAHHGKAKYDANITFVSQAAYDKGIKEELGLERQVLPVKNCRNITKKDMQFNDTTAHIEVNPETYHVFVDGKEVTSKPANKVSLAQLFSIF | 569 |
| 40 | I2DEC3 | MKKISRKEYVSMYGPTTGDKVRLGDTDLIAEVEHDYTIYGEELKFGGGKTLREGMSQSNNPSKEELDLIITNALIVDYTGIYKADIGIKDGKIAGIGKGGNKDMQDGVKNNLSVGPATEALAGEGLIVTAGGIDTHIHFISPQQIPTAFASGVTTMIGGGTGPADGTNATTITPGRRNLKWMLRAAEEYSMNLGFLAKGNASNDASLADQIEAGAIGFKIHEDWGTTPSAINHALDVADKYDVQVAIHTDTLNEAGCVEDTMAAIAGRTMHTFHTEGAGGGHAPDIIKVAGEHNILPASTNPTIPFTVNTEAEHMDMLMVCHHLDKSIKEDVQFADSRIRPQTIAAEDTLHDMGIFSITSSDSQAMGRVGEVITRTWQTADKNKKEFGRLKEEKGDNDNFRIKRYLSKYTINPAIAHGISEYVGSVEVGKVADLVLWSPAFFGVKPNMIIKGGFIALSQMGDANASIPTPQPVYYREMFAHHGKAKYDANITFVSQAAYDKGIKEELGLERQVLPVKNCRNITKKDMQFNDTTAHIEVNPETYHVFVDGKEVTSKPATKVSLAQLFSIF | 569 |
| 41 | M3NUN6 | MKKISRKEYVSMYGPTTGDKVRLGDTDLIAEVEHDYTIYGEELKFGGGKTLREGMSQSNNPSKEELDLIITNALIVDYTGIYKADIGIKDGKIAGIGKGGNKDMQDGVKNNLSVGPATEALAGEGLIVTAGGIDTHIHFISPQQIPTAFASGVTTMIGGGTGPADGTNATTITPGRRNLKWMLRAAEEYSMNLGFLAKGNTSNDASLADQIEAGAIGFKIHEDWGTTPSAINHALDVADKYDVQVAIHTDTLNEAGCVEDTMAAIAGRTMHTFHTEGAGGGHAPDIIKVAGEHNILPASTNPTIPFTVNTEAEHMDMLMVCHHLDKSIKEDVQFADSRIRPQTIAAEDTLHDMGIFSITSSDSQAMGRVGEVITRTWQTADKNKKEFGRLKEEKGDNDNFRIKRYLSKYTINPAIAHGISEYVGSVEVGKVADLVLWSPAFFGVKPNMIIKGGFIALSQMGDANASIPTPQPVYYREMFAHHGKAKYDANITFVSQAAYDKGIKEELGLERQVLPVKNCRNITKKDMQFNDTTAHIEVNPETYHVFVDGKEVTSKPANKVSLAQLFSIF | 569 |
| 42 | M3PTF9 | MKKISRKEYVSMYGPTTGDKVRLGDTDLIAEVEHDYTIYGEELKFGGGKTLREGMSQSNNPSKEELDLIITNALIVDYTGIYKADIGIKDGKIAGIGKGGNKDMQDGVKNNLSVGPATEALAGEGLIVTAGGIDTHIHFISPQQIPTAFASGVTTMIGGGTGPADGTNATTITPGRRNLKWMLRAAEEYSMNLGFLAKGNTSNDASLADQIEAGAIGFKIHEDWGTTPSAINHALDVADKYDVQVAIHTDTLNEAGCVEDTMAAIAGRTMHTFHTEGAGGGHAPDIIKVAGEHNILPASTNPTIPFTVNTEAEHMDMLMVCHHLDKSIKEDVQFADSRIRPQTIAAEDTLHDMGIFSITSSDSQAMGRVGEVITRTWQTADKNKKEFGRLKEEKGDNDNFRIKRYLSKYTINPAIAHGISEYVGSVEVGKVADLVLWSPAFFGVKPNMIIKGGFIALSQMGDANASIPTPQPVYYREMFAHHGKAKYDANITFVSQAAYDKGIKEELGLERQVLPVKNCRNITKKDMQFNDTTAHIEVNPETYHVFVDGKEVTSKPANKVSLAQLFSIF | 569 |
| 43 | M3PWK4 | MKKISRKEYVSMYGPTTGDKVRLGDTDLIAEVEHDYTIYGEELKFGGGKTLREGMSQSNNPSKEELDLIITNALIVDYTGIYKADIGIKDGKIAGIGKGGNKDMQDGVKNNLSVGPATEALAGEGLIVTAGGIDTHIHFISPQQIPTAFASGVTTMIGGGTGPADGTNATTITPGRRNLKWMLRAAEEYSMNLGFLAKGNTSNDASLADQIEAGAIGFKIHEDWGTTPSAINHALDVADKYDVQVAIHTDTLNEAGCVEDTMAAIAGRTMHTFHTEGAGGGHAPDIIKVAGEHNILPASTNPTIPFTVNTEAEHMDMLMVCHHLDKSIKEDVQFADSRIRPQTIAAEDTLHDMGIFSITSSDSQAMGRVGEVITRTWQTADKNKKEFGRLKEEKGDNDNFRIKRYLSKYTINPAIAHGISEYVGSVEVGKVADLVLWSPAFFGVKPNMIIKGGFIALSQMGDANASIPTPQPVYYREMFAHHGKAKYDANITFVSQAAYDKGIKEELGLERQVLPVKNCRNITKKDMQFNDTTAHIEVNPETYHVFVDGKEVTSKPANKVSLAQLFSIF | 569 |
| 44 | M3QY21 | MKKISRKEYVSMYGPTTGDKVRLGDTDLIAEVEHDYTIYGEELKFGGGKTLREGMSQSNNPSKEELDLIITNALIVDYTGIYKADIGIKDGKIAGIGKGGNKDMQDGVKNNLSVGPATEALAGEGLIVTAGGIDTHIHFISPQQIPTAFASGVTTMIGGGTGPADGTNATTITPGRRNLKWMLRAAEEYSMNLGFLAKGNASNDASLADQIEAGAIGFKIHEDWGTTPSAINHALDVADKYDVQVAIHTDTLNEAGCVEDTMAAIAGRTMHTFHTEGAGGGHAPDIIKVAGEHNILPASTNPTIPFTVNTEAEHMDMLMVCHHLDKSIKEDVQFADSRIRPQTIAAEDTLHDMGIFSITSSDSQAMGRVGEVITRTWQTADKNKKEFGRLKEEKGDNDNFRIKRYLSKYTINPAIAHGISEYVGSVEVGKVADLVLWSPAFFGVKPNMIIKGGFIALSQMGDANASIPTPQPVYYREMFAHHGKAKYDANITFVSQAAYDKGIKEELGLERQVLPVKNCRNITKKDMQFNDTTAHIEVNPETYHVFVDGKEVTSKPANKVSLAQLFSIF | 569 |
| 45 | G2M772 | MKKISRKEYVSMYGPTTGDKVRLGDTDLIAEVEHDYTIYGEELKFGGGKTLREGMSQSNNPSKEELDLIITNALIVDYTGIYKADIGIKDGKIAGIGKGGNKDMQDGVKNNLSVGPATEALAGEGLIVTAGGIDTHIHFISPQQIPTAFASGVTTMIGGGTGPADGTNATTITPGRRNLKWMLRAAEEYSMNLGFLAKGNASNDASLADQIEAGAIGFKIHEDWGTTPSAINHALDVADKYDVQVAIHTDTLNEAGCVEDTMAAIAGRTMHTFHTEGAGGGHAPDIIKVAGEHNILPASTNPTIPFTVNTEAEHMDMLMVCHHLDKSIKEDVQFADSRIRPQTIAAEDTLHDMGIFSITSSDSQAMGRVGEVITRTWQTADKNKKEFGRLKEEKGDNDNFRIKRYLSKYTINPAIAHGISEYVGSVEVGKVADLVLWSPAFFGVKPNMIIKGGFIALSQMGDANASIPTPQPVYYREMFAHHGKAKYDANITFVSQAAYDKGIKEELGLERQVLPVKNCRNITKKDMQFNDTTAHIEVNPETYHVFVDGKEVTSKPANKVSLAQLFSIF | 569 |
| 46 | M3NNG8 | MKKISRKEYVSMYGPTTGDKVRLGDTDLIAEVEHDYTIYGEELKFGGGKTLREGMSQSNNPSKEELDLIITNALIVDYTGIYKADIGIKDGKIAGIGKGGNKDMQDGVKNNLSVGPATEALAGEGLIVTAGGIDTHIHFISPQQIPTAFASGVTTMIGGGTGPADGTNATTITPGRRNLKWMLRAAEEYSMNLGFLAKGNTSNDASLADQIEAGAIGFKIHEDWGTTPSAINHALDVADKYDVQVAIHTDTLNEAGCVEDTMAAIAGRTMHTFHTEGAGGGHAPDIIKVAGEHNILPASTNPTIPFTVNTEAEHMDMLMVCHHLDKSIKEDVQFADSRIRPQTIAAEDTLHDMGIFSITSSDSQAMGRVGEVITRTWQTADKNKKEFGRLKEEKGDNDNFRIKRYLSKYTINPAIAHGISEYVGSVEVGKVADLVLWSPAFFGVKPNMIIKGGFIALSQMGDANASIPTPQPVYYREMFAHHGKAKYDANITFVSQAAYDKGIKEELGLERQVLPVKNCRNITKKDMQFNDTTAHIEVNPETYHVFVDGKEVTSKPANKVSLAQLFSIF | 569 |
| 47 | M3P4J9 | MKKISRKEYVSMYGPTTGDKVRLGDTDLIAEVEHDYTIYGEELKFGGGKTLREGMSQSNNPSKEELDLIITNALIVDYTG  IYKADIGIKDGKIAGIGKGGNKDMQDGVKNNLSVGPATEALAGEGLIVTAGGIDTHIHFISPQQIPTAFASGVTTMIGGG  TGPADGTNATTITPGRRNLKWMLRAAEEYSMNLGFLAKGNASNDASLADQIEAGAIGFKIHEDWGTTPSAINHALDVADK  YDVQVAIHTDTLNEAGCVEDTMAAIAGRTMHTFHTEGAGGGHAPDIIKVAGEHNILPASTNPTIPFTVNTEAEHMDMLMV  CHHLDKSIKEDVQFADSRIRPQTIAAEDTLHDMGIFSITSSDSQAMGRVGEVITRTWQTADKNKKEFGRLKEEKGDNDNF  RIKRYLSKYTINPAIAHGISEYVGSVEVGKVADLVLWSPAFFGVKPNMIIKGGFIALSQMGDANASIPTPQPVYYREMFA  HHGKAKYDANITFVSQAAYDKGIKEELGLERQVLPVKNCRNITKKDMQFNDTTAHIEVNPETYHVFVDGKEVTSKPANKV  SLAQLFSIF | 569 |
| 48 | M3PPX9 | MKKISRKEYVSMYGPTTGDKVRLGDTDLIAEVEHDYTIYGEELKFGGGKTLREGMSQSNNPSKEELDLIITNALIVDYTGIYKADIGIKDGKIAGIGKGGNKDMQDGVKNNLSVGPATEALAGEGLIVTAGGIDTHIHFISPQQIPTAFASGVTTMIGGGTGPADGTNATTITPGRRNLKWMLRAAEEYSMNLGFLAKGNASNDASLADQIEAGAIGFKIHEDWGTTPSAINHALDVADKYDVQVAIHTDTLNEAGCVEDTMAAIAGRTMHTFHTEGAGGGHAPDIIKVAGEHNILPASTNPTIPFTVNTEAEHMDMLMVCHHLDKSIKEDVQFADSRIRPQTIAAEDTLHDMGIFSITSSDSQAMGRVGEVITRTWQTADKNKKEFGRLKEEKGDNDNFRIKRYLSKYTINPAIAHGISEYVGSVEVGKVADLVLWSPAFFGVKPNMIIKGGFIALSQMGDANASIPTPQPVYYREMFAHHGKAKYDANITFVSQAAYDKGIKEELGLERQVLPVKNCRNITKKDMQFNDTTAHIEVNPETYHVFVDGKEVTSKPANKVSLAQLFSIF | 569 |
| 49 | M3RFH4 | MKKISRKEYVSMYGPTTGDKVRLGDTDLIAEVEHDYTIYGEELKFGGGKTLREGMSQSNNPSKEELDLIITNALIVDYTGIYKADIGIKDGKIAGIGKGGNKDMQDGVKNNLSVGPATEALAGEGLIVTAGGIDTHIHFISPQQIPTAFASGVTTMIGGGTGPADGTNATTITPGRRNLKWMLRAAEEYSMNLGFLAKGNASNDASLADQIEAGAIGFKIHEDWGTTPSAINHALDVADKYDVQVAIHTDTLNEAGCVEDTMAAIAGRTMHTFHTEGAGGGHAPDIIKVAGEHNILPASTNPTIPFTVNTEAEHMDMLMVCHHLDKSIKEDVQFADSRIRPQTIAAEDTLHDMGIFSITSSDSQAMGRVGEVITRTWQTADKNKKEFGRLKEEKGDNDNFRIKRYLSKYTINPAIAHGISEYVGSVEVGKVADLVLWSPAFFGVKPNMIIKGGFIALSQMGDANASIPTPQPVYYREMFAHHGKAKYDANITFVSQAAYDKGIKEELGLERQVLPVKNCRNITKKDMQFNDTTAHIEVNPETYHVFVDGKEVTSKPANKVSLAQLFSIF | 569 |
| 50 | M3RWC6 | MKKISRKEYVSMYGPTTGDKVRLGDTDLIAEVEHDYTIYGEELKFGGGKTLREGMSQSNNPSKEELDLIITNALIVDYTGIYKADIGIKDGKIAGIGKGGNKDMQDGVKNNLSVGPATEALAGEGLIVTAGGIDTHIHFISPQQIPTAFASGVTTMIGGGTGPADGTNATTITPGRRNLKWMLRAAEEYSMNLGFLAKGNTSNDASLADQIEAGAIGFKIHEDWGTTPSAINHALDVADKYDVQVAIHTDTLNEAGCVEDTMAAIAGRTMHTFHTEGAGGGHAPDIIKVAGEHNILPASTNPTIPFTVNTEAEHMDMLMVCHHLDKSIKEDVQFADSRIRPQTIAAEDTLHDMGIFSITSSDSQAMGRVGEVITRTWQTADKNKKEFGRLKEEKGDNDNFRIKRYLSKYTINPAIAHGISEYVGSVEVGKVADLVLWSPAFFGVKPNMIIKGGFIALSQMGDANASIPTPQPVYYREMFAHHGKAKYDANITFVSQVAYDKGIKEELGLERQVLPVKNCRNITKKDMQFNDTTAHIEVNPETYHVFVDGKEVTSKPANKVSLAQLFSIF | 569 |
| 51 | M3TTE2 | MKKISRKEYVSMYGPTTGDKVRLGDTDLIAEVEHDYTIYGEELKFGGGKTLREGMSQSNNPSKEELDLIITNALIVDYTGIYKADIGIKDGKIAGIGKGGNKDMQDGVKNNLSVGPATEALAGEGLIVTAGGIDTHIHFISPQQIPTAFASGVTTMIGGGTGPADGTNATTITPGRRNLKWMLRAAEEYSMNLGFLAKGNTSNDASLADQIEAGAIGFKIHEDWGTTPSAINHALDVADKYDVQVAIHTDTLNEAGCVEDTMAAIAGRTMHTFHTEGAGGGHAPDIIKVAGEHNILPASTNPTIPFTVNTEAEHMDMLMVCHHLDKSIKEDVQFADSRIRPQTIAAEDTLHDMGIFSITSSDSQAMGRVGEVITRTWQTADKNKKEFGRLKEEKGDNDNFRIKRYLSKYTINPAIAHGISEYVGSVEVGKVADLVLWSPAFFGVKPNMIIKGGFIALSQMGDANASIPTPQPVYYREMFAHHGKAKYDANITFVSQAAYDKGIKEELGLERQVLPVKNCRNITKKDMQFNDTTAHIEVNPETYHVFVDGKEVTSKPANKVSLAQLFSIF | 569 |
| 52 | T0DA43 | MKKISRKEYVSMYGPTTGDKVRLGDTDLIAEVEHDYTIYGEELKFGGGKTLREGMSQSNNPSKEELDLIITNALIVDYTGIYKADIGIKDGKIAGIGKGGNKDMQDGVKNNLSVGPATEALAGEGLIVTAGGIDTHIHFISPQQIPTAFASGVTTMIGGGTGPADGTNATTITPGRRNLKWMLRAAEEYSMNLGFLAKGNTSNDASLADQIEAGAIGFKIHEDWGTTPSAINHALDVADKYDVQVAIHTDTLNEAGCVEDTMAAIAGRTMHTFHTEGAGGGHAPDIIKVAGEHNILPASTNPTIPFTVNTEAEHMDMLMVCHHLDKSIKEDVQFADSRIRPQTIAAEDTLHDMGIFSITSSDSQAMGRVGEVITRTWQTADKNKKEFGRLKEEKGDNDNFRIKRYLSKYTINPAIAHGISEYVGSVEVGKVADLVLWSPAFFGVKPNMIIKGGFIALSQMGDANASIPTPQPVYYREMFAHHGKAKYDANITFVSKAAYDKGIKEELGLERQVLPVKNCRNITKKDMQFNDTTAHIEVNPETYHVFVDGKEVTSKPATKVSLAQLFSIF | 569 |
| 53 | A0A060PQA0 | MKKISRKEYVSMYGPTTGDKVRLGDTDLIAEVEHDYTIYGEELKFGGGKTLREGMSQSNNPSKEELDLIITNALIVDYTGIYKADIGIKDGKIAGIGKGGNKDMQDGVKNNLSVGPATEALAGEGLIVTAGGIDTHIHFISPQQIPTAFASGVTTMIGGGTGPADGTNATTITPGRRNLKWMLRAAEEYSMNLGFLAKGNASNDASLADQIEAGAIGFKIHEDWGTTPSAINHALDVADKYDVQVAIHTDTLNEAGCVEDTMAAIAGRTMHTFHTEGAGGGHAPDIIKVAGEHNILPASTNPTIPFTVNTEAEHMDMLMVCHHLDKSIKEDVQFADSRIRPQTIAAEDTLHDMGIFSITSSDSQAMGRVGEVITRTWQTADKNKKEFGRLKEEKGDNDNFRIKRYLSKYTINPAIAHGISEYVGSVEVGKVADLVLWSPAFFGVKPNMIIKGGFIALSQMGDANASIPTPQPVYYREMFAHHGKAKYDANITFVSQAAYDKGIKEELGLERQVLPVKNCRNITKKDMQFNDTTAHIEVNPETYHVFVDGKEVTSKPANKVSLAQLFSIF | 569 |
| 54 | A0A0E0W9F6 | MKKISRKEYASMYGPTTGDKVRLGDTDLIAEVEHDYTIYGEELKFGGGKTLREGMSQSNNPSKEELDLIITNALIVDYTGIYKADIGIKDGKIAGIGKGGNKDTQDGVKNNLSVGPATEALAGEGLIVTAGGIDTHIHFISPQQIPTAFASGVTTMIGGGTGPADGTNATTITPGRRNLKFMLRAAEEYSVNLGFLAKGNASNDASLADQIEAGAIGLKIHEDWGTTPSAINHALDVADK  YDVQVAIHTDTLNEAGCVEDTMAAIAGRTMHTYHTEGAGGGHAPDIIKVAGEHNILPASTNPTIPFTVNTEAEHMDMLMVCHHLDKNIKEDVQFADSRIRPQTIAAEDTLHDMGIFSITSSDSQAMGRVGEVITRTWQTADKNKKEFGRLKEEKGDNDNFRIKRYLSKYTINPAIAHGISEYVGSVEVGKVADLVLWSPAFFGVKPNMIIKGGFIALSQMGDANASIPTPQPVYYREMFAHHGKAKYDANITFVSQAAYDKGIKEELGLERQVLPVKNCRNITKKDMQFNDTTAHIEVNSETYHVFVDGKEVTSKPANKVSLAQLFSIF | 569 |
| 55 | A0A6M5U692 | MKKISRKEYVSMYGPTTGDKVRLGDTDLIAEVEHDYTIYGEELKFGGGKTLREGMSQSNNPSKEELDLIITNALIVDYTGIYKADIGIKDGKIAGIGKGGNKDMQDGVKNNLSVGPATEALAGEGLIVTAGGIDTHIHFISPQQIPTAFASGVTTMIGGGTGPADGTNATTITPGRRNLKWMLRAAEEYSMNLGFLAKGNASNDASLADQIEAGAIGFKIHEDWGTTPSAINHALDVADKYDVQVAIHTDTLNEAGCVEDTMAAIAGRTMHTFHTEGAGGGHAPDIIKVAGEHNILPASTNPTIPFTVNTEAEHMDMLMVCHHLDKSIKEDVQFADSRIRPQTIAAEDTLHDMGIFSITSSDSQAMGRVGEVITRTWQTADKNKKEFGRLKEEKGDNDNFRIKRYLSKYTINPAIAHGISEYVGSVEVGKVADLVLWSPAFFGVKPNMIIKGGFIALSQMGDANASIPTPQPVYYREMFAHHGKAKYDANITFVSQAAYDKGIKEELGLERQVLPVKNCRNITKKDMQFNDTTAHIEVNPETYHVFVDGKEVTSKPANKVSLAQLFSIF | 569 |
| 56 | K7Y404 | MKKISRKEYVSMYGPTTGDKVRLGDTDLIAEVEHDYTIYGEELKFGGGKTLREGMSQSNNPSKEELDLIITNALIVDYTGIYKADIGIKDGKIAGIGKGGNKDMQDGVKNNLSVGPATEALAGEGLIVTAGGIDTHIHFISPQQIPTAFASGVTTMIGGGTGPADGTNATTITPGRRNLKWMLRAAEEYSMNLGFLAKGNASNDASLADQIEAGAIGFKIHEDWGTTPSAINHALDVADKYDVQVAIHTDTLNEAGCVEDTMAAIAGRTMHTFHTEGAGGGHAPDIIKVAGEHNILPASTNPTIPFTVNTEAEHMDMLMVCHHLDKSIKEDVQFADSRIRPQTIAAEDTLHDMGIFSITSSDSQAMGRVGEVITRTWQTADKNKKEFGRLKEEKGDNDNFRIKRYLSKYTINPAIAHGISEYVGSVEVGKVADLVLWSPAFFGVKPNMIIKGGFIALSQMGDANASIPTPQPVYYREMFAHHGKAKYDANITFVSQAAYDKGIKEELGLERQVLPVKNCRNITKKDMQFNDTTAHIEVNPETYHVFVDGKEVTSKPANKVSLAQLFSIF | 569 |
| 57 | K8GRK4 | MKKISRKEYVSMYGPTTGDKVRLGDTDLIAEVEHDYTIYGEELKFGGGKTLREGMSQSNNPSKEELDLIITNALIVDYTGIYKADIGIKDGKIAGIGKGGNKDMQDGVKNNLSVGPATEALAGEGLIVTAGGIDTHIHFISPQQIPTAFASGVTTMIGGGTGPADGTNATTITPGRRNLKWMLRAAEEYSMNLGFLAKGNTSNDASLADQIEAGAIGFKIHEDWGTTPSAINHALDVADKYDVQVAIHTDTLNEAGCVEDTMAAIAGRTMHTFHTEGAGGGHAPDIIKVAGEHNILPASTNPTIPFTVNTEAEHMDMLMVCHHLDKSIKEDVQFADSRIRPQTIAAEDTLHDMGIFSITSSDSQAMGRVGEVITRTWQTADKNKKEFGRLKEEKGDNDNFRIKRYLSKYTINPAIAHGISEYVGSVEVGKVADLVLWSPAFFGVKPNMIIKGGFIALSQMGDANASIPTPQPVYYREMFAHHGKAKYDANITFVSQAAYDKGIKEELGLERQVLPVKNCRNITKKDMQFNDTTAHIEVNPETYHVFVDGKEVTSKPANKVSLAQLFSIF | 569 |
| 58 | M3LB71 | MKKISRKEYVSMYGPTTGDKVRLGDTDLIAEVEHDYTIYGEELKFGGGKTLREGMSQSNNPSKEELDLIITNALIVDYTGIYKADIGIKDGKIAGIGKGGNKDMQDGVKNNLSVGPATEALAGEGLIVTAGGIDTHIHFISPQQIPTAFASGVTTMIGGGTGPADGTNATTITPGRRNLKWMLRAAEEYSMNLGFLAKGNASNDASLADQIEAGAIGFKIHEDWGTTPSAINHALDVADKYDVQVAIHTDTLNEAGCVEDTMAAIAGRTMHTFHTEGAGGGHAPDIIKVAGEHNILPASTNPTIPFTVNTEAEHMDMLMVCHHLDKSIKEDVQFADSRIRPQTIAAEDTLHDMGIFSITSSDSQAMGRVGEVITRTWQTADKNKKEFGRLKEEKGDNDNFRIKRYLSKYTINPAIAHGISEYVGSVEVGKVADLVLWSPAFFGVKPNMIIKGGFIALSQMGDANASIPTPQPVYYREMFAHHGKAKYDANITFVSQAAYDKGIKEELGLERQVLPVKNCRNITKKDMQFNDTTAHIEVNPETYHVFVDGKEVTSKPANKVSLAQLFSIF | 569 |
| 59 | M3LEW1 | MKKISRKEYVSMYGPTTGDKVRLGDTDLIAEVEHDYTIYGEELKFGGGKTLREGMSQSNNPSKEELDLIITNALIVDYTGIYKADIGIKDGKIAGIGKGGNKDMQDGVKNNLSVGPATEALAGEGLIVTAGGIDTHIHFISPQQIPTAFASGVTTMIGGGTGPADGTNATTITPGRRNLKWMLRAAEEYSMNLGFLAKGNASNDASLADQIEAGAIGFKIHEDWGTTPSAINHALDVADKYDVQVAIHTDTLNEAGCVEDTMAAIAGRTMHTFHTEGAGGGHAPDIIKVAGEHNILPASTNPTIPFTVNTEAEHMDMLMVCHHLDKSIKEDVQFADSRIRPQTIAAEDTLHDMGIFSITSSDSQAMGRVGEVITRTWQTADKNKKEFGRLKEEKGDNDNFRIKRYLSKYTINPAIAHGISEYVGSVEVGKVADLVLWSPAFFGVKPNMIIKGGFIALSQMGDANASIPTPQPVYYREMFAHHGKAKYDANITFVSQAAYDKGIKEELGLERQVLPVKNCRNITKKDMQFNDTTAHIEVNPETYHVFVDGKEVTSKPANKVSLAQLFSIF | 569 |
| 60 | M3LK16 | MKKISRKEYVSMYGPTTGDKVRLGDTDLIAEVEHDYTIYGEELKFGGGKTLREGMSQSNNPSKEELDLIITNALIVDYTGIYKADIGIKDGKIAGIGKGGNKDMQDGVKNNLSVGPATEALAGEGLIVTAGGIDTHIHFISPQQIPTAFASGVTTMIGGGTGPADGTNATTITPGRRNLKWMLRAAEEYSMNLGFLAKGNTSNDASLADQIEAGAIGFKIHEDWGTTPSAINHALDVADKYDVQVAIHTDTLNEAGCVEDTMAAIAGRTMHTFHTEGAGGGHAPDIIKVAGEHNILPASTNPTIPFTVNTEAEHMDMLMVCHHLDKSIKEDVQFADSRIRPQTIAAEDTLHDMGIFSITSSDSQAMGRVGEVITRTWQTADKNKKEFGRLKEEKGDNDNFRIKRYLSKYTINPAIAHGISEYVGSVEVGKVADLVLWSPAFFGVKPNMIIKGGFIALSQMGDANASIPTPQPVYYREMFAHHGKAKYDANITFVSQAAYDKGIKEELGLERQVLPVKNCRNITKKDMQFNDTTAHIEVNPETYHVFVDGKEVTSKPANKVSLAQLFSIF | 569 |
| 61 | M3ME32 | MKKISRKEYVSMYGPTTGDKVRLGDTDLIAEVEHDYTIYGEELKFGGGKTLREGMSQSNNPSKEELDLIITNALIVDYTGIYKADIGIKDGKIAGIGKGGNKDMQDGVKNNLSVGPATEALAGEGLIVTAGGIDTHIHFISPQQIPTAFASGVTTMIGGGTGPADGTNATTITPGRRNLKWMLRAAEEYSMNLGFLAKGNTSNDASLADQIEAGAIGFKIHEDWGTTPSAINHALDVADKYDVQVAIHTDTLNEAGCVEDTMAAIAGRTMHTFHTEGAGGGHAPDIIKVAGEHNILPASTNPTIPFTVNTEAEHMDMLMVCHHLDKSIKEDVQFADSRIRPQTIAAEDTLHDMGIFSITSSDSQAMGRVGEVITRTWQTADKNKKEFGRLKEEKGDNDNFRIKRYLSKYTINPAIAHGISEYVGSVEVGKVADLVLWSPAFFGVKPNMIIKGGFIALSQMGDANASIPTPQPVYYREMFAHHGKAKYDANITFVSQAAYDKGIKEELGLERQVLPVKNCRNITKKDMQFNDTTAHIEVNPETYHVFVDGKEVTSKPANKVSLAQLFSIF | 569 |
| 62 | M3NM20 | MKKISRKEYVSMYGPTTGDKVRLGDTDLIAEVEHDYTIYGEELKFGGGKTLREGMSQSNNPSKEELDLIITNALIVDYTGIYKADIGIKDGKIAGIGKGGNKDMQDGVKNNLSVGPATEALAGEGLIVTAGGIDTHIHFISPQQIPTAFASGVTTMIGGGTGPADGTNATTITPGRRNLKWMLRAAEEYSMNLGFLAKGNTSNDASLADQIEAGAIGFKIHEDWGTTPSAINHALDVADKYDVQVAIHTDTLNEAGCVEDTMAAIAGRTMHTFHTEGAGGGHAPDIIKVAGEHNILPASTNPTIPFTVNTEAEHMDMLMVCHHLDKSIKEDVQFADSRIRPQTIAAEDTLHDMGIFSITSSDSQAMGRVGEVITRTWQTADKNKKEFGRLKEEKGDNDNFRIKRYLSKYTINPAIAHGISEYVGSVEVGKVADLVLWSPAFFGVKPNMIIKGGFIALSQMGDANASIPTPQPVYYREMFAHHGKAKYDANITFVSQAAYDKGIKEELGLERQVLPVKNCRNITKKDMQFNDTTAHIEVNPETYHVFVDGKEVTSKPANKVSLAQLFSIF | 569 |
| 63 | M3RQH5 | MKKISRKEYVSMYGPTTGDKVRLGDTDLIAEVEHDYTIYGEELKFGGGKTLREGMSQSNNPSKEELDLIITNALIVDYTGIYKADIGIKDGKIAGIGKGGNKDMQDGVKNNLSVGPATEALAGEGLIVTAGGIDTHIHFISPQQIPTAFASGVTTMIGGGTGPADGTNATTITPGRRNLKWMLRAAEEYSMNLGFLAKGNTSNDASLADQIEAGAIGFKIHEDWGTTPSAINHALDVADKYDVQVAIHTDTLNEAGCVEDTMAAIAGRTMHTFHTEGAGGGHAPDIIKVAGEHNILPASTNPTIPFTVNTEAEHMDMLMVCHHLDKSIKEDVQFADSRIRPQTIAAEDTLHDMGIFSITSSDSQAMGRVGEVITRTWQTADKNKKEFGRLKEEKGDNDNFRIKRYLSKYTINPAIAHGISEYVGSVEVGKVADLVLWSPAFFGVKPNMIIKGGFIALSQMGDANASIPTPQPVYYREMFAHHGKAKYDANITFVSQAAYDKGIKEELGLERQVLPVKNCRNITKKDMQFNDTTAHIEVNPETYHVFVDGKEVTSKPANKVSLAQLFSIF | 569 |
| 64 | M3TI66 | MKKISRKEYVSMYGPTTGDKVRLGDTDLIAEVEHDYTIYGEELKFGGGKTLREGMSQSNNPSKEELDLIITNALIVDYTGIYKADIGIKDGKIAGIGKGGNKDMQDGVKNNLSVGPATEALAGEGLIVTAGGIDTHIHFISPQQIPTAFASGVTTMIGGGTGPADGTNATTITPGRRNLKWMLRAAEEYSMNLGFLAKGNASNDASLADQIEAGAIGFKIHEDWGTTPSAINHALDVADKYDVQVAIHTDTLNEAGCVEDTMAAIAGRTMHTFHTEGAGGGHAPDIIKVAGEHNILPASTNPTIPFTVNTEAEHMDMLMVCHHLDKSIKEDVQFADSRIRPQTIAAEDTLHDMGIFSITSSDSQAMGRVGEVITRTWQTADKNKKEFGRLKEEKGDNDNFRIKRYLSKYTINPAIAHGISEYVGSVEVGKVADLVLWSPAFFGVKPNMIIKGGFIALSQMGDANASIPTPQPVYYREMFAHHGKAKYDANITFVSQAAYDKGIKEELGLERQVLPVKNCRNITKKDMQFNDTTAHIEVNPETYHVFVDGKEVTSKPANKVSLAQLFSIF | 569 |
| 65 | M5YAQ5 | MKKISRKEYVSMYGPTTGDKVRLGDTDLIAEVEHDYTIYGEELKFGGGKTLREGMSQSNNPSKEELDLIITNALIVDYTGIYKADIGIKDGKIAGIGKGGNKDMQDGVKNNLSVGPATEALAGEGLIVTAGGIDTHIHFISPQQIPTAFASGVTTMIGGGTGPADGTNATTITPGRRNLKWMLRAAEEYSMNLGFLAKGNASNDASLADQIEAGAIGFKIHEDWGTTPSAINHALDVADKYDVQVAIHTDTLNEAGCVEDTMAAIAGRTMHTFHTEGAGGGHAPDIIKVAGEHNILPASTNPTIPFTVNTEAEHMDMLMVCHHLDKSIKEDVQFADSRIRPQTIAAEDTLHDMGIFSITSSDSQAMGRVGEVITRTWQTADKNKKEFGRLKEEKGDNDNFRIKRYLSKYTINPAIAHGISEYVGSVEVGKVADLVLWSPAFFGVKPNMIIKGGFIALSQMGDANASIPTPQPVYYREMFAHHGKAKYDANITFVSQAAYDKGIKEELGLERQVLPVKNCRNITKKDMQFNDTTAHIEVNPETYHVFVDGKEVTSKPANKVSLAQLFSIF | 569 |
| 66 | M5YL41 | MKKISRKEYVSMYGPTTGDKVRLGDTDLIAEVEHDYTIYGEELKFGGGKTLREGMSQSNNPSKEELDLIITNALIVDYTGIYKADIGIKDGKIAGIGKGGNKDMQDGVKNNLSVGPATEALAGEGLIVTAGGIDTHIHFISPQQIPTAFASGVTTMIGGGTGPADGTNATTITPGRRNLKWMLRAAEEYSMNLGFLAKGNTSNDASLADQIEAGAIGFKIHEDWGTTPSAINHALDVADKYDVQVAIHTDTLNEAGCVEDTMAAIAGRTMHTFHTEGAGGGHAPDIIKVAGEHNILPASTNPTIPFTVNTEAEHMDMLMVCHHLDKSIKEDVQFADSRIRPQTIAAEDTLHDMGIFSITSSDSQAMGRVGEVITRTWQTADKNKKEFGRLKEEKGDNDNFRIKRYLSKYTINPAIAHGISEYVGSVEVGKVADLVLWSPAFFGVKPNMIIKGGFIALSQMGDANASIPTPQPVYYREMFAHHGKAKYDANITFVSQAAYDKGIKEELGLERQVLPVKNCRNITKKDMQFNDTTAHIEVNPETYHVFVDGKEVTSKPANKVSLAQLFSIF | 569 |
| 67 | T2SPM2 | MIGGGTGPADGTNATTITPGRRNLKWMLRAAEEYSMNLGFLAKGNASNDASLADQIEAGAIGFKIHEDWGTTPSAINHALDVADKYDVQVAIHTDTLNEAGCVEDTMAAIAGRTMHTFHTEGAGGGHAPDIIKVAGEHNILPASTNPTIPFTVNTEAEHMDMLMVCHHLDKSIKEDVQFADSRIRPQTIAAEDTLHDMGIFSITSSDSQAMGRVGEVITRTWQTADKNKKEFGRLKEEKGDNDNFRIKRYLSKYTINPAIAHGISEYVGSVEVGKVADLVLWSPAFFGVKPNMIIKGGFIALSQMGDANASIPTPQPVYYREMFAHHGKAKYDANITFVSQAAYDKGIKEELGLERQVLPVKNCRNITKKDMQFNDTTAHIEVNPETYHVFVDGKEVTSKPANKVSLAQLFSIF | 414 |
| 68 | C1PIF5 | MKKISRKEYVSMYGPTTGDKVRLGDTDLILEVEHDCTTYGEEIKFGGGKTIRDGMGQTNSPSSHELDLVITNALIVDYTGIYKADIGIKDGKIHGIGKAGNKDIQDGVCNRLCVGPATEALAGEGLIVTAGGIDTHIHFISPQQIPTAFASGITTMLGGGTGPADGTNATTITPGRWNLKEMLRASEEYAMNLGYMGKGNVSYEPSLVEQLEAGAIGFKIHEDWGSTPSAIHHALKIADE  YDVQVAIHTDTLNEAGCVEDTLEAIAGRTIHTFHTEGAGGGHAPDVIKMAGAFNVLPASTNPTIPFTKNTEAEHMDMLMVCHHLDKNIKEDVEFAD | 336 |
| 69 | A0A3Q8APR6 | MKKISRKEYVSMYGPTTGDKVRLGDTDLIAEVEHDYTIYGEELKFGGGKTLREGMSQSNNPSKEELDLIITNALIVDYTGIYKADIGIKDGKIAGIGKGGNKDMQDGVKNNLSVGPATEALAGEGLIVTAGGIDTHIHFISPQQIPTAFASGVTTMIGGGTGPADGTNATTITPGRRNLKWMLRAAEEYSMNLGFLAKGNASNDASLADQIEAGAIGFKIHEDWGTTPSAINHALDVADKYDVQVAIHTDTLNEAGCVEDTMAAIAGRTMHTFHTEGAGGGHAPDIIKVAGEHNILPASTNPTIPFTVNTEAEHMDMLMVCHHLDKSIKEDVQFADSRIRPQTIAAEDTLHDMGIFSITSSDSQAMGRVGEVITRTWQTADKNKKEFGRLKEEKGDNDNFRIKRYLSKYTINPAIAHGISEYVGSVEVGKVADLVLWSPAFFGVKPNMIIKGGFIALSQMGDANASIPTPQPVYYREMFAHHGKAKYDANITFVSQAAYDKGIKEELGLERQVLPVKNCRNITKKDMQFNDTTAHIEVNPETYHVFVDGKEVTSKPANKVSLAQLFSIF | 569 |
| 70 | G2M2S6 | MKKISRKEYASMYGPTTGDKVRLGDTDLIAEVEHDYTIYGEELKFGGGKTLREGMSQSNNPSKEELDLIITNALIVDYTGIYKADIGIKDGKIAGIGKGGNKDMQDGVKNNLSVGPATEALAGEGLIVTAGGIDTHIHFISPQQIPTAFASGVTTMIGGGTGPADGTNATTITPGRRNLKWMLRAAEEYSMNLGFLAKGNASNDASLADQIEAGAIGFKIHEDWGTTPSAINHALDVADKYDVQVAIHTDTLNEAGCVEDTMAAIAGRTMHTFHTEGAGGGHAPDIIKVAGEHNILPASTNPTIPFTVNTEAEHMDMLMVCHHLDKSIKEDVQFADSRIRPQTIAAEDTLHDMGIFSITSSDSQAMGRVGEVITRTWQTADKNKKEFGRLKEEKGDNDNFRIKRYLSKYTINPAIAHGISEYVGSVEVGKVADLVLWSPAFFGVKPNMIIKGGFIALSQMGDANASIPTPQPVYYREMFAHHGKAKYDANITFVSQAAYDKGIKEELGLERQVLPVKNCRNITKKDMQFNDTTAHIEVNPETYHVFVDGKEVTSKPANKVSLAQLFSIF | 569 |
| 71 | K7YLU6 | MKKISRKEYASMYGPTTGDKVRLGDTDLIAEVEHDYTIYGEELKFGGGKTLREGMSQSNNPSKEELDLIITNALIVDYTGIYKADIGIKDGKIAGIGKGGNKDMQDGVKNNLSVGPATEALAGEGLIVTAGGIDTHIHFISPQQIPTAFASGVTTMIGGGTGPADGTNATTITPGRRNLKWMLRAAEEYSMNLGFLGKGNASNDASLADQIEAGAIGFKIHEDWGTTPSAINHALDVADKYDVQVAIHTDTLNEAGCVEDTMAAIAGRTMHTFHTEGAGGGHAPDIIKVAGEHNILPASTNPTIPFTVNTEAEHMDMLMVCHHLDKSIKEDVQFADSRIRPQTIAAEDTLHDMGIFSITSSDSQAMGRVGEVITRTWQTADKNKKEFGRLKEEKGDNDNFRIKRYLSKYTINPAIAHGISEYVGSVEVGKVADLVLWSPAFFGVKPNMIIKGGFIALSQMGDANASIPTPQPVYYREMFAHHGKAKYDANITFVSQAAYNKGIKEELGLERQVLPVKNCRNITKKDMQFNDTTAHIEVNPETYHVFVDGKEVTSKPANKVSLAQLFSIF | 569 |
| 72 | K7YPA3 | MKMKKLDYVNTYGPTKGDKVRLGDTEIWAEVEHDYTIYGEELKFGAGKTIREGMGQSNSHDENTLDLVITNALIIDYTGIYKADIGIKNGKIAGIGKAGNKDMQDGVSPNLVVGVGTEALAGEGMIVTAGGIDSHTHFLSPQQFPTALANGVTTMFGGGTGPVDGTNATTITPGEWNIHRMLRAAEEYAMNVGFLGKGNSSSKTQLVEQIEAGVVGFKLHEDWGTTPSAIDTCLSVADEYDVQVCIHTDTVNEAGYVEDTLNAMNGRAIHAYHIEGAGGGHSPDVITMAGEENILPSSTTPTIPYTINTVAEHLDMLMTCHHLDKKIREDLQFSQSRIRPGSIAAEDVLHDNGMIAMTSSDSQAMGRAGEVVPRTWQTADKNKKEFGPLKEDAQNGNDNFRIKRYISKYTINPAITHGVSEYIGSVEAGKIADLVVWNPAFFGVKPKIIIKGGLVVFSEMGDSNASVPTPQPVYYREMFGHHGKAKFDTSITFVNKLAYEKGIKEKLGLERQVLPIKNVRNITKKDFKFNNTTGKLTVDPKTFEVFLDGKLCTSKPASELPLAQRYTFF | 569 |
| 73 | M3LJJ0 | MKKISRKEYVSMYGPTTGDKVRLGDTDLIAEVEHDYTIYGEELKFGGGKTLREGMSQSNNPSKEELDLIITNALIVDYTGIYKADIGIKDGKIAGIGKGGNKDMQDGVKNNLSVGPATEALAGEGLIVTAGGIDTHIHFISPQQIPTAFASGVTTMIGGGTGPADGTNATTITPGRRNLKWMLRAAEEYSMNLGFLAKGNTSNDASLADQIEAGAIGFKIHEDWGTTPSAINHALDVADKYDVQVAIHTDTLNEAGCVEDTMAAIAGRTMHTFHTEGAGGGHAPDIIKVAGEHNILPASTNPTIPFTVNTEAEHMDMLMVCHHLDKSIKEDVQFADSRIRPQTIAAEDTLHDMGIFSITSSDSQAMGRVGEVITRTWQTADKNKKEFGRLKEEKGDNDNFRIKRYLSKYTINPAIAHGISEYVGSVEVGKVADLVLWSPAFFGVKPNMIIKGGFIALSQMGDANASIPTPQPVYYREMFAHHGKAKYDANITFVSQAAYDKGIKEELGLERQVLPVKNCRNITKKDMQFNDTTAHIEVNPETYHVFVDGKEVTSKPANKVSLAQLFSIF | 569 |
| 74 | M3LY90 | MKKISRKEYVSMYGPTTGDKVRLGDTDLIAEVEHDYTIYGEELKFGGGKTLREGMSQSNNPSKEELDLIITNALIVDYTGIYKADIGIKDGKIAGIGKGGNKDMQDGVKNNLSVGPATEALAGEGLIVTAGGIDTHIHFISPQQIPTAFASGVTTMIGGGTGPADGTNATTITPGRRNLKWMLRAAEEYSMNLGFLAKGNTSNDASLADQIEAGAIGFKIHEDWGTTPSAINHALDVADKYDVQVAIHTDTLNEAGCVEDTMAAIAGRTMHTFHTEGAGGGHAPDIIKVAGEHNILPASTNPTIPFTVNTEAEHMDMLMVCHHLDKSIKEDVQFADSRIRPQTIAAEDTLHDMGIFSITSSDSQAMGRVGEVITRTWQTADKNKKEFGRLKEEKGDNDNFRIKRYLSKYTINPAIAHGISEYVGSVEVGKVADLVLWSPAFFGVKPNMIIKGGFIALSQMGDANASIPTPQPVYYREMFAHHGKAKYDANITFVSQAAYDKGIKEELGLERQVLPVKNCRNITKKDMQFNDTTAHIEVNPETYHVFVDGKEVTSKPANKVSLAQLFSIF | 569 |
| 75 | M3NFM7 | MKKISRKEYVSMYGPTTGDKVRLGiGAMDTDLIAEVEHDYTIYGEELKFGGGKTLREGMSQSNNPSKEELDLIITNALIVDYTGIYKADIGIKDGKIAGIGKGGNKDMQDGVKNNLSVGPATEALAGEGLIVTAGGIDTHIHFISPQQIPTAFASGVTTMIGGGTGPADGTNATTITPGRRNLKWMLRAAEEYSMNLGFLAKGNTSNDASLADQIEAGAIGFKIHEDWGTTPSAINHALDVADKYDVQVAIHTDTLNEAGCVEDTMAAIAGRTMHTFHTEGAGGGHAPDIIKVAGEHNILPASTNPTIPFTVNTEAEHMDMLMVCHHLDKSIKEDVQFADSRIRPQTIAAEDTLHDMGIFSITSSDSQAMGRVGEVITRTWQTADKNKKEFGRLKEEKGDNDNFRIKRYLSKYTINPAIAHGISEYVGSVEVGKVADLVLWSPAFFGVKPNMIIKGGFIALSQMGDANASIPTPQPVYYREMFAHHGKAKYDANITFVSQAAYDKGIKEELGLERQVLPVKNCRNITKKDMQFNDTTAHIEVNPETYHVFVDGKEVTSKPANKVSLAQLFSIF | 573 |
| 76 | M3NGY3 | MKKISRKEYVSMYGPTTGDKVRLGDTDLIAEVEHDYTIYGEELKFGGGKTLREGMSQSNNPSKEELDLIITNALIVDYTGIYKADIGIKDGKIAGIGKGGNKDMQDGVKNNLSVGPATEALAGEGLIVTAGGIDTHIHFISPQQIPTAFASGVTTMIGGGTGPADGTNATTITPGRRNLKWMLRAAEEYSMNLGFLAKGNASNDASLADQIEAGAIGFKIHEDWGTTPSAINHALDVADKYDVQVAIHTDTLNEAGCVEDTMAAIAGRTMHTFHTEGAGGGHAPDIIKVAGEHNILPASTNPTIPFTVNTEAEHMDMLMVCHHLDKSIKEDVQFADSRIRPQTIAAEDTLHDMGIFSITSSDSQAMGRVGEVITRTWQTADKNKKEFGRLKEEKGDNDNFRIKRYLSKYTINPAIAHGISEYVGSVEVGKVADLVLWSPAFFGVKPNMIIKGGFIALSQMGDANASIPTPQPVYYREMFAHHGKAKYDANITFVSQAAYDKGIKEELGLERQVLPVKNCRNITKKDMQFNDTTAHIEVNPETYHVFVDGKEVTSKPANKVSLAQLFSIF | 569 |
| 77 | M3NT03 | MKKISRKEYVSMYGPTTGDKVRLGDTDLIAEVEHDYTIYGEELKFGGGKTLREGMSQSNNPSKEELDLIITNALIVDYTGIYKADIGIKDGKIAGIGKGGNKDMQDGVKNNLSVGPATEALAGEGLIVTAGGIDTHIHFISPQQIPTAFASGVTTMIGGGTGPADGTNATTITPGRRNLKWMLRAAEEYSMNLGFLAKGNTSNDASLADQIEAGAIGFKIHEDWGTTPSAINHALDVADKYDVQVAIHTDTLNEAGCVEDTMAAIAGRTMHTFHTEGAGGGHAPDIIKVAGEHNILPASTNPTIPFTVNTEAEHMDMLMVCHHLDKSIKEDVQFADSRIRPQTIAAEDTLHDMGIFSITSSDSQAMGRVGEVITRTWQTADKNKKEFGRLKEEKGDNDNFRIKRYLSKYTINPAIAHGISEYVGSVEVGKVADLVLWSPAFFGVKPNMIIKGGFIALSQMGDANASIPTPQPVYYREMFAHHGKAKYDANITFVSQAAYDKGIKEELGLERQVLPVKNCRNITKKDMQFNDTTAHIEVNPETYHVFVDGKEVTSKPANKVSLAQLFSIF | 569 |
| 78 | M3P1K4 | MKKISRKEYVSMYGPTTGDKVRLGDTDLIAEVEHDYTIYGEELKFGGGKTLREGMSQSNNPSKEELDLIITNALIVDYTGIYKADIGIKDGKIAGIGKGGNKDMQDGVKNNLSVGPATEALAGEGLIVTAGGIDTHIHFISPQQIPTAFASGVTTMIGGGTGPADGTNATTITPGRRNLKWMLRAAEEYSMNLGFLAKGNASNDASLADQIEAGAIGFKIHEDWGTTPSAINHALDVADKYDVQVAIHTDTLNEAGCVEDTMAAIAGRTMHTFHTEGAGGGHAPDIIKVAGEHNILPASTNPTIPFTVNTEAEHMDMLMVCHHLDKSIKEDVQFADSRIRPQTIAAEDTLHDMGIFSITSSDSQAMGRVGEVITRTWQTADKNKKEFGRLKEEKGDNDNFRIKRYLSKYTINPAIAHGISEYVGSVEVGKVADLVLWSPAFFGVKPNMIIKGGFIALSQMGDANASIPTPQPVYYREMFAHHGKAKYDANITFVSQAAYDKGIKEELGLERQVLPVKNCRNITKKDMQFNDTTAHIEVNPETYHVFVDGKEVTSKPANKVSLAQLFSIF | 569 |
| 79 | M3SKK0 | MKKISRKEYVSMYGPTTGDKVRLGDTDLIAEVEHDYTIYGEELKFGGGKTLREGMSQSNNPSKEELDLIITNALIVDYTGIYKADIGIKDGKIAGIGKGGNKDMQDGVKNNLSVGPATEALAGEGLIVTAGGIDTHIHFISPQQIPTAFASGVTTMIGGGTGPADGTNATTITPGRRNLKWMLRAAEEYSMNLGFLAKGNASNDASLADQIEAGAIGFKIHEDWGTTPSAINHALDIADKYDVQVAIHTDTLNEAGCVEDTMAAIAGRTMHTFHTEGAGGGHAPDIIKVAGEHNILPASTNPTIPFTVNTEAEHMDMLMVCHHLDKSIKEDVQFADSRIRPQTIAAEDTLHDMGIFSITSSDSQAMGRVGEVITRTWQTADKNKKEFGRLKEEKGDNDNFRIKRYLSKYTINPAIAHGISEYVGSVEVGKVADLVLWSPAFFGVKPNMIIKGGFIALSQMGDANASIPTPQPVYYREMFAHHGKAKYDANITFVSQAAYDKGIKEELGLERQVLPVKNCRNITKKDMQFNDTTAHIEVNPETYHVFVDGKEVTSKPANKVSLAQLFSIF | 569 |
| 80 | M5YAP7 | MKKISRKEYVSMYGPTTGDKVRLGDTDLIAEVEHDYTIYGEELKFGGGKTLREGMSQSNNPSKEELDLIITNALIVDYTGIYKADIGIKDGKIAGIGKGGNKDMQDGVKNNLSVGPATEALAGEGLIVTAGGIDTHIHFISPQQIPTAFASGVTTMIGGGTGPADGTNATTITPGRRNLKWMLRAAEEYSMNLGFLAKGNASNDASLADQIEAGAIGFKIHEDWGTTPSAINHALDVADKYDVQVAIHTDTLNEAGCVEDTMAAIAGRTMHTFHTEGAGGGHAPDIIKVAGEHNILPASTNPTIPFTVNTEAEHMDMLMVCHHLDKSIKEDVQFADSRIRPQTIAAEDTLHDMGIFSITSSDSQAMGRVGEVITRTWQTADKNKKEFGRLKEEKGDNDNFRIKRYLSKYTINPAIAHGISEYVGSVEVGKVADLVLWSPAFFGVKPNMIIKGGFIALSQMGDANASIPTPQPVYYREMFAHHGKAKYDANITFVSQAAYDKGIKEELGLERQVLPVKNCRNITKKDMQFNDTTAHIEVNPETYHVFVDGKEVTSKPANKVSLAQLFSIF | 569 |
| 81 | T9WSM0 | MKKISRKEYVSMYGPTTGDKVRLGDTDLIAEVEHDYTIYGEELKFGGGKTLREGMSQSNNPSKEELDLIITNALIVDYTGIYKADIGIKDGKIAGIGKGGNKDMQDGVKNNLSVGPATEALAGEGLIVTAGGIDTHIHFISPQQIPTAFASGVTTMIGGGTGPADGTNATTITPGRRNLKWMLRAAEEYSMNLGFLAKGNTSNDASLADQIEAGAIGFKIHEDWGTTPSAINHALDVADKYDVQVAIHTDTLNEAGCVEDTMAAIAGRTMHTFHTEGAGGGHAPDIIKVAGEHNILPASTNPTIPFTVNTEAEHMDMLMVCHHLDKSIKEDVQFADSRIRPQTIAAEDTLHDMGIFSITSSDSQAMGRVGEVITRTWQTADKNKKEFGRLKEEKGDNDNFRIKRYLSKYTINPAIAHGISEYVGSVEVGKVADLVLWSPAFFGVKPNMIIKGGFIALSQMGDANASIPTPQPVYYREMFAHHGKAKYDANITFVSQVAYDKGIKEELGLERQVLPVKNCRNITKKDMQFNDTTAHIEVNPETYHVFVDGKEVTSKPANKVSLAQLFSIF | 569 |
| 82 | X2ICA2 | MKKISRKEYASMYGPTTGDKVRLGDTDLIAEVEHDYTIYGEELKFGGGKTLREGMSQSNNPSKEELDLIITNALIVDYTGIYKADIGIKDGKIAGIGKGGNKDMQDGVKNNLSVGPATEALAGEGLIVTAGGIDTHIHFISPQQIPTAFASGVTTMIGGGTGPADGTNATTITPGRRNLKWMLRAAEEYSMNLGFLAKGNTSNDASLADQIEAGAIGFKIHEDWGTTPSAINHALDVADKYDVQVAIHTDTLNEAGCVEDTMAAIAGRTMHTFHTEGAGGGHAPDIIKVAGEHNILPASTNPTIPFTVNTEAEHMDMLMVCHHLDKSIKEDVQFADSRIRPQTIAAEDTLHDMGIFSITSSDSQAMGRVGEVITRTWQTADKNKKEFGRLKEEKGDNDNFRIKRYLSKYTINPAIAHGISEYVGSVEVGKVADLVLWSPAFFGVKPNMIIKGGFIALSQMGDANASIPTPQPVYYREMFAHHGKAKYDANITFVSQAAYDKGIKEELGLERQVLPVKNCRNITKKDMQFNDTTAHIEVNPETYHVFVDGKEVTSKPANKVSLAQLFSIF | 569 |
| 83 | J0HXK2 | MKKISRKEYVSMYGPTTGDKVRLGDTDLIAEVEHDYTIYGEELKFGGGKTLREGMSQSNNPSKEELDLIITNALIVDYTGIYKADIGIKDGKIAGIGKGGNKDMQDGVKNNLSVGPATEALAGEGLIVTAGGIDTHIHFISPQQIPTAFASGVTTMIGGGTGPADGTNATTITPGRRNLKWMLRAAEEYSMNLGFLAKGNASNDASLADQIEAGAIGFKIHEDWGTTPSAINHALDVADKYDVQAAIPTDTLNEAGCVEDTTAAIAGRTMHTFHTEGAGGGHAPDIIKVAGEHNILPASTNPTIPFTVNTEAEHMDMLMVCHHLDKSIKEDVQFADSRIRPQTIAAEDTLHDMGIFSITSSDSQAMGRVGEVITRTWQTADKNKKEFGRLKEEKGDNDNFRIKRYLSKYTINPAIAHGISEYVGSVEVGKVADLVLWSPAFFGVKPNMIIKGGFIALSQMGDANASIPTPQPVYYREMFAHHGKAKYDANITFVSQAAYDKGIKEELGLERQVLPVKNCRNITKKDMQFNDTTAHIEVNPETYHVFVDGKEVTSKPATKVSLAQLFSIF | 569 |
| 84 | M3KAQ7 | MKKISRKEYVSMYGPTTGDKVRLGDTDLIAEVEHDYTIYGEELKFGGGKTLREGMSQSNNPSKEELDLIITNALIVDYTGIYKADIGIKDGKIAGIGKGGNKDMQDGVKNNLSVGPATEALAGEGLIVTAGGIDTHIHFISPQQIPTAFASGVTTMIGGGTGPADGTNATTITPGRRNLKWMLRAAEEYSMNLGFLAKGNTSNDASLADQIEAGAIGFKIHEDWGTTPSAINHALDVADKYDVQVAIHTDTLNEAGCVEDTMAAIAGRTMHTFHTEGAGGGHAPDIIKVAGEHNILPASTNPTIPFTVNTEAEHMDMLMVCHHLDKSIKEDVQFADSRIRPQTIAAEDTLHDMGIFSITSSDSQAMGRVGEVITRTWQTADKNKKEFGRLKEEKGDNDNFRIKRYLSKYTINPAIAHGISEYVGSVEVGKVADLVLWSPAFFGVKPNMIIKGGFIALSQMGDANASIPTPQPVYYREMFAHHGKAKYDANITFVSQAAYDKGIKEELGLERQVLPVKNCRNITKKDMQFNDTTAHIEVNPETYHVFVDGKEVTSKPANKVSLAQLFSIF | 569 |
| 85 | M3M4N4 | MKKISRKEYVSMYGPTTGDKVRLGDTDLIAEVEHDYTIYGEELKFGGGKTLREGMSQSNNPSKEELDLIITNALIVDYTGIYKADIGIKDGKIAGIGKGGNKDMQDGVKNNLSVGPATEALAGEGLIVTAGGIDTHIHFISPQQIPTAFASGVTTMIGGGTGPADGTNATTITPGRRNLKWMLRAAEEYSMNLGFLAKGNASNDASLADQIEAGAIGFKIHEDWGTTPSAINHALDVADKYDVQVAIHTDTLNEAGCVEDTMAAIAGRTMHTFHTEGAGGGHAPDIIKVAGEHNILPASTNPTIPFTVNTEAEHMDMLMVCHHLDKSIKEDVQFADSRIRPQTIAAEDTLHDMGIFSITSSDSQAMGRVGEVITRTWQTADKNKKEFGRLKEEKGDNDNFRIKRYLSKYTINPAIAHGISEYVGSVEVGKVADLVLWSPAFFGVKPNMIIKGGFIALSQMGDANASIPTPQPVYYREMFAHHGKAKYDANITFVSQAAYDKGIKEELGLERQVLPVKNCRNITKKDMQFNDTTAHIEVNPETYHVFVDGKEVTSKPANKVSLAQLFSIF | 569 |
| 86 | M3MK80 | MKKISRKEYVSMYGPTTGDKVRLGDTDLIAEVEHDYTIYGEELKFGGGKTLREGMSQSNNPSKEELDLIITNALIVDYTGIYKADIGIKDGKIAGIGKGGNKDMQDGVKNNLSVGPATEALAGEGLIVTAGGIDTHIHFISPQQIPTAFASGVTTMIGGGTGPADGTNATTITPGRRNLKWMLRAAEEYSMNLGFLAKGNASNDASLADQIEAGAIGFKIHEDWGTTPSAINHALDVADKYDVQVAIHTDTLNEAGCVEDTMAAIAGRTMHTFHTEGAGGGHAPDIIKVAGEHNILPASTNPTIPFTVNTEAEHMDMLMVCHHLDKSIKEDVQFADSRIRPQTIAAEDTLHDMGIFSITSSDSQAMGRVGEVITRTWQTADKNKKEFGRLKEEKGDNDNFRIKRYLSKYTINPAIAHGISEYVGSVEVGKVADLVLWSPAFFGVKPNMIIKGGFIALSQMGDANASIPTPQPVYYREMFAHHGKAKYDANITFVSQAAYDKGIKEELGLERQVLPVKNCRNITKKDMQFNDTTAHIEVNPETYHVFVDGKEVTSKPANKVSLAQLFSIF | 569 |
| 87 | M3MQU8 | MKKISRKEYVSMYGPTTGDKVRLGDTDLIAEVEHDYTIYGEELKFGGGKTLREGMSQSNNPSKEELDLIITNALIVDYTGIYKADIGIKDGKIAGIGKGGNKDMQDGVKNNLSVGPATEALAGEGLIVTAGGIDTHIHFISPQQIPTAFASGVTTMIGGGTGPADGTNATTITPGRRNLKWMLRAAEEYSMNLGFLAKGNTSNDASLADQIEAGAIGFKIHEDWGTTPSAINHALDVADKYDVQVAIHTDTLNEAGCVEDTMAAIAGRTMHTFHTEGAGGGHAPDIIKVAGEHNILPASTNPTIPFTVNTEAEHMDMLMVCHHLDKSIKEDVQFADSRIRPQTIAAEDTLHDMGIFSITSSDSQAMGRVGEVITRTWQTADKNKKEFGRLKEEKGDNDNFRIKRYLSKYTINPAIAHGISEYVGSVEVGKVADLVLWSPAFFGVKPNMIIKGGFIALSQMGDANASIPTPQPVYYREMFAHHGKAKYDANITFVSQAAYDKGIKEELGLERQVLPVKNCRNITKKDMQFNDTTAHIEVNPETYHVFVDGKEVTSKPANKVSLAQLFSIF | 569 |
| 88 | M3PTR1 | MKKISRKEYVSMYGPTTGDKVRLGDTDLIAEVEHDYTIYGEELKFGGGKTLREGMSQSNNPSKEELDLIITNALIVDYTGIYKADIGIKDGKIAGIGKGGNKDMQDGVKNNLSVGPATEALAGEGLIVTAGGIDTHIHFISPQQIPTAFASGVTTMIGGGTGPADGTNATTITPGRRNLKWMLRAAEEYSMNLGFLAKGNTSNDASLADQIEAGAIGFKIHEDWGTTPSAINHALDVADKYDVQVAIHTDTLNEAGCVEDTMAAIAGRTMHTFHTEGAGGGHAPDIIKVAGEHNILPASTNPTIPFTVNTEAEHMDMLMVCHHLDKSIKEDVQFADSRIRPQTIAAEDTLHDMGIFSITSSDSQAMGRVGEVITRTWQTADKNKKEFGRLKEEKGDNDNFRIKRYLSKYTINPAIAHGISEYVGSVEVGKVADLVLWSPAFFGVKPNMIIKGGFIALSQMGDANASIPTPQPVYYREMFAHHGKAKYDANITFVSQAAYDKGIKEELGLERQVLPVKNCRNITKKDMQFNDTTAHIEVNPETYHVFVDGKEVTSKPANKVSLAQLFSIF | 569 |
| 89 | M3PXD6 | MKKISRKEYVSMYGPTTGDKVRLGDTDLIAEVEHDYTIYGEELKFGGGKTLREGMSQSNNPSKEELDLIITNALIVDYTGIYKADIGIKDGKIAGIGKGGNKDMQDGVKNNLSVGPATEALAGEGLIVTAGGIDTHIHFISPQQIPTAFASGVTTMIGGGTGPADGTNATTITPGRRNLKWMLRAAEEYSMNLGFLAKGNASNDASLADQIEAGAIGFKIHEDWGTTPSAINHALDVADKYDVQVAIHTDTLNEAGCVEDTMAAIAGRTMHTFHTEGAGGGHAPDIIKVAGEHNILPASTNPTIPFTVNTEAEHMDMLMVCHHLDKSIKEDVQFADSRIRPQTIAAEDTLHDMGIFSITSSDSQAMGRVGEVITRTWQTADKNKKEFGRLKEEKGDNDNFRIKRYLSKYTINPAIAHGISEYVGSVEVGKVADLVLWSPAFFGVKPNMIIKGGFIALSQMGDANASIPTPQPVYYREMFAHHGKAKYDANITFVSQVAYDKGIKEELGLERQVLPVKNCRNITKKDMQFNDTTAHIEVNPETYHVFVDGKEVTSKPANKVSLAQLFSIF | 569 |
| 90 | M3Q5Y0 | MKKISRKEYVSMYGPTTGDKVRLGDTDLIAEVEHDYTIYGEELKFGGGKTLREGMSQSNNPSKEELDLIITNALIVDYTGIYKADIGIKDGKIAGIGKGGNKDMQDGVKNNLSVGPATEALAGEGLIVTAGGIDTHIHFISPQQIPTAFASGVTTMIGGGTGPADGTNATTITPGRRNLKWMLRAAEEYSMNLGFLAKGNTSNDASLADQIEAGAIGFKIHEDWGTTPSAINHALDVADKYDVQVAIHTDTLNEAGCVEDTMAAIAGRTMHTFHTEGAGGGHAPDIIKVAGEHNILPASTNPTIPFTVNTEAEHMDMLMVCHHLDKSIKEDVQFADSRIRPQTIAAEDTLHDMGIFSITSSDSQAMGRVGEVITRTWQTADKNKKEFGRLKEEKGDNDNFRIKRYLSKYTINPAIAHGISEYVGSVEVGKVADLVLWSPAFFGVKPNMIIKGGFIALSQMGDANASIPTPQPVYYREMFAHHGKAKYDANITFVSQAAYDKGIKEELGLERQVLPVKNCRNITKKDMQFNDTTAHIEVNPETYHVFVDGKEVTSKPANKVSLAQLFSIF | 569 |
| 91 | M3QJC9 | MKKISRKEYVSMYGPTTGDKVRLGDTDLIAEVEHDYTIYGEELKFGGGKTLREGMSQSNNPSKEELDLIITNALIVDYTGIYKADIGIKDGKIAGIGKGGNKDMQDGVKNNLSVGPATEALAGEGLIVTAGGIDTHIHFISPQQIPTAFASGVTTMIGGGTGPADGTNATTITPGRRNLKWMLRAAEEYSMNLGFLAKGNASNDASLADQIEAGAIGFKIHEDWGTTPSAINHALDVADKYDVQVAIHTDTLNEAGCVEDTMAAIAGRTMHTFHTEGAGGGHAPDIIKVAGEHNILPASTNPTIPFTVNTEAEHMDMLMVCHHLDKSIKEDVQFADSRIRPQTIAAEDTLHDMGIFSITSSDSQAMGRVGEVITRTWQTADKNKKEFGRLKEEKGDNDNFRIKRYLSKYTINPAIAHGISEYVGSVEVGKVADLVLWSPAFFGVKPNMIIKGGFIALSQMGDANASIPTPQPVYYREMFAHHGKAKYDANITFVSQAAYDKGIKEELGLERQVLPVKNCRNITKKDMQFNDTTAHIEVNPETYHVFVDGKEVTSKPANKVSLAQLFSIF | 569 |
| 92 | M3RQW6 | MKKISRKEYVSMYGPTTGDKVRLGDTDLIAEVEHDYTIYGEELKFGGGKTLREGMSQSNNPSKEELDLIITNALIVDYTGIYKADIGIKDGKIAGIGKGGNKDMQDGVKNNLSVGPATEALAGEGLIVTAGGIDTHIHFISPQQIPTAFASGVTTMIGGGTGPADGTNATTITPGRRNLKWMLRAAEEYSMNLGFLAKGNTSNDASLADQIEAGAIGFKIHEDWGTTPSAINHALDVADKYDVQVAIHTDTLNEAGCVEDTMAAIAGRTMHTFHTEGAGGGHAPDIIKVAGEHNILPASTNPTIPFTVNTEAEHMDMLMVCHHLDKSIKEDVQFADSRIRPQTIAAEDTLHDMGIFSITSSDSQAMGRVGEVITRTWQTADKNKKEFGRLKEEKGDNDNFRIKRYLSKYTINPAIAHGISEYVGSVEVGKVADLVLWSPAFFGVKPNMIIKGGFIALSQMGDANASIPTPQPVYYREMFAHHGKAKYDANITFVSQAAYDKGIKEELGLERQVLPVKNCRNITKKDMQFNDTTAHIEVNPETYHVFVDGKEVTSKPANKVSLAQLFSIF | 569 |
| 93 | M5YXK1 | MKKISRKEYVSMYGPTTGDKVRLGDTDLIAEVEHDYTIYGEELKFGGGKTLREGMSQSNNPSKEELDLIITNALIVDYTGIYKADIGIKDGKIAGIGKGGNKDMQDGVKNNLSVGPATEALAGEGLIVTAGGIDTHIHFISPQQIPTAFASGVTTMIGGGTGPADGTNATTITPGRRNLKWMLRAAEEYSMNLGFLAKGNASNDASLADQIEAGAIGFKIHEDWGTTPSAINHALDVADKYDVQVAIHTDTLNEAGCVEDTMAAIAGRTMHTFHTEGAGGGHAPDIIKVAGEHNILPASTNPTIPFTVNTEAEHMDMLMVCHHLDKSIKEDVQFADSRIRPQTIAAEDTLHDMGIFSITSSDSQAMGRVGEVITRTWQTADKNKKEFGRLKEEKGDNDNFRIKRYLSKYTINPAIAHGISEYVGSVEVGKVADLVLWSPAFFGVKPNMIIKGGFIALSQMGDANASIPTPQPVYYREMFAHHGKAKYDANITFVSQAAYDKGIKEELGLERQVLPVKNCRNITKKDMQFNDTTAHIEVNPETYHVFVDGKEVTSKPANKVSLAQLFSIF | 569 |
| 94 | N2BI39 | MTKINRQEYVSMYGPTTGDKIRLSDTELFAEIEKDYAIYGEEIKFGGGKTIRDGMAQSVSNSENELDSVITNAVIIDYTGIYKADIGIKNGKIFGIGKAGNKDTQDGVCDKLIVGTNTEVIAGEGLIVTAGGIDTHIHYISPTQIPTALYSGVTTMIGGGTGPAAGTSATTCTPGSWHMREMIRATQHYAMNFGFFGKGNSSNENALSKQIESGALGLKVHEDWGSTPAAINHALNIADKYDVQIAIHTDTLNEAGCMEDTLQAINGRTIHTFHTEGAGGGHAPDIIKAAGELHVLPASTNPTIPFTTNTADEHLDMLMVCHHLDKNIKEDVAFADSRIRPETIAAEDTLHDMGIFSITSSDSQAMGRVGEVIIRTWQTADKCKREFGALKEERGDNDNFRIKRYIAKYTINPAIAHGIADYVGSVEIGKIADLVIWKPSMFGVKPEMILKNGMIVAAKIGDSNASIPTPQPIVYADMFGSVGSARYDCGFTFVSKVAFDSNIKEKYGIERNILPVKNCRNITKKDMKYNDVVEKIEVDSETYEVKVNGVKITSKPISKVSLGQLYTLF | 569 |
| 95 | V5NL74 | MKKISRKEYASMYGPTTGDKVRLGDTDLIAEVEHDYTIYGEELKFGGGKTLREGMSQSNNPSKEELDLIITNALIVDYTGIYKADIGIKDGKIAGIGKGGNKDMQDGVKNNLSVGPATEALAGEGLIVTAGGIDTHIHFISPQQIPTAFASGVTTMIGGGTGPADGTNATTITPGRRNLKFMLRAAEEYSMNFGFLAKGNVSNDASLADQIEAGAIGFKIHEDWGTTPSAINHALDVADKYDVQVAIHTDTLNEAGCVEDTMAAIAGRTMHTFHTEGAGGGHAPDIIKVAGEHNILPASTNPTIPFTVNTEAEHMDMLMVCHHLDKSIKEDVQFADSRIRPQTIAAEDTLHDMGIFSITSSDSQAMGRVGEVITRTWQTADKNKKEFGRLKEEKGDNDNFRIKRYLSKYTINPAIAHGISEYVGSVEVGKVADLVLWSPAFFGVKPNMIIKGGFIALSQMGDANASIPTPQPVYYREMFAHHGKAKYDANITFVSQAAYDKGIKEELGLERQVLPVKNCRNITKKDMQFNDTTAHIEVNSETYHVFVDGKEVTSKPANKVSLAQLFSIF | 569 |
| 96 | X2I1L8 | MKKISRKEYASMYGPTTGDKVRLGDTDLIAEVEHDYTIYGEELKFGGGKTLREGMSQSNNPSKEELDLIITNALIVDYTGIYKADIGIKDGKIAGIGKGGNKDMQDGVKNNLSVGPATEALAGEGLIVTAGGIDTHIHFISPQQIPTAFASGVTTMIGGGTGPADGTNATTITPGRRNLKFMLRAAEEYSVNLGFLAKGNTSNDASLADQIEAGAIGFKIHEDWGTTPSAINHALDVADKYDVQVAIHTDTLNEAGCVEDTMAAIAGRTMHTFHTEGAGGGHAPDIIKVAGEHNILPASTNPTIPFTVNTEAEHMDMLMVCHHLDKSIKEDVQFADSRIRPQTIAAEDTLHDMGIFSITSSDSQAMGRVGEVITRTWQTADKNKKEFGRLKEEKGDNDNFRIKRYLSKYTINPAIAHGISEYVGSVEVGKVADLVLWSPAFFGVKPNMIIKGGFIALSQMGDANASIPTPQPVYYREMFAHHGKAKYDANITFVSQAAYDKGIKEELGLERQVLPVKNCRNITKKDMQFNDTTAHIEVNPETYHVFVDGKEVTSKPANKVSLAQLFSIF | 569 |
| 97 | A0A268TFG8 | MTKISRKQYASMFGPTVGDKVRLADTELFAEIEKDYTTYGEELKFGGGKTLRDGMAQSVSSDSSELDLVITNAMIIDYNGIYKADIGIKDGKIAGIGKAGNRDMQDGVCGNMTVGAATEALAAEGMIITAGGIDTHIHFISPQQIPTALYSGITTMIGGGTGPADGTNATTCTPGKWNLKEMIRAAEEYTMNLGFFGKGNSSNENALAEQIKGGALGLKIHEDWGATPSVINHALNVAEAYDVQVAIHTDTLNEAGCVEDTIKAIGGRTIHTFHTEGAGGGHAPDIIKIAGEPNILPASTNPTIPFTKNTADEHMDMLMVCHHLDKKIKEDVAFADSRIRPETIAAEDALHDMGIFSITSSDSQAMGRVGEVITRTWQTADKNKKEFGRLKEECGDNDNFRIKRYIAKYTINPAIAHGISEYVGSVSVGKYADLVMWHPAFFGIKPDMIIKCGMIAGARMGDANASIPTPEPVIYREMFG  HHGKAKFDTSITFVSKAAYEEGIKEKLGLDRVVLPVKNCRNITKKDMKNNDVTAHIEVNPETYEVKVEGKKVTSKPADKLSLAQLYNLF | 569 |
| 98 | A0A268U3W9 | MTKLSRKQYASMFGPTIGDKVRLADTELFAEIEKDYTVYGEELKFGGGKTLRDGMAQSVSSNVNELDLVITNAMIIDHNGIYKADIGIKNGKIAGIGKAGNKDMQDGVDDNMIVGAGTEALAGEGMIVTAGGIDTHIHFISPQQIPTALYSGITTMIGGGTGPADGTNATTCTPGKWNLKEMIRAAEEYTMNLGFFGKGNSSNENALAEQIKGGALGLKIHEDWGATPSVINHALNVAEKYDVQVAIHTDTLNEAGCVEDTIKAIGGRTIHTFHTEGAGGGHAPDIIKIAGEPNILPASTNPTIPFTKNTADEHLDMLMVCHHLDKKIKEDVAFADSRIRPETIAAEDTLHDMGIFSITSSDSQAMGRVGEVITRTWQTADKNKKEFGRLKEENGDNDNFRIKRYIAKYTINPAIAHGISEYIGSVEVGKFADLVIWHPAFFGIKPDIIIKGGMIAAARMGDPNASIPTPEPVIYREMFGHHGKAKYDTNITFVSQVAYEEGIKEKLGLERVILPVKNCRNITKKDMKNNSVTAHIEVNPETYEVKVEGKKVTSKPAEKLSLAQLYNLF | 569 |
| 99 | A0A8D6H666 | MKKISRKEYVSMYGPTTGDKVRLGDTDLILEVEHDYTTYGEEIKFGGGKTIRDGMGQTNSPSSHELDLVITNALIVDYTGIYKADIGIKNGKIHGIGKAGNKDLQDGVCNRLCVGPATEALAGEGLIVTAGGIDTHIHFISPQQIPTAFASGITTMIGGGTGPADGTNATTITPGRWNLKEMLRASEEYAMNLGYLGKGNVSFEPALIDQLEAGAIGFKIHEDWGSTPSAIHHALNIADK  YDVQVAIHTDTLNEAGCVEDTLEAIAGRTIHTFHTEGAGGGHAPDVIKMAGEFNILPASTNPTIPFTKNTEAEHMDMLMVCHHLDKNIKEDVEFADSRIRPQTIAAEDKLHDMGIFSITSSDSQAMGRVGEVITRTWQTADKNKKEFGRLPEEKGDNDNFRIKRYVAKYTINPAITHGISEYVGSVEVGKYADLVLWSPAFFGIKPNMIIKGGFIALSQMGDANASIPTPQPVYYREMFG  HHGKAKFDTNITFVSQVAYENGIKHELGLQRIVLPVKNCRNITKKDLKFNDVTAHIEVNPETYKVKVDGNEVTSHAADKLSLAQLYNLF | 569 |
| 100 | A0A8D6H6V3 | MKKISRKEYVSMYGPTTGDKVRLGDTDLILEVEHDYTTYGEEIKFGGGKTIRDGMGQTNSPSSHELDLVITNALIVDYTGIYKADIGIKNGKIHGIGKAGNKDLQDGVCNRLCVGPATEALAGEGLIVTAGGIDTHIHFISPQQIPTAFASGITTMIGGGTGPADGTNATTITPGRWNLKEMLRASEEYAMNLGYLGKGNVSFEPALIDQLEAGAIGFKIHEDWGSTPSAINHALNIADK  YDVQVAIHTDTLNEAGCVEDTLQAIAGRTIHTFHTEGAGGGHAPDVIKMAGEFNILPASTNPTIPFTKNTEAEHMDMLMVCHHLDKNIKEDVEFAGSRIRPQTIAAEDKLHDMGIFSITSSDSQAMGRVGEVITRTWQTADKNKKEFGRLPEEKGDNDNFRIKRYIAKYTINPAITHGISEYVGSVEVGKFADLVLWSPAFFGIKPNMIIKGGFIALSQMGDANASIPTPQPVYYREMFG  HHGKAKYDTNITFVSQVAYENGIKHELGLQRIVLPVKNCRNITKKDLKFNDVTAHIEVNPETYKVKVDGNEVTSHAADKLSLAQLYNLF | 569 |
| 101 | C7BXS4 | MKKISRKEYVSMYGPTTGDKVRLGDTDLIAEVEHDYTIYGEELKFGGGKTLREGMSQSNNPSKEELDLIITNALIVDYTGIYKADIGIKDGKIAGIGKGGNKDMQDGVKNNLSVGPATEALAGEGLIVTAGGIDTHIHFISPQQIPTAFASGVTTMIGGGTGPADGTNATTITPGRRNLKWMLRAAEEYSMNLGFLAKGNASNDASLADQIEAGAIGFKIHEDWGTTPSAINHALDVADKYDVQVAIHTDTLNEAGCVEDTMAAIAGRTMHTFHTEGAGGGHAPDIIKVAGEHNILPASTNPTIPFTVNTEAEHMDMLMVCHHLDKSIKEDVQFADSRIRPQTIAAEDTLHDMGIFSITSSDSQAMGRVGEVITRTWQTADKNKKEFGRLKEEKGDNDNFRIKRYLSKYTINPAIAHGISEYVGSVEVGKVADLVLWSPAFFGVKPNMIIKGGFIALSQMGDANASIPTPQPVYYREMFAHHGKAKYDANITFVSQAAYDKGIKEELGLERQVLPVKNCRNITKKDMQFNDTTAHIEVNPETYHVFVDGKEVTSKPANKVSLAQLFSIF | 569 |
| 102 | D0IQV3 | MKKISRKEYVSMYGPTTGDKVRLGDTDLIAEVEHDYTIYGEELKFGGGKTLREGMSQSNNPSKEELDLIITNALIVDYTGIYKADIGIKDGKIAGIGKGGNKDMQDGVKNNLSVGPATEALAGEGLIVTAGGIDTHIHFISPQQIPTAFASGVTTMIGGGTGPADGTNATTITPGRRNLKWMLRAAEEYSMNLGFLAKGNTSNDASLADQIEAGAIGFKIHEDWGTTPSAINHALDVADKYDVQVAIHTDTLNEAGCVEDTMAAIAGRTMHTFHTEGAGGGHAPDIIKVAGEHNILPASTNPTIPFTVNTEAEHMDMLMVCHHLDKSIKEDVQFADSRIRPQTIAAEDTLHDMGIFSITSSDSQAMGRVGEVITRTWQTADKNKKEFGRLKEEKGDNDNFRIKRYLSKYTINPAIAHGISEYVGSVEVGKVADLVLWSPAFFGVKPNMIIKGGFIALSQMGDANASIPTPQPVYYREMFAHHGKAKYDANITFVSQAAYDKGIKEELGLERQVLPVKNCRNITKKDMQFNDTTAHIEVNPETYHVFVDGKEVTSKPATKVSLAQLFSIF | 569 |
| 103 | E1Q187 | MKKISRKEYVSMYGPTTGDKVRLGDTDLIAEVEHDYTIYGEELKFGGGKTLREGMSQSNNPSKEELDLIITNALIVDYTGIYKADIGIKDGKIAGIGKGGNKDMQDGVKNNLSVGPATEALAGEGLIVTAGGIDTHIHFISPQQIPTAFASGVTTMIGGGTGPADGTNATTITPGRRNLKWMLRAAEEYSMNLGFLAKGNASNDASLADQIEAGAIGFKIHEDWGTTPSAINHALDVADKYDVQVAIHTDTLNEAGCVEDTMAAIAGRTMHTFHTEGAGGGHAPDIIKVAGEHNILPASTNPTIPFTVNTEAEHMDMLMVCHHLDKSIKEDVQFADSRIRPQTIAAEDTLHDMGIFSITSSDSQAMGRVGEVITRTWQTADKNKKEFGRLKEEKGDNDNFRIKRYLSKYTINPAIAHGISEYVGSVEVGKVADLVLWSPAFFGVKPNMIIKGGFIALSQMGDANASIPTPQPVYYREMFAHHGKAKYDANITFVSQAAYDKGIKEELGLERQVLPVKNCRNITKKDMQFNDTTAHIEVNPETYHVFVDGKEVTSKPANKVSLAQLFSIF | 569 |
| 104 | E1Q3V3 | MKKISRKEYVSMYGPTTGDKVRLGDTDLIAEVEHDYTIYGEELKFGGGKTLREGMSQSNNPSKEELDLIITNALIVDYTGIYKADIGIKDGKIAGIGKGGNKDMQDGVKNNLSVGPATEALAGEGLIVTAGGIDTHIHFISPQQIPTAFASGVTTMIGGGTGPADGTNATTITPGRRNLKWMLRAAEEYSMNLGFLAKGNASNDASLADQIEAGAIGFKIHEDWGTTPSAINHALDVADKYDVQVAIHTDTLNEAGCVEDTMAAIAGRTMHTFHTEGAGGGHAPDIIKVAGEHNILPASTNPTIPFTVNTEAEHMDMLMVCHHLDKSIKEDVQFADSRIRPQTIAAEDTLHDMGIFSITSSDSQAMGRVGEVITRTWQTADKNKKEFGRLKEEKGDNDNFRIKRYLSKYTINPAIAHGISEYVGSVEVGKVADLVLWSPAFFGVKPNMIIKGGFIALSQMGDANASIPTPQPVYYREMFAHHGKAKYDANITFVSQAAYDKGIKEELGLERQVLPVKNCRNITKKDMQFNDTTAHIEVNPETYHVFVDGKEVTSKPANKVSLAQLFSIF | 569 |
| 105 | E8QRG4 | MKKISRKEYVSMYGPTTGDKVRLGDTDLIAEVEHDYTIYGEELKFGGGKTLREGMSQSNNPSKEELDLIITNALIVDYTGIYKADIGIKDGKIAGIGKGGNKDMQDGVKNNLSVGPATEALAGEGLIVTAGGIDTHIHFISPQQIPTAFASGVTTMIGGGTGPADGTNATTITPGRRNLKFMLRAAEEYSMNFGFLAKGNVSNDASLADQIEAGAIGFKIHEDWGTTPSAINHALDVADKYDVQVAIHTDTLNEAGCVEDTMAAIAGRTMHTFHTEGAGGGHAPDIIKVAGEHNILPASTNPTIPFTVNTEAEHMDMLMVCHHLDKSIKEDVQFADSRIRPQTIAAEDTLHDMGIFSITSSDSQAMGRVGEVITRTWQTADKNKKEFGRLKEEKGDNDNFRIKRYLSKYTINPAIAHGISEYVGSVEVGKVADLVLWSPAFFGVKPNMIIKGGFIALSQMGDANASIPTPQPVYYREMFAHHGKAKYDANITFVSQAAYDKGIKEELGLERQVLPVKNCRNITKKDMQFNDTTAHIEVNPETYHVFVDGKEVTSKPANKVSLAQLFSIF | 569 |
| 106 | E8QTS3 | MKKISRKEYVSMYGPTTGDKVRLGDTELIAEVEHDYTIYGEELKFGGGKTLREGMSQSNNPSKEELDLIITNALIVDYTGIYKADIGIKDGKIAGIGKGGNKDMQDGVKNNLSVGPATEALAGEGLIVTAGGIDTHIHFISPQQIPTAFASGVTTMIGGGTGPADGTNATTITPGRRNLKWMLRAAEEYSMNLGFLAKGNTSNDASLADQIEAGAIGFKIHEDWGTTPSAINHALDVADKYDVQVAIHTDTLNEAGCVEDTMAAIAGRTMHTFHTEGAGGGHAPDIIKVAGEHNILPASTNPTIPFTVNTEAEHMDMLMVCHHLDKSIKEDVQFADSRIRPQTIAAEDTLHDMGIFSITSSDSQAMGRVGEVITRTWQTADKNKKEFGRLKEEKGDNDNFRIKRYLSKYTINPAIAHGISEYVGSVEVGKVADLVLWSPAFFGVKPNMIIKGGFIALSQMGDANASIPTPQPVYYREMFAHHGKAKYDANITFVSQAAYDKGIKEELGLERQVLPVKNCRNITKKDMQFNDTTAHIEVNPETYHVFVDGKEVTSKPANKVSLAQLFSIF | 569 |
| 107 | H5VC53 | MYGPTTGDKVRLGDTDLILEVEHDCTTYGEEIKFGGGKTIRDGMAQTNSPSSHELDLVLTNALIVDYTGIYKADIGIKNGKIHGIGKAGNKDMQDGVCNNLCVGPATEALAAEGLIVTAGGIDTHIHFISPQQIPTAFASGITTMIGGGTGPADGTNATTITPGRWNLKTMLRASEEYAMNLGYLGKGNVSYEPSLVDQLEAGAIGFKIHEDWGSTPAAIYHCLNVADKYDVQVAIHTDTLNEAGCVEDTLQAIAGRTIHTFHTEGAGGGHAPDVIKMSGEFNILPASTNPTIPFTVNTEAEHMDMLMVCHHLDKNIKEDVQFADSRIRPQTIAAEDKLHDMGIFSITSSDSQAMGRVGEVITRTWQTADKNKKEFGRLPEEKGDNDNFRIKRYISKYTINPAIAHGISEYVGSVEVGKFADLVLWSPAFFGIKPNMIIKGGFIALSQMGDANASIPTPQPVYYREMFGHHGKAKFDTNITFVSQVAYDNGIKEELGLQRVVLPVKNCRNITKKDLKFNDVTAHIEVNPETYKVKVDGKEVTSKAADKISLAQLYNLF | 558 |
| 108 | V6L8R1 | MKKISRKEYVSMYGPTTGDKVRLGDTDLIAEVEHDYTIYGEELKFGGGKTLREGMSQSNNPSKEELDLIITNALIVDYTGIYKADIGIKDGKIAGIGKGGNKDMQDGVKNNLSVGPATEALAGEGLIVTAGGIDTHIHFISPQQIPTAFASGVTTMIGGGTGPADGTNATTITPGRRNLKWMLRAAEEYSMNLGFLAKGNASNDASLADQIEAGAIGFKIHEDWGTTPSAINHALDVADKYDVQVAIHTDTLNEAGCVEDTMAAIAGRTMHTFHTEGAGGGHAPDIIKVAGEHNILPASTNPTIPFTVNTEAEHMDMLMVCHHLDKSIKEDVQFADSRIRPQTIAAEDTLHDMGIFSITSSDSQAMGRVGEVITRTWQTADKNKKEFGRLKEEKGDNDNFRIKRYLSKYTINPAIAHGISEYVGSVEVGKVADLVLWSPAFFGVKPNMIIKGGFIALSQMGDANASIPTPQPVYYREMFAHHGKAKYDANITFVSQAAYDKGIKEELGLERQVLPVKNCRNITKKDMQFNDTTAHIEVNPETYHVFVDGKEVTSKPANKVSLAQLFSIF | 569 |
| 109 | A0A268TGN5 | MTKISRKLYASMFGPTVGDKVRLADTELFAEIEKDYTTYGEEIKFGGGKTIRDGMAQSVSADTHELDLVITNAMIIDYNGIYKADIGIKDGKIAGIGKAGNKDMQDGVCGNMTVGAATEALAGEGMIITAGGIDTHIHFISPQQIPTALYSGITTMIGGGTGPADGTNATTCTPGRWNLKEMIRAAEEYTMNLGFFGKGNSSNEAALADQIKAGALGLKIHEDWGSTPSVINHALNVAEE  YDVQVAIHTDTLNEAGCVEDTIKAIGGRTIHTFHTEGAGGGHAPDIIKIAGEPNILPASTNPTIPFTKNTADEHMDMLMVCHHLDKRIKEDVAFADSRIRPETIAAEDALHDMGIFSITSSDSQAMGRVGEVITRTWQTADKNKKEFGKLKEETGENDNFRIKRYVAKYTINPAIAHGISEYVGSVEVGKFADLVMWHPAFFGIKPDIIIKGGMIVAARMGDPNASIPTPEPVIYREMFGHHGKARFDTSITFVSKAAYEEGIKEKLGLDRVVLPVKNCRNITKKDMKNNDVITHIEVNPETYEVKVEGKKVTSKPAQKLSLAQLYNLF | 569 |
| 110 | E8QDC7 | MKKISRKEYVSMYGPTTGDKVRLGDTDLIAEVEHDYTIYGEELKFGGGKTLREGMSQSNNPSKEELDLIITNALIVDYTGIYKADIGIKDGKIAGIGKGGNKDMQDGVKNNLSVGPATEALAGEGLIVTAGGIDTHIHFISPQQIPTAFASGVTTMIGGGTGPADGTNATTITPGRRNLKWMLRAAEEYSMNLGFLAKGNASNDASLADQIEAGAIGFKIHEDWGTTPSAINHALDVADKYDVQVAIHTDTLNEAGCVEDTMAAIAGRTMHTFHTEGAGGGHAPDIIKVAGEHNILPASTNPTIPFTVNTEAEHMDMLMVCHHLDKSIKEDVQFADSRIRPQTIAAEDTLHDMGIFSITSSDSQAMGRVGEVITRTWQTADKNKKEFGRLKEEKGDNDNFRIKRYLSKYTINPAIAHGISEYVGSVEVGKVADLVLWSPAFFGVKPNMIIKGGFIALSQMGDANASIPTPQPVYYREMFAHHGKAKYDANITFVSQAAYDKGIKEELGLERQVLPVKNCRNITKKDMQFNDTTAHIEVNPETYHVFVDGKEVTSKPANKVSLAQLFSIF | 569 |
| 111 | A0A849NN23 | MKKISRKEYVSMYGPTTGDKVRLGDTDLIAEVEHDYTIYGEELKFGGGKTLREGMSQSNNPSKEELDLIITNALIVDYTGIYKADIGIKDGKIAGIGKGGNKDMQDGVKNNLSVGPATEALAGEGLIVTAGGIDTHIHFISPQQIPTAFASGVTTMIGGGTGPADGTNATTITPGRRNLKWMLRAAEEYSMNLGFLAKGNASNDASLADQIEAGAIGFKIHEDWGTTPSAINHALDVADKYDVQVAIHTDTLNEAGCVEDTMAAIAGRTMHTFHTEGAGGGHAPDIIKVAGEHNILPASTNPTIPFTVNTEAEHMDMLMVCHHLDKSIKEDVQFADSRIRPQTIAAEDTLHDMGIFSITSSDSQAMGRVGEVITRTWQTADKNKKEFGRLKEEKGDNDNFRIKRYLSKYTINPAIAHGISEYVGSVEVGKVADLVLWSPAFFGVKPNMIIKGGFIALSQMGDANASIPTPQPVYYREMFAHHGKAKYDANITFVSQAAYDKGIKEELGLERQVLPVKNCRNITKKDMQFNDTTAHIEVNPETYHVFVDGKEVTSKPANKVSLAQLFSIF | 569 |
| 112 | D0K1Z5 | MKKISRKEYASMYGPTTGDKVRLGDTDLIAEVEHDYTIYGEELKFGGGKTLREGMSQSNNPSKEELDLIITNALIVDYTGIYKADIGIKDGKIAGIGKGGNKDTQDGVKNNLSVGPATEALAGEGLIVTAGGIDTHIHFISPQQIPTAFASGVTTMIGGGTGPADGTNATTITPGRRNLKWMLRAAEEYSMNLGFLAKGNTSNDASLADQIEAGAIGFKIHEDWGTTPSAINHALDVADKYDVQVAIHTDTLNEAGCVEDTMAAIAGRTMHTFHTEGAGGGHAPDIIKVAGEHNILPASTNPTIPFTVNTEAEHMDMLMVCHHLDKNIKEDVQFADSRIRPQTIAAEDTLHDMGIFSITSSDSQAMGRVGEVITRTWQTADKNKKEFGRLKEEKGDNDNFRIKRYLSKYTINPAIAHGISEYVGSVEVGKVADLVLWSPAFFGVKPNMIIKGGFIALSQMGDANASIPTPQPVYYREMFAHHGKAKYDANITFVSQAAYDKGIKEELGLERQVLPVKNCRNITKKDMQFNDTTAHIEVNPETYHVFVDGKEVTSKPANKVSLAQLFSIF | 569 |
| 113 | I0EV22 | MKKISRKEYVSMYGPTTGDKVRLGDTDLIAEVEHDYTIYGEELKFGGGKTLREGMSQSNNPSKEELDLIITNALIVDYTGIYKADIGIKDGKIAGIGKGGNKDMQDGVKNNLSVGPATEALAGEGLIVTAGGIDTHIHFISPQQIPTAFASGVTTMIGGGTGPADGTNATTITPGRRNLKWMLRAAEEYSMNLGFLAKGNASNDASLADQIEAGAIGFKIHEDWGTTPSAINHALDVADKYDVQVAIHTDTLNEAGCVEDTMAAIAGRTMHTFHTEGAGGGHAPDIIKVAGEHNILPASTNPTIPFTVNTEAEHMDMLMVCHHLDKSIKEDVQFADSRIRPQTIAAEDTLHDMGIFSITSSDSQAMGRVGEVITRTWQTADKNKKEFGRLKEEKGDNDNFRIKRYLSKYTINPAIAHGISEYVGSVEVGKVADLVLWSPAFFGVKPNMIIKGGFIALSQMGDANASIPTPQPVYYREMFAHHGKAKYDANITFVSQVAYDKGIKEELGLERQVLPVKNCRNITKKDMQFNDTTAHIEVNPETYHVFVDGKEVTSKPANKVSLAQLFSIF | 569 |
| 114 | A0A268TRY1 | MTKISRKQYASMFGPTVGDKVRLADTELFAEIEKDYTTYGEELKFGGGKTLRDGMAQSVSSDSHELDLVITNAMIIDYNGIYKADIGIKDGKIAGIGKAGNKDMQDGVCGNMTVGAATEALAAEGMIVTAGGIDTHIHFISPQQIPTALYSGITTMIGGGTGPADGTNATTCTPGKWNLKEMIRAAEEYTMNLGFFGKGNSSNENSLAEQIKGGALGLKIHEDWGATPSVINHALNVADEYDVQVAIHTDTLNEAGCVEDTIKAIAGRTIHTFHTEGAGGGHAPDIIKIAGEPNILPASTNPTIPFTKNTADEHLDMLMVCHHLDKKIKEDVAFADSRIRPETIAAEDALHDMGIFSITSSDSQAMGRVGEVITRTWQTADKNKKEFGRLKEETGDNDNFRIKRYIAKYTINPAIAHGISEYIGSVEIGKYADLVIWHPAFFGIKPEMIIKCGMIAGARMGDSNASIPTPEPVIYREMFA  HHGKAKFDTNITFVSKVAYEDGIKEKLKLDRIVLPVKNCRNVTKKDMKNNDVTAHIDVNPETYEVKVDGKKVTSKPADKLSLAQLYNLF | 569 |
| 115 | A0A268TS20 | MTKISRKQYASMFGPTVGDKVRLADTELFAEIEKDYTIYGEELKFGGGKTLRDGMAQSVSSDTRELDLVITNAMIIDYNGIYKADIGIKDGKIAGIGKAGNKDMQDGVCGNMAVGAGTEALAGEGMIVTAGGIDTHIHFISPQQIPTALYSGITTMIGGGTGPADGTNATTCTPGRWNLKEMIRAAEEYTMNLGFFGKGNSSNENALAEQVKAGALGLKIHEDWGATPSVINHALNVAEAYDVQVAIHTDTLNEAGCVEDTIKAIAGRTIHTFHTEGAGGGHAPDIIKIAGEPNILPASTNPTIPFTKNTADEHLDMLMVCHHLDKKIKEDVAFADSRIRPETIAAEDTLHDMGIFSITSSDSQAMGRVGEVITRTWQTADKNKKEFGRLKEEKGDNDNFRIKRYVAKYTINPAIAHGISEYVGSVEVGKYADLVLWNPAFFGIKPEMIIKCGMIAGARMGDANASIPTPEPVIYREMFAHHGKAKFDTNITFVSQAAYADGIKEKLGLDRIVLPVKNCRNITKKDMKNNDVTAHIEVNPETYEVKVGGKKISSKPADKLSLAQLYNLF | 569 |
| 116 | B9XWX8 | MKKISRKEYASMYGPTTGDKVRLGDTDLIAEVEHDYTIYGEELKFGGGKTLREGMSQSNNPSKEELDLIITNALIVDYTGIYKADIGIKDGKIAGIGKGGNKDMQDGVKNNLSVGPATEALAGEGLIVTAGGIDTHIHFISPQQIPTAFASGVTTMIGGGTGPADGTNATTITPGRRNLKWMLRAAEEYSMNLGFLAKGNASNDASLADQIEAGAIGFKIHEDWGTTPSAINHALDVADKYDVQVAIHTDTLNEAGCVEDTMAAIAGRTMHTFHTEGAGGGHAPDIIKVAGEHNILPASTNPTIPFTVNTEAEHMDMLMVCHHLDKSIKEDVQFADSRIRPQTIAAEDTLHDMGIFSITSSDSQAMGRVGEVITRTWQTADKNKKEFGRLKEEKGDNDNFRIKRYLSKYTINPAIAHGISEYVGSVEVGKVADLVLWSPAFFGVKPNMIIKGGFIALSQMGDANASIPTPQPVYYREMFAHHGKAKYDANITFVSQAAYDKGIKEELGLERQVLPVKNCRNITKKDMQFNDTTAHIDVNPETYHVFVDGKEVTSKPATKVSLAQLFSIF | 569 |
| 117 | A0A268UBN6 | MTKISRKQYASMFGPTVGDKVRLADTELFAEIEKDYTIYGEEIKFGGGKTIRDGMGQSVSHSENELDLVITNAMIIDYTGIYKADIGIKNGKIAGIGKAGNKDMQDGVCDNMIVGAGTEALAGEGLIITAGGIDTHIHYISPQQIPTALYSGVTTMIGGGTGPADGTNATTCTPGRYNLKEMLRAAEEYTMNLGFFGKGNCSNEAALADQIKAGALGLKIHEDWGSTPAVINHALNVAEEYDIQIAIHTDTLNEAGCVEDTINAINGRTIHTFHTEGAGGGHAPDIIKIAGELNILPASTNPTIPFTKNTADEHLDMLMVCHHLDKKIKEDVAFADSRIRPETIAAEDTLHDMGIFSITSSDSQAMGRVGEVITRTWQTADKNKKEFGKLKEEKGDNDNFRIKRYISKYTINPAIAHGISEYVGSVEVGKFADLVIWQPAFFGIKPEMILKCGMIVAARMGDPNASIPTPEPVIYREMFGHHGKAKFDVNITFVSKAAYEDNIKEKLGLERVVLPVKNCRNITKKDMKNNDVTAHIEVDPETYAVKVDGKKVTSKPADKLSLAQLYNLF | 569 |
| 118 | C3XHC3 | MIKINRQEYVSMYGPTTGDKIRLGDTELFAEIEKDYAIYGEEIKFGGGKTIRDGMAQSVSDSENELDSVITNAVIIDYTGIYKADIGIKNGKIFGIGKAGNKDTQDGVCDKLIVGTNTEVIAGEGLIVTAGGIDTHIHYISPTQIPTALYSGVTTMIGGGTGPAAGTSATTCTPGSWHMREMIRATQHYAMNFGFFGKGNSSNENALSKQIESGALGLKVHEDWGSTPAAINHALNIADKYDVQIAIHTDTLNEAGCMEDTLQAINGRTIHTFHTEGAGGGHAPDIIKAAGELHVLPASTNPTIPFTTNTADEHLDMLMVCHHLDKNIKEDVAFADSRIRPETIAAEDTLHDMGIFSITSSDSQAMGRVGEVIIRTWQTADKCKREFGALKEEKGDNDNFRIKRYIAKYTINPAIAHGIADYVGSVEIGKIADLVIWKPSMFGVKPEMILKNGMIVAAKIGDSNASIPTPQPIVYADMFGSVGSAKYDCGFTFVSKVAFDSNIKEKYGIERNILPVKNCRNITKKDMKYNDVVEKIEVDSETYEVKVNGVKITSKPVSKVSLGQLYTLF | 569 |
| 119 | A0A099TW07 | MIKISRKQYASMYGPTTGDKVRLGDTNLFAEIEKDYTTYGEEIKFGGGKTIRDGMAQSASSYENELDVVITNAMIIDYTGIYKADIGIKNGKIVGIGKAGNPDTQDNVSKTMVVGAATEVIAGEGQIVTAGGIDTHIHFISPTQIPTALYSGVTTMIGGGTGPAAGTNATTCTPGKYNMQQMLGAAEEYAMNIGIFGKGNSSNEEALEEQIKAGALGLKVHEDWGSTPAAINHALNVAEKYDVQVAIHTDTLNEAGCVEDTMRAIGGRTIHTFHTEGAGGGHAPDIIKAAGELNILPASTNPTIPFTKNTADEHLDMLMVCHHLDKKIKEDVAFADSRIRPETIAAEDTLHDMGIFSITSSDSQAMGRVGEVIIRTWQTANKCKNEFGALKEECGENDNFRIKRYISKYTINPAIAHGISEYVGSIEVGKFADLVLWKPSMFGVKPEMILKNGMIVAAKIGDSNASIPTPEPIVYAPMFGSHGKAKYNCAITFVSKVAYDTNIKEKFGLQRILLPVKNCRKITKKDMQFNDVVTPIKVNPETYEVSVNGKKITSKSVDSVSLGQLYCLF | 569 |
| 120 | A0A0K9MV2 | MKKISRKEYVSMYGPTTGDKVRLGDTDLIAEVEHDYTIYGEELKFGGGKTLREGMSQSNNPSKEELDLIITNALIVDYTGIYKADIGIKDGKIAGIGKGGNKDMQDGVKNNLSVGPATEALAGEGLIVTAGGIDTHIHFISPQQIPTAFASGVTTMIGGGTGPADGTNATTITPGRRNLKWMLRAAEEYSMNLGFLAKGNASNDASLADQIEAGAIGLKIHEDWGTTPSAINHALDVADKYDVQVAIHTDTLNEAGCVEDTMAAIAGRTMHTFHTEGAGGGHAPDIIKVAGEHNILPASTNPTIPFTVNTEAEHMDMLMVCHHLDKSIKEDVQFADSRIRPQTIAAEDTLHDMGIFSITSSDSQAMGRVGEVITRTWQTADKNKKEFGRLKEEKGDNDNFRIKRYLSKYTINPAIAHGISEYVGSVEVGKVADLVLWSPAFFGVKPNMIIKGGFIALSQMGDANASIPTPQPVYYREMFAHHGKAKYDANITFVSQAAYDKGIKEELGLERQVLPVKNCRNITKKDMQFNDTTAHIEVNPETYHVFVDGKEVTSKPANKVSLAQLFSIF | 569 |
| 121 | A0A2T6RR45 | MKKISRKEYVSMYGPTTGDKVRLGDTDLIAEVEHDYTIYGEELKFGGGKTLREGMSQSNNPSKEELDLIITNALIVDYTGIYKADIGIKDGKIAGIGKGGNKDTQDGVKNNLSVGPATEALAGEGLIVTAGGIDTHIHFISPQQIPTAFASGVTTMIGGGTGPADGTNATTITPGRRNLKFMLRAAEEYSMNFGFLAKGNASNDASLADQIEAGAIGLKIHEDWGTTPSAINHALDVADKYDVQVAIHTDTLNEAGCVEDTMAAIAGRTMHTYHTEGAGGGHAPDIIRVAGEHNILPASTNPTIPFTVNTEAEHMDMLMVCHHLDKSIKEDVQFADSRIRPQTIAAEDTLHDMGIFSITSSDSQAMGRVGEVITRTWQTADKNKKEFGRLKEEKGDNDNFRIKRYLSKYTINPAIAHGISEYVGSVEVGKVADLVLWSPAFFGVKPNMIIKGGFIALSQMGDANASIPTPQPVYYREMFAHHGKAKYDANITFVSQAAYDKGIKEELGLERQVLPVKNCRNITKKDMQFNDTTAHIEVNSETYHVFVDGKEVTSKPANKVSLAQLFSIF | 569 |
| 122 | A0A2T6V0D | MKKISRKEYVSMYGPTTGDKVRLGDTDLIAEVEHDYTIYGEELKFGGGKTLREGMSQSNNPSKEELDLIITNALIVDYTGIYKADIGIKNGKIAGIGKGGNKDMQDGVKNNLSVGPATEALAGEGLIVTAGGIDTHIHFISPQQIPTAFASGVTTMIGGGTGPADGTNATTITPGRRNLKWMLRAAEEYSMNLGFLAKGNASNDASLADQIEAGAIGFKIHEDWGTTPSAINHALDVADKYDVQVAIHTDTLNEAGCVEDTMAAIAGRTMHTFHTEGAGGGHAPDIIKVAGEHNILPASTNPTIPFTVNTEAEHMDMLMVCHHLDKSIKEDVQFADSRIRPQTIAAEDTLHDMGIFSITSSDSQAMGRVGEVITRTWQTADKNKKEFGRLKEEKGDNDNFRIKRYLSKYTINPAIAHGISEYVGSVEVGKVADLVLWSPAFFGVKPNMIIKGGFIALSQMGDANASIPTPQPVYYREMFAHHGKAKYDANITFVSQAAYDKGIKEELGLERQVLPVKNCRNITKKDMQFNDTTAHIEVNPETYHVFVDGKEVTSKPANKVSLAQLFSIF | 569 |
| 123 | A0A3Q9DMJ6 | MKKISRKEYVSMYGPTTGDKVRLGDTDLIAEVEHDYTIYGEELKFGGGKTLREGMSQSNNPSKEELDLIITNALIVDYTGIYKADIGIKDGKIAGIGKGGNKDTQDGVKNNLSVGPATEALAGEGLIVTAGGIDTHIHFISPQQIPTAFASGITTMIGGGTGPADGTNATTITPGRRNLKWMLRAAEEYSMNLGFLAKGNTSNDASLADQIEAGAIGFKIHEDWGTTPSAINHALDVADK  YDVQVAIHTDTLNEAGCVEDTMAAIAGRTMHTFHTEGAGGGHAPDIIKVAGEHNILPASTNPTIPFTVNTEAEHMDMLMVCHHLDKNIKEDVQFADSRIRPQTIAAEDTLHDMGIFSITSSDSQAMGRVGEVITRTWQTADKNKKEFGRLKEEKGDNDNFRIKRYLSKYTINPAIAHGISEYVGSVEVGKVADLVLWSPAFFGVKPNMIIKGGFIALSQMGDANASIPTPQPVYYREMFAHHGKAKYDANITFVSQAAYDKGIKEELGLERQVLPVKNCRNITKKDMQFNDTTAHIEVNPETYHVFVDGKEVTSKPANKVSLAQLFSIF | 569 |
| 124 | A0A438R6B6 | MKKISRKEYVSMYGPTTGDKVRLGDTDLIAEVEHDYTIYGEELKFGGGKTLREGMSQSNNPSKEELDLIITNALIVDYTGIYKADIGIKDGKIAGIGKGGNKDTQDGVKNNLSVGPATEALAGEGLIVTAGGIDTHIHFISPQQIPTAFASGVTTMIGGGTGPADGTNATTITPGRRNLKFMLRAAEEYSMNFGFLAKGNASNDASLADQIEAGAIGLKIHEDWGTTPSAINHALDVADKYDVQVAIHTDTLNEAGCVEDTMAAIAGRTMHTYHTEGAGGGHAPDIIKVAGEYNILPASTNPTIPFTVNTEAEHMDMLMVCHHLDKSIKEDVQFADSRIRPQTIAAEDTLHDMGIFSITSSDSQAMGRVGEVITRTWQTADKNKKEFGRLKEEKGDNDNFRIKRYLSKYTINPAIAHGISEYVGSVEVGKVADLVLWSPAFFGVKPNMIIKGGFIALSQMGDANASIPTPQPVYYREMFAHHGKAKYDANITFVSQAAYDKGIKEELGLERQVLPVKNCRNITKKDMQFNDTTAHIEVNPETYHVFVDGKEVTSKPANKVSLAQLFSIF | 569 |
| 124 | A0A438SUZ0 | MKKISRKEYVSMYGPTTGDKVRLGDTDLIAEVEHDYTIYGEELKFGGGKTLREGMSQSNNPSKEELDLIITNALIVDYTGIYKADIGIKDGKIAGIGKGGNKDMQDGVKNNLSVGPATEALAGEGLIVTAGGIDTHIHFISPQQIPTAFASGVTTMIGGGTGPADGTNATTITPGKRNLKWMLRAAEEYSMNLGFLAKGNTSNDASLADQIEAGAIGFKIHEDWGTTPSAINHALDVADKYDVQVAIHTDTLNEAGCVEDTMAAIAGRTMHTFHTEGAGGGHAPDIIKVAGEHNILPASTNPTIPFTVNTEAEHMDMLMVCHHLDKSIKEDVQFADSRIRPQTIAAEDTLHDMGIFSITSSDSQAMGRVGEVITRTWQTADKNKKEFGRLKEEKGDNDNFRIKRYLSKYTINPAIAHGISEYVGSVEVGKVADLVLWSPAFFGVKPNMIIKGGFIALSQMGDANASIPTPQPVYYREMFAHHGKAKYDANITFVSQAAYDKGIKEELGLERQVLPVKNCRNITKKDMQFNDTTAHIEVNPETYHVFVDGKEVTSKPANKVSLAQLFSIF | 569 |
| 126 | A0A496HBV3 | MKKISRKEYVSMYGPTTGDKVRLGDTDLIAEVEHDYTIYGEELKFGGGKTLREGMSQSNNPSKEELDLVITNALIVDYTGIYKADIGIKDGKIAGIGKGGNKDMQDGVKNNLSVGPATEALAGEGLIVTAGGIDTHIHFISPQQIPTAFASGVTTMIGGGTGPADGTNATTITPGRRNLKWMLRAAEEYSMNLGFLAKGNASNDASLADQIEAGAIGFKIHEDWGTTPSAINHALDVADKYDVQVAIHTDTLNEAGCVEDTMAAIAGRTMHTFHTEGAGGGHAPDIIKVAGEHNILPASTNPTIPFTVNTEAEHMDMLMVCHHLDKSIKEDVQFADSRIRPQTIAAEDTLHDMGIFSITSSDSQAMGRVGEVITRTWQTADKNKKEFGRLKEEKGDNDNFRIKRYLSKYTINPAIAHGISEYVGSVEVGKVADLVLWSPAFFGVKPNMIIKGGFIALSQMGDANASIPTPQPVYYREMFAHHGKAKYDANITFVSQAAYDKGIKEELGLERQVLPVKNCRNITKKDMQFNDTTAHIEVNPETYHVFVDGKEVTSKPANKVSLAQLFSIF | 569 |
| 127 | A0A4Y4X1C7 | MKKISRKEYASMYGPTTGDKVRLGDTDLIAEVEHDYTIYGEELKFGGGKTLREGMSQSNNPSKEELDLIITNALIVDYTGIYKADIGIKDGKIAGIGKGGNKDMQDGVKNNLSVGPATEALAGEGLIVTAGGIDTHIHFISPQQIPTAFASGVTTMIGGGTGPADGTNATTITPGRRNLKWMLRAAEEYSMNLGFLAKGNTSNDASLADQIEAGAIGFKIHEDWGTTPSAINHALDVADKYDVQVAIHTDTLNEAGCVEDTMAAIAGRTMHTFHTEGAGGGHAPDIIKVAGEHNILPASTNPTIPFTVNTEAEHMDMLMVCHHLDKSIKEDVQFADSRIRPQTIAAEDTLHDMGIFSITSSDSQAMGRVGEVITRTWQTADKNKKEFGRLKEEKGDNDNFRIKRYLSKYTINPAIAHGISEYVGSVEVGKVADLVLWSPAFFGVKPNMIIKGGFIALSQMGDANASIPTPQPVYYREMFAHHGKAKYDANITFVSQAAYDKGIKEELGLERQVLPVKNCRNITKKDMQFNDTTAHIEVNPETYHVFVDGKEVTSKPANKVSLAQLFSIF | 569 |
| 128 | A0A5M8VBG3 | MKKISRKEYVSMYGPTTGDKVRLGDTDLIAEVEHDYTIYGEELKFGGGKTLREGMSQSNNPSKEELDLIITNALIVDYTGIYKADIGIKDGKIAGIGKGGNKDMQDGVKNNLSVGPATEALAGEGLIVTAGGIDTHIHFISPQQIPTAFASGVTTMIGGGTGPADGTNATTITPGRRNLKWMLRAAEEYSMNLGFLAKGNASNDASLADQIEAGAIGFKIHEDWGTTPSAINHALDVADKYDVQVAIHTDTLNEAGCVEDTMAAIAGRTMHTFHTEGAGGGHAPDIIKVAGEHNILPASTNPTIPFTVNTEAEHMDMLMVCHHLDKSIKEDVQFADSRIRPQTIAAEDTLHDMGIFSITSSDSQAMGRVGEVITRTWQTADKNKKEFGRLKEEKGDNDNFRIKRYLSKYTINPAIAHGISEYVGSVEVGKVADLVLWSPAFFGVKPNMIIKGGFIALSQMGDANASIPTPQPVYYREMFAHHGKAKYDANITFVSQAAYDKGIKEELGLERQVLPVRNCRNITKKDMQFNDTTAHIEVNPETYHVFVDGKEVTSKPANKVSLAQLFSIF | 569 |
| 129 | A0A293V1J2 | MKKISRKEYVSMYGPTTGDKVRLGDTDLIAEVEHDYTIYGEELKFGGGKTLREGMSQSNNPSKEELDLIITNALIVDYTGIYKADIGVKDGKIAGIGKGGNKDMQDGVKNNLSVGPATEALAGEGLIVTAGGIDTHIHFISPQQIPTAFASGVTTMIGGGTGPADGTNATTITPGRRNLKWMLRAAEEYSMNLGFLAKGNASNDASLADQIEAGAIGFKIHEDWGTTPSAINHALDVADKYDVQVAIHTDTLNEAGCVEDTMAAIAGRTMHTFHTEGAGGGHAPDIIKVAGEHNILPASTNPTIPFTVNTEAEHMDMLMVCHHLDKSIKEDVQFADSRIRPQTIAAEDTLHDMGIFSITSSDSQAMGRVGEVITRTWQTADKNKKEFGRLKEEKGDNDNFRIKRYLSKYTINPAIAHGISEYVGSVEVGKVADLVLWSPAFFGVKPNMIIKGGFIALSQMGDANASIPTPQPVYYREMFAHHGKAKYDANITFVSQAAYDKGIKEELGLERQVLPVKNCRNITKKDMQFNDTTAHIEVNPETYHVFVDGKEVTSKPANKVSLAQLFSIF | 569 |
| 130 | A0A496EYR7 | MKKISRKEYVSMYGPTTGDKVRLGDTDLIAEVEHDYTIYGEELKFGGGKTLREGMSQSNNPSKEELDLIITNALIVDYTGIYKADIGIKDGKIAGIGKGGNKDMQDGVKNNLSVGPATEALAGEGLIVTAGGIDTHIHFISPQQIPTAFASGVTTMIGGGTGPADGTNATTITPGRRNLKWMLRAAEEYSMNLGFLAKGNTSNDASLADQIEAGAIGFKIHEDWGTTPSAINHALDVADKYDVQVAIHTDTLNEAGCVEDTMAAIAGRTMHTFHTEGAGGGHAPDIIKVAGEHNILPASTNPTIPFTVNTEAEHMDMLMVCHHLDKSIKEDVQFADSRIRPQTIAAEDTLHDMGIFSITSSDSQAMGRVGEVITRTWQTADKNKKEFGRLKEEKGDNDNFRIKRYLSKYTINPAIAHGISEYVGSVEVGKVADLVLWSPAFFGVKPNMIIKGGFIALSQMGDANASIPTPQPVYYREMFAHHGKAKYDANITFVSQVAYDKGIKEELGLERQVLPVKNCRNITKKDMQFNDTTAHIEVNPETYHVFVDGKEVTSKPANKVSLAQLFSIF | 569 |
| 131 | A0A6I4C915 | MKKISRKEYVSMYGPTTGDKVRLGDTDLIAEVEHDYTIYGEELKFGGGKTLREGMSQSNNPSKEELDLIITNALIVDYTGIYKADIGIKDGKIAGIGKGGNKDMQDGVKNNLSVGPATEALAGEGLIVTAGGIDTHIHFISPQQIPTAFASGVTTMIGGGTGPADGTNATTITPGRRNLKWMLRAAEEYSMNLGFLAKGNTSNDASLADQIEAGAIGFKIHEDWGTTPSAINHALDVADKYDVQVAIHTDTLNEAGCVEDTMAAIAGRTMHTFHTEGAGGGHAPDIIKVAGEHNILPASTNPTIPFTVNTEAEHMDMLMVCHHLDKSIKEDVQFADSRIRPQTIAAEDTLHDMGIFSITSSDSQAMGRVGEVITRTWQTADKNKKEFGRLKEEKGDNDNFRIKRYLSKYTINPAIAHGISEYVGSVEVGKVADLVLWSPAFFGVKPNMIIKGGFIALSQMGDANASIPTPQPVYYREMFAHHGKAKYDANITFVSQAAYNKGIKEELGLERQVLPVKNCRNITKKDMQFNDTTAHIEVNPETYHVFVDGKEVTSKPANKVSLAQLFSIF | 569 |
| 132 | A0A6J4CZ13 | MKKISRKEYVSMYGPTTGDKVRLGDTDLILEVEHDCTTYGEEIKFGGGKTIRDGMGQTNSPSSHELDLVITNALIVDYTGIYKADIGIKDGKIHGIGKAGNKDIQDGVCNRLCVGPATEALAGEGLIVTAGGIDTHIHFISPQQIPTAFASGITTMLGGGTGPADGTNATTITPGRWNLKEMLRASEEYAMNLGYMGKGNVSYEPSLVEQLEAGAIGFKIHEDWGSTPSAIHHALKIADE  YDVQVAIHTDTLNEAGCVEDTLEAIAGRTIHTFHTEGAGGGHAPDVIKMAGAFNVLPASTNPTIPFTKNTEAEHMDMLMVCHHLDKNIKEDVEFADSRIRPQTIAAEDKLHDMGIFSITSSDSQAMGRVGEVITRTWQTADKNKKEFGRLKEETGDNDNFRIKRYISKYTINPAIAHGISEYVGSVEVGKYADLVLWSPAFFGIKPNMIIKGGMIALSQMGDANASIPTPQPVYYREMFGHHGKAKFDTNITFVSRVAYENGIKHELGLQRKVLPVKNCRNITKKDLKFNDVTAHIEVNPETYKVKVDGQEVTSKAADKISLAQLYNLF | 569 |
| 133 | A0A1Q2R9X3 | MKKISRKEYASMYGPTTGDKVRLGDTDLIAEVEHDYTIYGEELKFGGGKTLREGMSQSNNPSKEELDLIITNALIVDYTGIYKADIGIKDGKIAGIGKGGNKDTQDGVKNNLSVGPATEALAGEGLIVTAGGIDTHIHFISPQQIPTAFASGVTTMIGGGTGPADGTNATTITPGRRNLKWMLRAAEEYSMNLGFLAKGNTSNDASLADQIEAGAIGFKIHEDWGTTPSAINHALDVADKYDVQVAIHTDTLNEAGCVEDTMAAIAGRTMHTFHTEGAGGGHAPDIIKVAGEHNILPASTNPTIPFTVNTEAEHMDMLMVCHHLDKNIKEDVQFADSRIRPQTIAAEDTLHDMGIFSITSSDSQAMGRVGEVITRTWQTADKNKKEFGRLKEEKGDNDNFRIKRYLSKYTINPAIAHGISEYVGSVEVGKVADLVLWSPAFFGVKPNMIIKGGFIALSQMGDANASIPTPQPVYYREMFAHHGKAKYDANITFVSQAAYDKGIKEELGLERQVLPVKNCRNITKKDMQFNDTTAHIEVNPETYHVFVDGKEVTSKPANKVSLAQLFSIF | 569 |
| 134 | A0A2A6S0A0 | MKKINRKEYVSMYGPTTGDKVRLGDTDLIAEVEHDYTIYGEELKFGGGKTLREGMSQSNNPSKEELDLIITNALIVDYTGIYKADIGIKDGKIAGIGKGGNKDMQDGVKNNLSVGPATEALAGEGLIVTAGGIDTHIHFISPQQIPTAFASGVTTMIGGGTGPADGTNATTITPGRRNLKWMLRAAEEYSMNLGFLAKGNASNDASLADQIEAGAIGFKIHEDWGTTPSAINHALDVADKYDVQVAIHTDTLNEAGCVEDTMAAIAGRTMHTFHTEGAGGGHAPDIIKVAGEHNILPASTNPTIPFTVNTEAEHMDMLMVCHHLDKSIKEDVQFADSRIRPQTIAAEDTLHDMGIFSITSSDSQAMGRVGEVITRTWQTADKNKKEFGRLKEEKGDNDNFRIKRYLSKYTINPAIAHGISEYVGSVEVGKVADLVLWSPAFFGVKPNMIIKGGFIALSQMGDANASIPTPQPVYYREMFAHHGKAKYDANITFVSQAAYDKGIKEELGLERQVLPVKNCRNITKKDMQFNDTTAHIEVNPETYHVFVDGKEVTSKPANKVSLAQLFSIF | 569 |
| 135 | A0A2T6NY60 | MKKISRKEYVSMYGPTTGDKVRLGDTDLIAEVEHDYTIYGEELKFGGGKTLREGMSQSNNPSKEELDLIITNALIVDYTGIYKADIGIKDGKIAGIGKGGNKDTQDGVKNNLSVGPATEALAGEGLIVTAGGIDTHIHFISPQQIPTAFASGVTTMIGGGTGPADGTNATTITPGRRNLKFMLRAAEEYSMNFGFLAKGNASNDASLADQIEAGAIGFKIHEDWGTTPSAINHALDVADKYDVQVAIHTDTLNEAGCVEDTMAAIAGRTMHTYHTEGAGGGHAPDIIKVAGEHNILPASTNPTIPFTVNTEAEHMDMLMVCHHLDKSIKEDVQFADSRIRPQTIAAEDTLHDMGIFSITSSDSQAMGRVGEVITRTWQTADKNKKEFGRLKEEKGDNDNFRIKRYLSKYTINPAIAHGISEYVGSVEVGKVADLVLWSPAFFGVKPNMIIKGGFIALSQMGDANASIPTPQPVYYREMFAHHGKAKYDANITFVSQAAYDKGIKEELGLERQVLPVKNCRNITKKDMQFNDTTAHIEVNSETYHVFVDGKEVTSKPANKVSLAQLFSIF | 569 |
| 136 | A0A7K1LU75 | MKKISRKEYVSMYGPTTGDKVRLGDTDLIAEVEHDYTIYGEELKFGGGKTLREGMSQSNNPSKEELDLIITNALIVDYTGIYKADIGIKDGKIVGIGKGGNKDMQDGVKNNLSVGPATEALAGEGLIVTAGGIDTHIHFISPQQIPTAFASGVTTMIGGGTGPADGTNATTITPGRRNLKWMLRAAEEYSMNLGFLAKGNASNDASLADQIEAGAIGFKIHEDWGTTPSAINHALDVADKYDVQVAIHTDTLNEAGCVEDTMAAIAGRTMHTFHTEGAGGGHAPDIIKVAGEHNILPASTNPTIPFTVNTEAEHMDMLMVCHHLDKSIKEDVQFADSRIRPQTIAAEDTLHDMGIFSITSSDSQAMGRVGEVITRTWQTADKNKKEFGRLKEEKGDNDNFRIKRYLSKYTINPAIAHGISEYVGSVEVGKVADLVLWSPAFFGVKPNMIIKGGFIALSQMGDANASIPTPQPVYYREMFAHHGKAKYDANITFVSQAAYDKGIKEELGLERQVLPVKNCRNITKKDMQFNDTTAHIEVNPETYHVFVDGKEVTSKPANKVSLAQLFSIF | 569 |
| 137 | A0A1Q2PHU0 | MKKISRKEYASMYGPTTGDKVRLGDTDLIAEVEHDYTIYGEELKFGGGKTLREGMSQSNNPSKEELDLIITNALIVDYTGIYKADIGIKDGKIAGIGKGGNKDMQDGVKNNLSVGPATEALAGEGLIVTAGGIDTHIHFISPQQIPTAFASGVTTMIGGGTGPADGTNATTITPGRRNLKWMLRAAEEYSMNLGFLAKGNASNDASLADQIEAGAIGLKIHEDWGTTPSAINHALDVADKYDVQVAIHTDTLNEAGCVEDTMAAIAGRTMHTFHTEGAGGGHAPDIIKVAGEHNILPASTNPTIPFTVNTEAEHMDMLMVCHHLDKSIKEDVQFADSRIRPQTIAAEDTLHDMGIFSITSSDSQAMGRVGEVITRTWQTADKNKKEFGRLKEEKGDNDNFRIKRYLSKYTINPAIAHGISEYVGSVEVGKVADLVLWSPAFFGVKPNMIIKGGFIALSQMGDANASIPTPQPVYYREMFAHHGKAKYDANITFVSQAAYDKGIKEELGLERQVLPVKNCRNITKKDMQFNDTTAHIEVNPETYHVFVDGKEVTSKPATKVSLAQLFSIF | 569 |
| 138 | A0A1V3A7M7 | MKKISRKEYVSMYGPTTGDKVRLGDTDLIAEVEHDYTIYGEELKFGGGKTLREGMSQSNNPSKEELDLIITNALIVDYTGIYKADIGIKDGKIAGIGKGGNKDMQDGVKNNLSVGPATEALAGEGLIVTAGGIDTHIHFISPQQIPTAFASGVTTMIGGGTGPADGTNATTITPGRRNLKWMLRAAEEYSMNLGFLAKGNASNDASLADQIEAGAIGFKIHEDWGTTPSAINHALDVADKYDVQVAIHTDTLNEAGCVEDTMAAIAGRTMHTFHTEGAGGGHAPDIIKVAGEHNILPASTNPTIPFTVNTEAEHMDMLMVCHHLDKSIKEDVQFADSRIRPQTIAAEDTLHDMGIFSITSSDSQAMGRVGEVITRTWQTADKNKKEFGRLKEEKGDNDNFRIKRYLSKYTINPAIAHGISEYVGSVEVGKVADLVLWSPAFFGVKPNMIIKGGFIALSQMGDANASIPTPQPVYYREMFAHHGKAKYDANITFVSQAAYDKGIKEELGLERQVLPVKNCRNITKKDMQFNDTTAHIEVNPETYRVFVDGKEVTSKPANKVSLAQLFSIF | 569 |
| 139 | A0A3S5BYW5 | MKKISRKEYVSMYGPTTGDKVRLGDTDLIAEVEHDYTIYGEELKFGGGKTLREGMSQSNNPSKEELDLIITNALIVDYTGIYKADIGIKDGKIAGIGKGGNKDTQDGVKNNLSVGPATEALAGEGLIVTAGGIDTHIHFISPQQIPTAFASGVTTMIGGGTGPADGTNATTITPGRRNLKFMLRAAEEYSMNFGFLAKGNASNDASLADQIEAGAIGLKIHEDWGTTPSAINHALDVADKYDVQVAIHTDTLNEAGCVEDTMAAIAGRTMHTYHTEGAGGGHAPDIIKVAGEHNILPASTNPTIPFTVNTEAEHMDMLMVCHHLDKSIKEDVQFADSRIRPQTIAAEDTLHDMGIFSITSSDSQAMGRVGEVITRTWQTADKNKKEFGRLKEEKGDNDNFRIKRYLSKYTINPAIAHGISEYVGSVEVGKVADLVLWSPAFFGVKPNMIIKGGFIALSQMGDANASIPTPQPVYYREMFAHHGKAKYDANITFVSQAAYDKGIKEELGLERQVLPVKNCRNITKKDMQFNDTTAHIEVNSETYHVFVDGKEVTSKPANKVSLAQLFSIF | 569 |
| 140 | A0A402DX05 | MKKISRKEYASMYGPTTGDKVRLGDTDLIAEVEHDYTIYGEELKFGGGKTLREGMSQSNNPSKEELDLIITNALIVDYTGIYKADIGIKDGKIAGIGKGGNKDMQDGVKNNLSVGPATEALAGEGLIVTAGGIDTHIHFISPQQIPTAFASGVTTMIGGGTGPADGTNATTITPGRRNLKWMLRAAEEYSMNLGFLGKGNASNDESLADQIEAGAIGLKIHEDWGTTPSAINHALDVADKYDVQVAIHTDTLNEAGCVEDTMAAIAGRTMHTFHTEGAGGGHAPDIIKVAGEHNILPASTNPTIPFTVNTEAEHMDMLMVCHHLDKSIKEDVQFADSRIRPQTIAAEDTLHDMGIFSITSSDSQAMGRVGEVITRTWQTADKNKKEFGRLKEEKGDNDNFRIKRYLSKYTINPAIAHGISEYVGSVEVGKVADLVLWSPAFFGVKPNMIIKGGFIALSQMGDANASIPTPQPVYYREMFAHHGKAKYDANITFVSQAAYDKGIKEELGLERQVLPVKNCRNITKKDMQFNDTTAHIEVNPETYHVFVDGKEVTSKPATKVSLAQLFSIF | 569 |

**Table S2.Primer sequences**

| purpose | Genes | primer | | length (bp) |
| --- | --- | --- | --- | --- |
| Primers of detecting drug-resistant mutations | rdxA | F | ATCAATGAAATTTTTGGATC | 254 |
|  |  | R | TACTACAAATTAGCAGCATTC |  |
|  | gyrA | F | GCAAAATATCATGCGTG | 1534 |
|  |  | R | GTGCATAGGCGTATTTT |  |
|  | UreB | F | TCATGGCGCTAAAAGCG | 1825 |
|  |  | r | ACTAAGGATTTAAGGAGG |  |
| Primers of qRT-PCR | 00042 | F | CTCTTGGAGTTTTAAATTTTC | 174 |
|  |  | R | TACCAAGCGAGCAAATTAG |  |
|  | SotB | F | GCCAAACAACGCACCTGATC | 298 |
|  |  | R | TGGGTTAGCGGGCGTGGCGG |  |
|  | CusB | F | AGCGCTCAAAGCGAGCTGTT | 167 |
|  |  | R | ATAACGCCGTTGAAGTGAGAG |  |
|  | MsbA | F | GTCAAGCCCACTTTAGATG | 131 |
|  |  | R | AAGTTAGTGAAATAAGTGCC |  |
|  | 01163 | F | GCCGTTTGAATGAGCAACGCTCC | 124 |
|  |  | R | CTTACCACTAAAAGGGTTCTGGG |  |
|  | CorC | F | CCCAAGCGTTACCTGCTCGCATTC | 205 |
|  |  | R | CGGTGGCACAGCCGGGTTGCTCAC |  |
|  | 00800 | F | CCACTTCTGCATCAAAGCGCG | 206 |
|  |  | R | AGCGTATAAAAAATTGGAG |  |
|  | KefC | F | GGATAAGGAATTGGTGGATG | 189 |
|  |  | R | GAGACTCTAAAACTGCCATA |  |
|  | CusA | F | GCAGCCAATCTCAATGGCGAT | 160 |
|  |  | R | CGCTCCTGTCATACACGCTGG |  |
|  | 00466 | F | CCTTACAAGAGCAAATTGACGC | 203 |
|  |  | R | GATCATTTGAGATTGCGTGAG |  |
|  | 16S RNA | F | TCCTGGCTCAGAGTGAACG | 136 |
|  |  | R | GCGCCACTAATCAGCACTC |  |
| Knockout of the UreB | UreB | F1 | ATGACAACCACATTACAAACAATGGAAAGCCCGCC | 375 |
|  |  | R1 | GAATTGTTTTAGGATTTTCTCTAGGATTTTTAAT |  |
|  | Kana | F2 | CCTAGAGAAAATCCTAAAACAATTCATCCAGTAAAATATAATA | 1314 |
|  |  | R2 | CAATTAAGGAGTAAGAAGGTGATAGGTAAGATTATACCGAGGTA |  |
|  | UreB | F3 | AATCTTACCTATCACCTTCTTACTCCTTAATTGTTTTTACAT | 434 |
|  |  | R3 | GCCAATGGTAAATTAGTTCCTGGTGAGTTG |  |
| point mutation of the UreB | UreB-D134 | F | TGGATGTGTGTGGCAATACCACCAGCAGTTACGATC |  |
|  |  | R | CTGGTGGTATTGCCACACACATCCACTTCATCTCCCCCCAAC |  |
|  | UreB-T135 | F | TGGATGTGTGCGTCAATACCACCAGCAGTTACGATC |  |
|  |  | R | CTGGTGGTATTGACGCACACATCCACTTCATCTCCCCCCAAC |  |
|  | UreB-H136 | F | TGGATGGCTGTGTCAATACCACCAGCAGTTACGATC |  |
|  |  | R | CTGGTGGTATTGACACAGCCATCCACTTCATCTCCCCCCAAC |  |
|  | UreB-I137 | F | TGGGCGTGTGTGTCAATACCACCAGCAGTTACGATC |  |
|  |  | R | CTGGTGGTATTGACACACACGCCCACTTCATCTCCCCCCAAC |  |
|  | UreB-H138 | F | GAGATGAAGGCATGTGTGTGTCAATACCACCAGCAGTTA |  |
|  |  | R | CTGGTGGTATTGACACACACATCGCCTTCATCTCCCCCCAAC |  |
|  | UreB-F139 | F | GAGATGGCGTGGATGTGTGTGTCAATACCACCAGCAGTTA |  |
|  |  | R | CTGGTGGTATTGACACACACATCCACGCCATCTCCC |  |
|  | UreB-I140 | F | TTGGGGGGAGGCGAAGTGGATGTGTGTGTCAATACCACCAGCAGTTA |  |
|  |  | R | ACTGCTGGTGGTATTGACACACACATCCACTTCGCCTCCCCCCAAC |  |
|  | UreB-I157 | F | TTAGTGCCATCAGCAGGGCCAGTTCCGCCACCTGCCATGGTTGTTAC |  |
|  |  | R | CTACAGCTTTTGCAAGCGGTGTAACAACCATGGCAGGTGGCGG |  |
|  | UreB-G158 | F | TTAGTGCCATCAGCAGGGCCAGTTCCGCCTGCAATCATGGTTGTTAC |  |
|  |  | R | ACAGCTTTTGCAAGCGGTGTAACAACCATGATTGCAGGCGGAACTGGC |  |
|  | UreB-G159 | F | TTAGTGCCATCAGCAGGGCCAGTTCCGGCACCAATCATGGTTGTTAC |  |
|  |  | R | ACAGCTTTTGCAAGCGGTGTAACAACCATGATTGGTGCCGGAACTGGC |  |
|  | UreB-G166 | F | CTGGAGTGATAGTGGTTGCGTTAGTGGCATCAGCAGGGCCAGTTCCGCC |  |
|  |  | R | ATGATTGGTGGCGGAACTGGCCCTGCTGATGCCACTAACGCA |  |
|  | UreB-T167 | F | CTGGAGTGATAGTGGTTGCGTTTGCGCCATCAGCAGGGCCAGTTCCGCC |  |
|  |  | R | GGTGGCGGAACTGGCCCTGCTGATGGCGCAAACGCAACCACTATCACTCC |  |
|  | UreB-N168 | F | CTGGAGTGATAGTGGTTGCGGCAGTGCCATCAGCAGGGCCAGTTCCGCC |  |
|  |  | R | GGTGGCGGAACTGGCCCTGCTGATGGCACTGCCGCAACCACTATCACTCC |  |
|  | UreB-A169 | F | CTGGAGTGATAGTGGTTTAGTTAGTGCCATCAGCAGGGCCAGTTCCGCC |  |
|  |  | R | GGTGGCGGAACTGGCCCTGCTGATGGCACTAACAATACCACTATCACTCC |  |
|  | UreB-T170 | F | CTGGAGTGATAGTGGCTGCGTTAGTGCCATCAGCAGGGCCAGTTCCGCC |  |
|  |  | R | GGTGGCGGAACTGGCCCTGCTGATGGCACTAACGCAGCCACTATCACTCC |  |
|  | UreB-T171 | F | CTGGAGTGATTGCGGTTGCGTTAGTGCCATCAGCAGGGCCAGTTCCGCC |  |
|  |  | R | GGTGGCGGAACTGGCCCTGCTGATGGCACTAACGCAACCGCAATCACTCC |  |
|  | UreB-1172 | F | CTGCCTGGAGTGGCAGTGGTTGCGTTAGTGCCATCAGCAGGGCCAGTT |  |
|  |  | R | CGGAACTGGCCCTGCTGATGGCACTAACGCAACCACTGCCACTCCAGGCAG |  |
|  | UreB-L196 | F | CGGCTAAGCTCGCATCGTTAGAAACGTTACCTTTAGCCGCGAAACCAAAG |  |
|  |  | R | GCTGAAGAATATTCTATGAACTTTGGTTTCGCGGCTAAAGGTAACG |  |
|  | UreB-K219 | F | CAGAAGGAGTGGTACCCCAGTCTTCGTGGATTGCAAAGCCAATCGCA |  |
|  |  | R | AAATTGAAGCTGGTGCGATTGGCTTTGCAATCCACGAAGACTGG |  |
|  | UreB-I220 | F | CAGAAGGAGTGGTACCCCAGTCTTCGTGGGCTTTAAAGCCAATCGCA |  |
|  |  | R | AAATTGAAGCTGGTGCGATTGGCTTTAAAGCCCACGAAGACTGG |  |
|  | UreB-H221 | F | CAGAAGGAGTGGTACCCCAGTCTTCGGCGATTTTAAAGCCAATCGCA |  |
|  |  | R | GAAGCTGGTGCGATTGGCTTTAAAATCGCCGAAGACTGGGGTACCAC |  |
|  | UreB-E222 | F | ATGATTGATTGCAGAAGGAGTGGTACCCCAGTCTGCGTGGATTTTAAAGCCAAT |  |
|  |  | R | GAAGCTGGTGCGATTGGCTTTAAAATCCACGCAGACTGGGGTACCAC |  |
|  | UreB-D223 | F | ATGATTGATTGCAGAAGGAGTGGTACCCCAGGCTTCGTGGATTTTAAAGCCAAT |  |
|  |  | R | GAAGCTGGTGCGATTGGCTTTAAAATCCACGAAGCCTGGGGTACCAC |  |
|  | UreB-T226 | F | TGCAACATCTAACGCATGATTGATTGCAGAAGGAGTGGCACCCCAGTCTTCGTGGA |  |
|  |  | R | GCGATTGGCTTTAAAATCCACGAAGACTGGGGTGCCACTCCTTCTGCAATCA |  |
|  | UreB-A246 | F | AACCGGCTTCATTCAAAGTGTCTGTGTGGATTTAGACTTGC |  |
|  |  | R | GCGTTAGATGTTGCAGACAAATACGATGTGCAAGTCAATATCCACACAGACA |  |
|  | UreB-I247 | F | CAACCGGCTTCATTCAAAGTGTCTGTGTGGGCAGCGACTTGCACA |  |
|  |  | R | TGTTGCAGACAAATACGATGTGCAAGTCGCTGCCCACACAGACACTT |  |
|  | UreB-H248 | F | CAACCGGCTTCATTCAAAGTGTCTGTGGCGATAGCGACTTGCACA |  |
|  |  | R | TGTTGCAGACAAATACGATGTGCAAGTCGCTATCGCCACAGACACTT |  |
|  | UreB-T249 | F | CAACCGGCTTCATTCAAAGTGTCTGCGTGGATAGCGACTTGCACA |  |
|  |  | R | TGTTGCAGACAAATACGATGTGCAAGTCGCTATCCACGCAGACACTT |  |
|  | UreB-D250 | F | CTTCCACGCAACCGGCTTCATTCAAAGTGGCTGTGTGGATAGCG |  |
|  |  | R | GACAAATACGATGTGCAAGTCGCTATCCACACAGCCACTTTGAATGA |  |
|  | UreB-T251 | F | CTGCCATAGTGTCTTCCACGCAACCGGCTTCATTCAATGCGTCTGTGTGGATA |  |
|  |  | R | CAAATACGATGTGCAAGTCGCTATCCACACAGACGCATTGAATGAAGCCG |  |
|  | UreB-H271 | F | GTGTCCGCCGCCAGCGCCTTCAGTGTGGAAAGTGGCCATAGTGCG |  |
|  |  | R | GGCAGCCATTGCCGGACGCACTATGGCCACTTTCCACACTGAAGGC |  |
|  | UreB-T272 | F | GTGTCCGCCGCCAGCGCCTTCAGTGTGGAATGCGTGCATAGTGCG |  |
|  |  | R | GGCAGCCATTGCCGGACGCACTATGCACGCATTCCACACTGAAGGC |  |
|  | UreB-F273 | F | AGGAGCGTGTCCGCCGCCAGCGCCTTCAGTGTGGGCAGTGTGCATAG |  |
|  |  | R | AGCCATTGCCGGACGCACTATGCACACTGCCCACACTGAAGGCGCTGGC |  |
|  | UreB-H274 | F | AGGAGCGTGTCCGCCGCCAGCGCCTTCAGTGGCGAAAGTGTGCATAG |  |
|  |  | R | AGCCATTGCCGGACGCACTATGCACACTTTCGCCACTGAAGGCGCTGGC |  |
|  | UreB-T275 | F | AGGAGCGTGTCCGCCGCCAGCGCCTTCGGCGTGGAAAGTGTGCATAG |  |
|  |  | R | AGCCATTGCCGGACGCACTATGCACACTTTCCACGCCGAAGGCGCTGGC |  |
|  | UreB-E276 | F | AATAATATCAGGAGCGTGTCCGCCGCCAGCGCCTGCAGTGTGGAAAGTGTGCA |  |
|  |  | R | TTGCCGGACGCACTATGCACACTTTCCACACTGCAGGCGCTGGCGGCGGACAC |  |
|  | UreB-G277 | F | AATAATATCAGGAGCGTGTCCGCCGCCAGCGGCTTCAGTGTGGAAAGTGTGCA |  |
|  |  | R | TTGCCGGACGCACTATGCACACTTTCCACACTGAAGCCGCTGGCGGCGGACAC |  |
|  | UreB-A278 | F | AATAATATCAGGAGCGTGTCCGCCGCCTTAGCCTTCAGTGTGGAAAGTGTGCA |  |
|  |  | R | TTGCCGGACGCACTATGCACACTTTCCACACTGAAGGCAATGGCGGCGGACAC |  |
|  | UreB-G279 | F | GCCACTTTAATAATATCAGGAGCGTGTCCGCCGGCAGCGCCTTCAGTGTG |  |
|  |  | R | ACTATGCACACTTTCCACACTGAAGGCGCTGCCGGCGGACACGCTCCTGAT |  |
|  | UreB-G280 | F | GCCACTTTAATAATATCAGGAGCGTGTCCGGCGCCAGCGCCTTCAGTGTG |  |
|  |  | R | ACTATGCACACTTTCCACACTGAAGGCGCTGGCGCCGGACACGCTCCTGAT |  |
|  | UreB-G281 | F | GCCACTTTAATAATATCAGGAGCGTGTGCGCCGCCAGCGCCTTCAGTGTG |  |
|  |  | R | ACTATGCACACTTTCCACACTGAAGGCGCTGGCGGCGCACACGCTCCTGAT |  |
|  | UreB-I286 | F | GCAGGTAGAATGTTGTGTTCACCGGCCACTTTAATGGCATCAGGAG |  |
|  |  | R | ACTGAAGGCGCTGGCGGCGGACACGCTCCTGATGCCATTAAAGTGGCCGGT |  |
|  | UreB-A298 | F | TCACGGTGAAAGGGATAGTGGGGTTAGTGGATTAAGGTAGAATGTTG |  |
|  |  | R | TAAAGTGGCCGGTGAACACAACATTCTACCTAATTCCACTAACCCCACTATCCC |  |
|  | UreB-S299 | F | TCACGGTGAAAGGGATAGTGGGGTTAGTGGCAGCAGGTAGAATGTTG |  |
|  |  | R | TAAAGTGGCCGGTGAACACAACATTCTACCTGCTGCCACTAACCCCACTATCCC |  |
|  | UreB-T300 | F | GGCTTCTGTATTCACGGTGAAAGGGATAGTGGGGTTGGCGGAAGCAGGTAGAA |  |
|  |  | R | TAAAGTGGCCGGTGAACACAACATTCTACCTGCTTCCGCCAACCCCACTATCCC |  |
|  | UreB-N301 | F | GGCTTCTGTATTCACGGTGAAAGGGATAGTGGGGGCAGTGGAAGCAGGTAGAA |  |
|  |  | R | TAAAGTGGCCGGTGAACACAACATTCTACCTGCTTCCACTGCCCCCACTATCCC |  |
|  | UreB-P302 | F | GGCTTCTGTATTCACGGTGAAAGGGATAGTGGCGTTAGTGGAAGCAGGTAGAA |  |
|  |  | R | CGGTGAACACAACATTCTACCTGCTTCCACTAACGCCACTATCCCTTTCACC |  |
|  | UreB-H322 | F | GAACATCTTCTTTAATGCTTTTATCCAAGTGGGCGCACACCATAAGC |  |
|  |  | R | CCGAACACATGGACATGCTTATGGTGTGCGCCCACTTGGATAAAAGC |  |
|  | UreB-R338 | F | ATGCAAAGTGTCTTCAGCCGCAATGGTTTGAGGGCGGATGGCTGAATCAGCG |  |
|  |  | R | AAAAGCATTAAAGAAGATGTTCAGTTCGCTGATTCAGCCATCCGCCCTCAAAC |  |
|  | UreB-S360 | F | CACCCACACGACCCATAGCTTGAGAGTCAGAGGCGGTGATTGAGA |  |
|  |  | R | CTTTGCATGACATGGGGATTTTCTCAATCACCGCCTCTGACTCTCAAG |  |
|  | UreB-S361 | F | ATAACTTCACCCACACGACCCATAGCTTGAGAGTCCGGACTGGTGATTGAGAA |  |
|  |  | R | GACATGGGGATTTTCTCAATCACCAGTGCCGACTCTCAAGCTATGGGTCGT |  |
|  | UreB-D362 | F | ATAACTTCACCCACACGACCCATAGCTTGAGAGGCAGAACTGGTGATTGAGAA |  |
|  |  | R | GACATGGGGATTTTCTCAATCACCAGTTCTGCCTCTCAAGCTATGGGTCGT |  |
|  | UreB-S363 | F | ATAACTTCACCCACACGACCCATAGCTTGGGCGTCAGAACTGGTGATTGAGAA |  |
|  |  | R | GACATGGGGATTTTCTCAATCACCAGTTCTGACGCCCAAGCTATGGGTCGT |  |
|  | UreB-Q364 | F | CCAAGTTCTGGTGATAACTTCACCCACACGACCCATAGCTGCAGAGTCAGAACTGG |  |
|  |  | R | ACATGGGGATTTTCTCAATCACCAGTTCTGACTCTGCAGCTATGGGTCGTGTGG |  |
|  | UreB-A365 | F | CCAAGTTCTGGTGATAACTTCACCCACACGACCCATTTATTGAGAGTCAGAACTGG |  |
|  |  | R | ACATGGGGATTTTCTCAATCACCAGTTCTGACTCTCAAAATATGGGTCGTGTGG |  |
|  | UreB-M366 | F | CCAAGTTCTGGTGATAACTTCACCCACACGACCTGCAGCTTGAGAGTCAGAACTGG |  |
|  |  | R | ACATGGGGATTTTCTCAATCACCAGTTCTGACTCTCAAGCTGCAGGTCGTGTGG |  |
|  | UreB-G367 | F | CTGTTTGCCAAGTTCTGGTGATAACTTCACCCACACGGGCCATAGCTTGAGAGT |  |
|  |  | R | GGATTTTCTCAATCACCAGTTCTGACTCTCAAGCTATGGCCCGTGTGGGTGAA |  |
|  | UreB-R368 | F | CTGTTTGCCAAGTTCTGGTGATAACTTCACCCACTGCACCCATAGCTTGAGAGT |  |
|  |  | R | GGATTTTCTCAATCACCAGTTCTGACTCTCAAGCTATGGGTGCAGTGGGTGAA |  |
|  | Homology arm | F | AATGCGCCAAAAGGAGCTTTTTAAAAGCCATGCCATAGAGTTTGGCATGGTG |  |
|  |  | R | CATACCTCGGTATAATCTTATGGTGGCCACGACTGGACTTGAACCAGCGGCCAC |  |
|  | Kana | F | GTCCAGTCGTGGCCACCATAAGATTATACCGAGGTATGAAAACGAGAATTGG |  |
|  |  | R | GAGAATTAAAATTGGAGTGATAACTAAAACAATTCATCCAGTAAAATATAATA |  |
|  | His | F | CATCATCATCATCATCATTAAGAAATGAAAAAGATTAGCAGAAAAGAATATG |  |
|  |  | R | ATGATGATGATGATGATGCTCCTTAATTGTTTTTACATAGTTGTCATCGC |  |
|  | Homology arm2 | F | GCATTTTCCATCATCATCATCATCATTAGGATTTTCTCTAGGATTTTTAATTTTCTAGG |  |
|  |  | R | CAATCCCAACATATAACAATACAAGTCCTAGCATGCCTTTTCCTTCC |  |

**Table S3. Construction of phage mutation library.**

| Purpose | Genes | Sequence | length (aa/bp) |
| --- | --- | --- | --- |
| Gene synthesis template amino acid sequence | UreB | EALAGEGLIVTAGGIDTHIHFISPQQIPTAFASGVTTMIGGGTGPADGTNATTITPGRRNLKFMLRAAEEYSMNFGFLAKGNVSNDASLADQIEAGAIGFKIHEDWGTTPSAINHALDVADKYDVQVAIHTDTLNEAGCVEDTMAAIAGRTMHTFHTEGAGGGHAPDIIKVAGEHNILPASTNPTIPFTVNTEAEHMDMLMVCHHLDKSIKEDVQFADSRIRPQTIAAEDTLHDMGIFSITSSDSQAMGRVGEVITRT WQTADKNKKEFGR | 271aa |
| Gene synthesis template DNA sequence | UreB | GCTACCGTGGCCCAGGCGGCCGAAGCCTTAGCCGGTGAAGGTTTGATCGTAACTGCTGGTGGTATTGACACANNKNNKNNKTTCATCTCCCCCCAACAAATCCCTACAGCTTTTGCAAGCGGTGTAACAACCATGATTGGTGGCGGAACTGGCCCTGCTGATGGCACTNNKNNKNNKNNKATCACTCCAGGCAGAAGAAATTTAAAATTCATGCTCAGAGCGGCTGAAGAATATTCTATGAACTTTGGTTTCTTGGCTAAAGGTAACGTTTCTAACGATGCGAGCTTAGCCGATCAAATTGAAGCTGGTGCGATTGGCTTTNNKNNKNNKNNKNNKTGGGGTACCACTCCTTCTGCAATCAATCATGCGTTAGATGTTGCAGACAAATACGATGTGCAAGTCGCTNNKNNKNNKNNKNNKTTGAATGAAGCCGGTTGCGTGGAAGACACTATGGCAGCCATTGCCGGACGCACTATGCACACTNNKNNKACTGAAGGCGCTNNKNNKGGACACGCTCCTGATATTATTAAAGTGGCCGGTGAACACAACATTCTACCTNNKNNKNNKAACCCCACTATCCCTTTCACCGTGAATACAGAAGCCGAACACATGGACNNKNNKATGNNKNNKNNKCACTTGGATAAAAGCATTAAAGAAGATGTTCAGTTCGCTGATTCANNKATCCGCCCTCAAACCATTGCGGCTGAAGACACTTTGCATGACATGGGGATTTTCTCAATCACCNNKNNKNNKNNKNNKNNKNNKGGTCGTGTGGGTGAAGTTATCACCAGAACTTGGCAAACAGCTGACAAAAACAAAAAAGAATTTGGCCGCGGCCAGGCCGGCCAGCACCAT | 855bp |
| Purpose | Primer name | Primer | Site |
| NNK primers for gene synthesis | UreB-2F | GCTACCGTGGCCCAGGCGGCCGAAGCCT | N-terminal |
|  | UreB-2R1 | GATTTGTTGGGGGGAGATGAAMNNMNNMNNTGTGTCAATACCACCAGCAG | H136, I137, H138 |
|  | UreB-2F2 | TTCATCTCCCCCCAACAAATC |  |
|  | UreB-2R2 | ATTTCTTCTGCCTGGAGTGATMNNMNNMNNMNNAGTGCCATCAGCAGGGC CAG | N168, A169, T170, T171 |
|  | UreB-2F3 | ATCACTCCAGGCAGAAGAAAT |  |
|  | UreB-2R3 | GCAGAAGGAGTGGTACCCCAMNNMNNMNNMNNMNNAAAGCCAATCGCA CCAGCTT | K219, I220, H221, E222, D223 |
|  | UreB-2R3P | CTGCAACATCTAACGCATGATTGATTGCAGAAGGAGTGGTACCCCA |  |
|  | UreB-2F4P | AATCATGCGTTAGATGTTGCAGACAAATACGATGTGCAAGTCGCT |  |
|  | UreB-2F4 | AAATACGATGTGCAAGTCGCTNNKNNKNNKNNKNNKTTGAATGAAGCCGGTT GCGT | I247, H248, T249, D250, T251 |
|  | UreB-2R4 | AGTGTGCATAGTGCGTCCGGC |  |
|  | UreB-2F5 | GCCGGACGCACTATGCACACTNNKNNKACTGAAGGCGCTNNKNNKGGACAC GCTCCTGATATTAT | F273, H274, G279, G280 |
|  | UreB-2R5 | AGGTAGAATGTTGTGTTCACC |  |
|  | UreB-2F6 | GGTGAACACAACATTCTACCTNNKNNKNNKAACCCCACTATCCCTTTCACCGT GAATAC | A298, S299,  T300 |
|  | UreB-2R6 | GTCCATGTGTTCGGCTTCTGTATTCACGGTGAAAGGGATAGT |  |
|  | UreB-2R6P | GTCCATGTGTTCGGCTTCTG |  |
|  | UreB-2F7P | CAGAAGCCGAACACATGGAC |  |
|  | UreB-2F7 | CAGAAGCCGAACACATGGACNNKNNKATGNNKNNKNNKCACTTGGATAAAA GCATTAA | M317, L318, V320, C321, H322 |
|  | UreB-2R7 | GGTGATTGAGAAAATCCCCA |  |
|  | UreB-2R8F | GTTCAGTTCGCTGATTCANNKATCCGCCCTC | R338 |
|  | UreB-2R8P | CCCATAGCTTGAGAGTCAGAAC |  |
|  | UreB-2F8 | TGGGGATTTTCTCAATCACCNNKNNKNNKNNKNNKNNKNNKGGTCGTGTGGG TGAAGTTAT | S360, S361, D362,  S363, Q364, A365,  M366 |
|  | UreB-2R8 | ATGGTGCTGGCCGGCCTGGCCGCGGCCAAA | C-termina |
